# Impact of hepatitis C virus point-of-care RNA viral load testing compared with laboratory-based testing on uptake of RNA testing and treatment, and turnaround times: a systematic review and meta-analysis

**DOI:** 10.1016/S2468-1253(22)00346-6

**Published:** 2023-01-24

**Authors:** Adam Trickey, Emmanuel Fajardo, Daniel Alemu, Andreea Adelina Artenie, Philippa Easterbrook

**Affiliations:** a Population Health Sciences, University of Bristol, Bristol, UK; b Department of Global HIV, Hepatitis and STI Programmes, World Health Organization, Geneva, Switzerland

## Abstract

**Background:**

Point-of-care (POC) hepatitis C virus (HCV) RNA nucleic acid test viral load assays are being used increasingly as an alternative to centralised, laboratory-based standard-of-care (SOC) viral load assays to reduce loss to follow-up. We aimed to evaluate the impact of using POC compared with SOC approaches on uptake of HCV RNA viral load testing and treatment, and turnaround times from testing to treatment along the HCV care cascade.

**Methods:**

We searched PubMed, Embase, and Web of Science for studies published in English between Jan 1, 2016, and April 13, 2022. We additionally searched for accepted conference abstracts (2016−20) not identified in the main search. The contacts directory of the WHO Global Hepatitis Programme was also used to solicit additional studies on use of POC RNA assays. We included studies if they evaluated use of POC HCV RNA viral load with or without a comparator laboratory-based SOC assay, and had data on uptake of viral load testing and treatment, and turnaround times between these steps in cascade. We excluded studies with a sample size of ten or fewer participants. The POC studies were categorised according to whether the POC assay was based onsite at the clinic, in a mobile unit, or in a laboratory. Studies using the POC assay or comparator SOC assays were further stratified according to four models of care: whether HCV testing and treatment initiation were performed in the same or different site, and on the same or a different visit. The comparator was centralised, laboratory-based HCV RNA SOC assays. For turnaround times, we calculated the weighted median of medians with 95% CIs. We analysed viral load testing and treatment uptake using random-effects meta-analysis. The quality of evidence was rated using the GRADE framework. This study is registered with PROSPERO, CRD42020218239.

**Findings:**

We included 45 studies with 64 within-study arms: 28 studies were in people who inject drugs, were homeless, or both; four were in people incarcerated in prison; nine were in the general or mixed (ie, includes high-risk groups) populations; and four were in people living with HIV. All were observational studies. The pooled median turnaround times between HCV antibody test and treatment initiation was shorter with onsite POC assays (19 days [95% CI 14–53], ten arms) than with either laboratory-based POC assays (64 days [64–64], one arm) or laboratory-based SOC assays (67 days [50–67], two arms). Treatment uptake was higher with onsite POC assays (77% [95% CI 72–83], 34 arms) or mobile POC assays (81% [60–97], five arms) than with SOC assays (53% [31–75], 12 arms); onsite and mobile POC assay *vs* SOC assay p=0·029). For POC and SOC arms, higher RNA viral load testing uptake was seen with the same-site models for testing and treatment than with different-site models (all within-category p≤0·0001). For onsite and mobile POC arms, there was higher treatment uptake for same-site than different-site models (within-category p<0·0001). Four studies had direct within-study POC versus SOC comparisons for RNA viral load testing uptake (pooled relative risk 1·11 [95% CI 0·89–1·38]), and there were ten studies on treatment uptake (1·32 [1·06–1·64]). Overall, the quality of evidence was rated as low.

**Interpretation:**

Compared with use of laboratory-based SOC HCV viral load testing, the use of POC assays was associated with reduced time from antibody test to treatment initiation and increased treatment uptake. The effect of POC viral load testing is greatest when positioned within a simplified care model in which testing and treatment are provided at the same site, and, where possible, on the same day. POC HCV RNA viral load testing is now recommended in WHO guidelines as an alternative strategy to laboratory-based viral load testing.

**Funding:**

Unitaid.

**Research in context**

**Evidence before this study**

Point of care (POC) viral load assays are recommended by WHO for diagnosis and monitoring of infectious diseases, including tuberculosis, HIV early infant diagnosis, and HIV treatment monitoring, on the basis of high-quality evidence from randomised controlled trials. These trials showed that POC molecular testing for HIV early infant diagnosis was associated with faster result delivery time and antiretroviral therapy initiation in HIV-positive children, and for HIV viral load monitoring, faster return of results to patients and clinicians, and time to clinical action for elevated viral load than standard of care (SOC). There has been limited data on the impact of use of POC viral load assays on promoting access to hepatitis C virus (HCV) viral load testing and treatment. Many of the same benefits in use of POC platforms with HIV and tuberculosis might apply to HCV, despite differences in the care pathways. WHO recently undertook a systematic review and meta-analysis on the diagnostic performance of POC HCV viral load assays compared with laboratory-based SOC viral load testing. Overall, pooled sensitivity was 99% (95% CI 98–99) and pooled specificity was 99% (99–100). This high diagnostic performance was also observed across all settings and populations, and with use of different manufacturer's POC platforms and specimen types. There has been no previous systematic review evaluating the impact of POC HCV viral load assays on turnaround times and uptake of viral load testing and treatment.

**Added value of this study**

We undertook a systematic review and meta-analysis to evaluate the impact of using POC HCV RNA assays compared with centralised, laboratory-based SOC approaches on uptake of HCV viral load testing and treatment, and turnaround times to treatment initiation in HCV-antibody seropositive people. We included 45 observational studies. The pooled median turnaround time between HCV antibody test and treatment initiation was shorter with onsite POC assays (weighted median of medians 19 days [95% CI 14–53]) than with either laboratory-based POC assays (64 days [64–64]) or laboratory-based SOC assays (67 days [50–67]). Treatment uptake was higher with onsite POC assays (77% [95% CI 72–83]) or with mobile POC assays (81% [60–97]) than with SOC assays (53% [31–75]). Among the studies that had both POC and SOC comparator arms within the same study, the pooled relative risk for viral load uptake was 1·11 (95% CI 0·89–1·38) for POC versus SOC assays in four studies and the pooled relative risk for treatment uptake was 1·32 (1·06–1·64) in ten studies.

**Implications of all the available research**

This evidence base has informed new WHO recommendations for adoption of POC HCV viral load testing as an alternative approach to laboratory-based platforms for diagnosis of HCV viraemic infection. This is especially relevant for promoting linkage to care for hard to reach or marginalised populations at high risk of loss to follow-up, and at decentralised HCV testing and treatment sites that might include harm-reduction services, primary or secondary care clinics, prisons, and HIV clinics.

## Introduction

Chronic hepatitis C virus (HCV) infection is a major global public health problem and cause of liver disease, with the highest burden in low-income and middle-income countries (LMICs). In 2019, there were an estimated 290 000 HCV-related deaths.^1^ In 2016, WHO launched the Global Health Sector Strategy for Viral Hepatitis 2016–2021,^2^ with a goal of eliminating viral hepatitis B and hepatitis C by 2030. Good progress has been made, with an estimated 9·4 million people with chronic HCV infection treated using direct-acting antiviral therapy between 2015 and 2019.^1^ However, as of 2019, there were still 58 million people with chronic HCV infection, and only 20% of those infected worldwide had been diagnosed and 13% treated.^1^ To address this gap and achieve the WHO targets for elimination will require a substantial scale-up of testing and treatment using simplified service delivery models. A 2021 WHO-led systematic review provided a strong evidence base for the effectiveness of full decentralisation of testing and treatment and integration with other services at harm-reduction sites, supported through task sharing, especially among people who inject drugs.^3^

The recommended diagnostic strategy for chronic HCV infection is initial screening with an HCV antibody serological assay, followed by laboratory-based molecular viral load testing for HCV RNA, to confirm the presence of HCV viraemia and need for treatment.^4^, ^5^ However, access to laboratory-based viral load testing remains limited in many LMICs. As a result, many people with chronic HCV infection are never linked to care. HCV viral load assays performed on point-of-care (POC) devices outside the laboratory are being increasingly used as an alternative testing approach, especially in facilities caring for populations with high rates of loss to care and follow‑up.^4^, ^5^ POC devices can also be used for a test of cure after completing treatment, in addition to same-day diagnosis of HCV viraemic infection.^6^

Although there is now high-quality evidence of the clinical impact of POC assays for HIV viral load monitoring,^7^ early infant diagnosis of HIV,^8^ and diagnosis of tuberculosis,^9^, ^10^ data on their impact on promoting access to HCV viral load testing and treatment are scarce. We undertook a systematic review and meta-analysis to evaluate the impact of using POC HCV viral load assays compared with centralised, high-throughput, laboratory-based standard-of-care (SOC) approaches on uptake of HCV viral load testing and treatment, and turnaround times to treatment initiation in HCV-antibody seropositive people.

## Methods

### Search strategy and selection criteria

For this systematic review and meta-analysis, we searched PubMed, Embase, and Web of Science for observational and randomised controlled trials that used POC HCV viral load assays with or without a comparator laboratory-based SOC assay and contained data on outcomes across the HCV cascade of care and turnaround times. The PICO (population, intervention, comparator, and outcome) question is described in the appendix (pp 3–4). The search was carried out on Sept 23, 2020, on studies in English published from Jan 1, 2016 (the date of WHO pre-qualification of the first POC HCV viral load assay).^11^ A further updated search was done on April 13, 2022, to identify additional studies published between Sept 24, 2020, and April 13, 2022. In addition, we searched for accepted conference abstracts (2016−20) from the International Liver Conference, the International Network on Hepatitis in Substance Users symposia, and the International Viral Hepatitis Elimination Meeting that were not identified in the main search. The contacts directory of the WHO Global Hepatitis Programme was also used to solicit additional studies (completed or ongoing) on use of POC RNA assays from relevant parties, such as the manufacturers of the assays, Médecins Sans Frontières, and the Foundation for Innovative New Diagnostics. The reference lists of all retrieved articles, including review articles, identified during the initial search were also screened for citations of other relevant studies. We reviewed all final included abstracts and full papers, and any duplicate reports were excluded. The following information is provided in the appendix: the list of POC HCV viral load assays included (p 5), search terms (p 6), and further details on the search strategy (p 7).

Studies were included if they had evaluated use of POC HCV viral load with or without a centralised, laboratory-based SOC comparator assay, and had data on uptake of viral load testing and treatment across the care cascade, turnaround times between different steps, or both. Studies with a sample size of ten or fewer participants for the largest denominator were excluded. The appendix (p 8) gives further information about selection criteria.

For the main search and for studies identified through WHO partners, AT and EF conducted the search and independently evaluated the articles (first the titles and abstracts and then the full texts of those selected from the title and abstract screening) to determine the study eligibility, and PE reviewed the final selection and arbitrated on differences between the primary reviews. Manuscript references were checked by AT and DA, with EF arbitrating selection differences.

The main intervention group was use of a POC HCV RNA assay (POC group), and the comparator group was use of a centralised, laboratory-based, high-throughput SOC HCV RNA assay (SOC group). The POC HCV viral load assay intervention was further categorised according to whether the POC assay was used onsite (POC onsite) or in a mobile unit (POC mobile, defined as units that were not fixed to a particular site). POC assays that were undertaken at a centralised hub based on specimens sent from different clinic sites were classified as a laboratory-based POC assay.

All population types were included and were grouped into the following categories: people who inject drugs, were homeless, or both; the general or mixed (ie, includes high-risk groups) population; people incarcerated in prison; and people living with HIV. Only two studies included homeless people, and this population was grouped with people who inject drugs because one of the studies also reported a high proportion of injecting drug use, and there would have been insufficient data for an analysis of people who are homeless but do not inject drugs. The corresponding settings for these different populations were harm-reduction sites for people who inject drugs, homeless shelters for homeless people, primary health clinics or district hospitals for the general or mixed populations, prisons for people who were incarcerated, and HIV clinics for people living with HIV*.*

For both the POC and SOC groups, studies were further classified according to four models of care: whether initial HCV testing and treatment initiation were performed in the same or different site, and on the same or a different visit. The categories were testing and treatment initiation at the same site and on the same visit; testing and treatment initiation at the same site but treatment initiation on a different visit; testing at one site with referral to another site for treatment initiation on the same visit; and testing and treatment initiation at different sites and on different visits.

### Data analysis

For each study, data were extracted by AT and DA using a standardised data extraction form and checked by EF. Descriptive data extracted were country, setting, population type, population characteristics (mean or median age and percentage female), study design, and publication type. Study authors were contacted where necessary to clarify results or provide further or updated information and data.

The key outcomes were turnaround times in days from HCV antibody test to viral load test, viral load sample collection to testing, viral load test to results being made available to patient, viral load test to treatment initiation, and overall HCV antibody test to treatment initiation, in addition to uptake of HCV viral load testing and treatment.

Data on the median number of days between key steps in the cascade were pooled and presented as weighted median of medians for the POC groups (onsite, mobile, and laboratory based) compared with the SOC group, further stratified by four categories of model of care.^12^

The denominator for each step of the cascade was the number of participants who were eligible for this step (eg, for viral load testing, the denominator was the number of participants who were HCV antibody positive). These denominators were used to weight the turnaround time analyses. For estimation of the proportion of participants initiating treatment, we used the number of HCV RNA-positive individuals as the denominator, rather than attendance at pre-treatment assessment visits, which were not undertaken in all studies. Data on uptake of RNA viral load testing and treatment were pooled for POC groups compared with the SOC groups, and further stratified by the four categories of model of care using random-effects meta-analysis, with 95% CIs based on the exact binomial (Clopper-Pearson) method. We used the Freeman-Tukey double arcsine transformation to stabilise the variances. In a post-hoc analysis, pooled uptake percentages of viral load and treatment for each population group were stratified by country income status using World Bank 2021 definitions: low-income and middle-income, or high-income.

For the studies that had both a POC group and a SOC comparator group (historic or concurrent) within the same study, we compared outcomes in studies that had the same population and clinical service delivery model. The relative risk of viral load testing and treatment uptake were calculated and pooled in a random-effects meta-analysis.

EF and AT assessed the risk of bias for each study using a previously published and modified tool used for observational studies that report binary outcomes based on tools developed by Hoy and colleagues and the ROBINS-I tool,^13^, ^14^, ^15^ with AAA arbitrating disagreements. The quality of evidence was assessed using the Grading of Recommendations Assessment, Development and Evaluation framework,^16^ considering the risk of bias, consistency of results, directness of the evidence, precision of the estimates, and reporting bias.

We used a regression-based Egger test to assess publication bias for each outcome.^17^ The *I*^2^ statistic was used to measure heterogeneity between POC and SOC groups and within four service delivery model categories.^18^ Analyses were performed in Stata (version 16.1) and R (version 4.2.1).

The study is registered with PROSPERO, CRD42020218239.

### Role of the funding source

The funder of the study had no role in study design, data collection, data analysis, data interpretation, or writing of the report.

## Results

**Figure 1:**
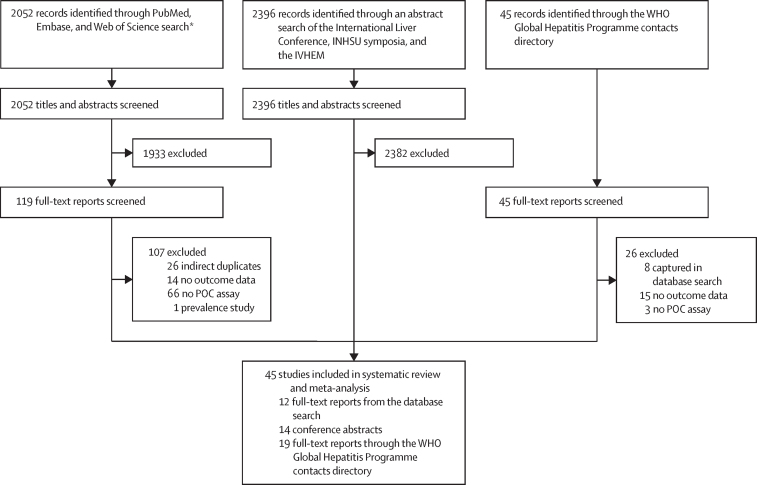
Study selection AASLD=American Association for the Study of Liver Diseases. APASL=Asia-Pacific Association for the Study of the Liver. INHSU=International Network on Hepatitis in Substance Users. IVHEM=International Viral Hepatitis Elimination Meeting. POC=point-of-care. *AASLD and APASL conference abstracts were captured in Embase database search.

45 studies, including 64 study arms, were included in the systematic review and meta-analysis (figure 1). Table 1 summarises key features, with additional details including all outcomes provided in the appendix (pp 15–35). Of the 45 studies, 28 were in people who inject drugs, were homeless, or both; nine were in the general population or mixed populations; four were in people incarcerated in prison; and four were in people living with HIV (Table 1, Table 2). 24 (53%) studies were from high-income countries, 19 (42%) from middle-income countries, and two (4%) from low-income countries (appendix p 14). All were observational studies. Two additional very small studies with 11 HCV-positive people^70^, ^71^ identified in the 2020−22 updated search were not included because they would not have affected the study findings or conclusions.

**Table 1:** Characteristics of 45 included studies

	**Study population**	**Setting**	**City or region, country**	**Design**	Study source*	**Number of study arms**	**Study group or subgroup**†**(model of care**‡**)**	**Risk of bias**§
Bajis et al (2019)^19^	People who were homeless	Hostel	Sydney, NSW, Australia	Prospective cohort	Main database search	1	Onsite POC (same site, different visit)	High
London Joint Working Group on Substance Use and Hepatitis C (2020)^20^	People who were homeless	Temporary accommodation	London, UK	Prospective observational	WHO contacts directory	1	Mobile POC (different site, different visit)	Some
Chevaliez et al (2020)^21^	People who inject drugs (ever injecting)	Drug treatment centres and drug consumption room	Paris, France	Prospective observational	Main database search	1	Onsite POC (different site, different visit)	Some
Lens et al (2020)^22^	People who inject drugs (active injecting)	Harm-reduction centre	Barcelona, Spain	Prospective observational	Conference abstract search	1	Onsite POC (same site, different visit)	Low
Lazarus et al (2020)^23^	People who inject drugs (active or ever injecting not assessed)	Mobile van	Copenhagen, Denmark	Prospective observational	Conference abstract search	1	Mobile POC (different site, different visit)	Some
Rogers et al (2020)^24^	People who inject drugs (active or ever injecting not assessed)	Pharmacies	Leicestershire, UK	Prospective observational	Conference abstract search	1	Onsite POC (different site, different visit)	High
Antonini et al (2018)^25^	People who inject drugs (ever injecting)	Addiction centre	Paris, France	Prospective observational	Conference abstract search	1:	Onsite POC (different site, different visit)	High
Remy et al (2019)^26^	People who inject drugs (ever injecting)	Mobile hepatitis team	Perpignan, France	Prospective observational	Main database search	1	Mobile POC (same site, different visit)	Low
Bajis et al (2020)^27^	People who inject drugs (ever injecting)	Drug and alcohol treatment sites, needle and syringe provision site, supervised injecting centre, community health centres	New South Wales, Queensland, South Australia, Australia	Prospective cohort	Main database search	2	Onsite POC (same site, different visit); SOC (same site, different visit)	High
Williams et al (2019)^28^	People who inject drugs (active or ever injecting not assessed)	Primary care clinics	Melbourne, VIC, Australia	Prospective cohort	Main database search	1	Onsite POC (same site, different visit)	Some
Valencia et al (2021),^29^ Ryan et al (2021)^30^	People who inject drugs and were homeless (active or ever injecting not assessed)	Mobile screening unit	Madrid, Spain	Prospective observational	Conference abstract search	1	Mobile POC (different site, same visit)	Low
Saludes et al (2020)^31^	People who inject drugs (injecting past 6 months)	Drug consumption room	Catalonia, Spain	Prospective observational	Main database search	2	Onsite POC (same site, different visit); SOC (same site, different visit)	Some
Schürch et al (2020)^32^	People who inject drugs (patients on opioid agonist therapy)	Opiate substitution treatment site	Aargau, Switzerland	Retrospective cohort	Main database search	2	Onsite POC (same site, different visit); SOC (same site, different visit)	High
Martel-Laferrière et al (2019),^33^ Martel-Laferrière et al (2022)^34^	People who inject drugs (injecting past year)	Addiction medicine clinic	Quebec, Canada	Prospective observational	Main database search	2	Onsite POC (different site, different visit); SOC (different site, different visit)	Some
Feld et al (2019)^35^	People who inject drugs (active or ever injecting not assessed)	Supervised consumption service	Toronto, ON, Canada	Prospective observational	Main database search	1	Onsite POC (same site, different visit)	Low
Thingnes et al (2019),^36^ Midgard et al (2022)^37^	People who inject drugs (active or ever injecting not assessed)	Mobile health service	Oslo, Norway	Prospective observational	Conference abstract search	1	Mobile POC (same site, different visit)	Low
Stone et al (2021)^38^	People who inject drugs (vast majority currently injecting)	Drug services	South Yorkshire, UK	Prospective observational	WHO contacts directory	1	Onsite POC (same site, same visit)	Low
Gutierrez, Médecins Sans Frontières (2019)^39^	People who inject drugs (active or ever injecting not assessed)	Community-based drop-in centre	Mafalala, Mozambique	Retrospective observational	WHO contacts directory	1	Onsite POC (different site, different visit)	High
Butsashvili et al (2019)^40^	People who inject drugs (active or ever injecting not assessed)	Opiate substitution treatment and needle and syringe provision centres	Tbilisi, Zugdidi, and Batumi, Georgia	Retrospective observational	Conference abstract search	1	Onsite POC (same site, different visit)	High
Thaung et al (2021)^41^	People who inject drugs (active or ever injecting not assessed)	Clinical facilities	Kachin, Myanmar	Prospective observational	Conference abstract search	3	Onsite POC (same site, different visit); laboratory-based POC (same site, different visit); laboratory-based POC (same site, different visit)	Some
Ramachandran et al (2019)^42^	People who inject drugs (ever injecting)	Harm reduction sites	Manipur, India	Prospective observational	Conference abstract search	3	Onsite POC (same site, different visit); onsite POC (different site, different visit); onsite POC (different site, different visit)	Low
Japaridze et al (2020),^43^ Shilton et al (2022)^44^	People who inject drugs (active or ever injecting not assessed)	Harm-reduction sites	Tbilisi, Batumi, Kutaisi, Zugdidi, Rustavi, and Gori, Georgia	Cluster non-randomised intervention	Conference abstract search	3	Onsite POC (different site, different visit); SOC (different site, different visit); SOC (different site, different visit)	Low
Hellard (2020),^45^ Draper (2021)^46^	People who inject drugs (active or ever injecting not assessed)	Harm-reduction sites	Yangon, Myanmar	Prospective observational	Main database search	1	Onsite POC (same site, different visit)	Low
London Joint Working Group on Substance Use and Hepatitis C (2018),^47^ London Joint Working Group on Substance Use and Hepatitis C (2019)^48^	People who inject drugs (active injecting)	Needle and syringe provisions at pharmacies	London, UK	Retrospective observational	WHO contacts directory	2	Onsite POC (different site, different visit); SOC (different site, different visit)	High
SOS Hépatites (2019)^49^	People who inject drugs (active or ever injecting not assessed)	Motor home	Burgundy, France	Prospective observational	WHO contacts directory	1	Mobile POC (same site, same visit)	High
Morris et al (2020)^50^	People who inject drugs and were homeless (active or ever injecting not assessed)	Hostels	West Midlands, UK	Prospective observational	WHO contacts directory	1	Onsite POC (different site, different visit)	Low
Wansom et al (2021)^51^	People who inject drugs (ever injecting)	Community drop-in centres	Bangkok, Chiang Mai, Songkhla, and Narathiwat, Thailand	Prospective observational	WHO contacts directory	1	Onsite POC (same site, different visit)	Low
Sonderup (2021)^52^	People who inject drugs (active or ever injecting not assessed)	Opiate substitution treatment centre	Pretoria, South Africa	Prospective observational	WHO contacts directory	1	Onsite POC (same site, same visit)	Some
Agwuocha et al (2019)^53^	General population	Tertiary hospital, with some primary and secondary facilities	Nasarawa, Nigeria	Retrospective observational	WHO contacts directory	1	Laboratory-based POC (different site, different visit)	High
Médecins Sans Frontières (2020)^54^	General population	District hospital	Uttar Pradesh, India	Prospective observational	WHO contacts directory	1	Onsite POC (same site, different visit)	Some
Walker et al (2020)^55^	General population and high-risk groups	National hospital	Phnom Penh, Cambodia	Prospective observational	WHO contacts directory	2	Onsite POC (same site, different visit); SOC (same site, different visit)	Some
Khalid et al (2020)^56^	General population and high-risk groups	Primary health-care clinic	Karachi, Pakistan	Retrospective cohort	WHO contacts directory	2	Onsite POC (same site, different visit); SOC (same site, different visit)	High
Qureshi et al (2017)^57^	General population	Private clinic and civil society organisation	Karachi, Pakistan	Prospective observational	WHO contacts directory	1	Onsite POC (same site, different visit)	High
Qureshi et al (2019)^58^	General population and high-risk groups	Visiting dwellings in a slum	Islamabad, Pakistan	Retrospective observational	Conference abstract search	1	Onsite POC (same site, different visit)	High
Hamid et al (2021),^59^ Abid et al (2021)^60^	General population and high-risk groups	Community screening camps	Karachi, Pakistan	Prospective observational	WHO contacts directory	2	Laboratory-based POC (same site, different visit); SOC (same site, different visit)	Some
Shiha et al (2020)^61^	General population	Non-governmental organisation building and governmental state office	Dakahlia and Cairo, Egypt	Prospective observational	Main database search	2	Onsite POC (same site, same visit); onsite POC (same site, same visit)	Some
Zhang et al (2021)^62^	General population	Rural health centres and district hospital	Battambang province, Cambodia	Prospective observational	WHO contacts directory	1	Laboratory-based POC (different site, different visit)	Some
Mohamed et al (2020)^63^	People incarcerated in prison	Men's remand prison	London, UK	Prospective observational	Main database search	2	Onsite POC (same site, different visit); SOC (same site, different visit)	Low
Davies et al (2020)^64^	People incarcerated in prison	Men's remand prison	Swansea, UK	Prospective observational	Conference abstract search	2	Onsite POC (same site, different visit); SOC (same site, different visit)	Some
Llerena et al (2020),^65^ Cabezas et al (2021)^66^	People incarcerated in prison	Centre for social insertion (non-custodial sentences)	Santander, Spain	Prospective observational	Conference abstract search	1	Onsite POC (same site, same visit)	Low
Ustianowski et al (2020)^67^	People incarcerated in prison	Women's prison	Manchester, UK	Retrospective observational	WHO contacts directory	2	Onsite POC (same site, different visit); SOC (same visit, different visit)	High
Shilton et al (2020)^68^	People living with HIV	Antiretroviral therapy centres at district hospitals	Punjab, India	Prospective observational	Conference abstract search	2	Onsite POC (same site, different visit); laboratory-based POC (same visit, different visit)	Some
Nguyen et al (2020)^69^	People living with HIV	Specialised HIV centre in primary care clinic	Maputo, Mozambique	Prospective observational	WHO contacts directory	1	Onsite POC (same site, different visit)	High
Nguyen A, personal communication	People living with HIV	National HIV centre	Mykolaiv, Ukraine	Prospective observational	WHO contacts directory	1	Onsite POC (different site, different visit)	High
Nguyen A, personal communication	People living with HIV	HIV centre	Dawei, Myanmar	Prospective observational	WHO contacts directory	1	Onsite POC (same site, different visit)	Some

HCV=hepatitis C virus. POC=point of care. SOC=standard of care.

* Study source refers to how study was identified: through the main database search strategy (PubMed, Embase, and Web of Science), from the conference abstract search (the International Liver Conference, the International Network on Hepatitis in Substance Users symposia, and the International Viral Hepatitis Elimination Meeting), or contacts directory the WHO Global Hepatitis Programme.

† Onsite POC refers to HCV RNA assays done onsite; mobile POC refers to assays done in mobile units; laboratory-based POC refers to assays done in one centralised laboratory using a POC device with samples taken at different clinical sites; and SOC refers to centralised, laboratory-based, high-throughput assays.

‡ Same site, same visit refers to testing and treatment initiation at the same site and on the same visit; same site, different visit refers to testing and treatment initiation at the same site but treatment initiation on a different visit; different site, same visit refers to testing at one site with referral to another site for treatment initiation on the same visit; and different site, different visit refers to testing and treatment initiation at different sites and on different visits.

§ Risk of bias was assessed for each study using a previously published and modified risk of bias tool used for observational studies that report binary outcomes based on tools by Hoy and colleagues and the ROBINS-I tool.^13^, ^14^, ^15^

**Table 2:** Study characteristics

		**Overall (n=45)**	**People who inject drugs, were homeless, or both (n=28)**	General and mixed*populations (n=9)	**People incarcerated in prison (n=4)**	**People living with HIV (n=4)**
Studies from LMICs (from World Bank 2021)†		21 (47%)	8 (29%)	9 (100%)	0	4 (100%)
Studies from WHO region						
	Africa	4 (9%)	2 (7%)	1 (11%)	0	1 (25%)
	Americas	2 (5%)	2 (7%)	0 (0%)	0	0
	Eastern Mediterranean	5 (11%)	0	5 (56%)	0	0
	European	22 (50%)	17 (61%)	0 (0%)	4 (100%)	1 (25%)
	South-East Asia	7 (16%)	4 (14%)	1 (11%)	0	2 (50%)
	Western Pacific	5 (11%)	3 (11%)	2 (22%)	0	0
Studies with two or more arms‡		16 (36%)	8 (29%)	4 (44%)	3 (75%)	1 (25%)
Studies with SOC comparator arms		12 (27%)	6 (23%)	3 (38%)	3 (75%)	0
Studies containing both POC arms and SOC comparator arms		11 (24%)	6 (23%)	2 (22%)	3 (75%)	0

Data are n (%). LMIC=low-income and middle-income country. POC=point of care. SOC=standard of care.

* General populations that also contain high-risk groups.

† LMIC as classified by the World Bank in 2021; for this review, the LMICs where studies took place were Cambodia, Egypt, Georgia, India, Mozambique, Myanmar, Nigeria, Pakistan, South Africa, Thailand, and Ukraine.

‡ 30 studies have one arm, 13 studies have two arms, and three studies have three arms.

Table 3 summarises the pooled characteristics of the 45 studies with 64 arms (51 were in the POC group and 13 were in the laboratory-based SOC group), comprising 27 364 people who had an HCV viral load test. All of the POC viral load assays were GeneXpert (Cepheid, Sunnyvale, CA, USA), except one study that used Genedrive (Epistem, Manchester, UK).^52^ Overall, the POC RNA viral load test was done onsite in 39 study arms, in a mobile unit in six arms, and in a laboratory in six arms. 12 studies (13 arms) had a SOC assay comparator, including one study (HEAD-start Georgia) with two SOC arms. Most comparator arms were based on historical data, and one was based on concurrent data.^44^ Of the 51 POC arms and 13 SOC arms, 32 (63%) and seven (54%), respectively, comprised people who inject drugs, were homeless, or both (p=0·30), and 24 (47%) and eight (62%), respectively, were in high-income countries (p=0·35).

**Table 3:** Model-of-care characteristics of POC assay arms and SOC comparator assay arms

			**Overall (n=45)**	**People who inject drugs, were homeless, or both (n=28)**	General and mixed*populations (n=9)	**People incarcerated in prison (n=4)**	**People living with HIV (n=4)**
Total number of POC assay and SOC comparator assay arms (studies)			64 (45)	39 (28)	13 (9)	7 (4)	5 (4)
Total number of POC assay arms (studies) available (onsite or mobile)			45 (42)	30 (28)	7 (6)	4 (4)	4 (4)
	Onsite POC		39 (37)	24 (23)	7 (6)	4 (4)	4 (3)
		Same site, same visit	5 (4)	2 (2)	2 (1)	1 (1)	0
		Same site, different visit	23 (23)	12 (12)	5 (5)	3 (3)	3 (3)
		Different site, different visit	11 (10)	10 (9)	0	0	1 (1)
	Mobile POC		6 (6)	6 (6)	0	0	0
		Same site, same visit	1 (1)	1 (1)	0	0	0
		Same site, different visit	2 (2)	2 (2)	0	0	0
		Different site, same visit	1 (1)	1 (1)	0	0	0
		Different site, different visit	2 (2)	2 (2)	0	0	0
	Laboratory-based POC		6 (5)	2 (1)	3 (3)	0	1 (1)
		Same site, different visit	4 (3)	2 (1)	1 (1)	0	1 (1)
		Different site, different visit	2 (2)	0	2 (2)	0	0
Total comparator laboratory-based SOC assay arms (studies) available			13 (12)	7 (6)	3 (3)	3 (3)	0
	Same site, different visit		9 (9)	3 (3)	3 (3)	3 (3)	0
	Different site, different visit		4 (3)	4 (3)	0	0	0

Data are number of arms (number of studies). Onsite POC refers to HCV RNA assays done onsite; mobile POC refers to assays done in mobile units; laboratory-based POC refers to assays done in one centralised laboratory using a POC device with samples taken at different clinical sites; and SOC refers to centralised, laboratory-based, high-throughput assays. Models of care: same site, same visit refers to testing and treatment initiation at the same site and on the same visit; same site, different visit refers to testing and treatment initiation at the same site but treatment initiation on a different visit; different site, same visit refers to testing at one site with referral to another site for treatment initiation on the same visit; and different site, different visit refers to testing and treatment initiation at different sites and on different visits. POC=point of care. SOC=standard of care.

* General populations that also contain high-risk groups.

The most common model of care for the 45 POC (onsite and mobile) arms (in around 50%) and the 13 laboratory-based SOC arms was same site for testing and treatment, but at different visits (table 3). 41 arms had turnaround times for at least one step of the HCV cascade, and all but one of the 64 arms had data on uptake of viral load testing, treatment, or both (table 4).

**Table 4:** Available outcomes across the HCV care cascade

	**Overall (n=45)**	**People who inject drugs, were homeless, or both (n=28)**	General and mixed*populations (n=9)	**People incarcerated in prison (n=4)**	**People living with HIV (n=4)**
**Cascade outcomes available for the 45 HCV POC assay arms (onsite or mobile)**					
HCV antibody tested†	5 (5)	2 (2)	0	3 (3)	0
HCV RNA tested	28 (25)	18 (16)	4 (3)	3 (3)	3 (3)
Post-RNA assessment	13 (12)	10 (10)	43 (2)	0	0
Started treatment	39 (36)	24 (22)	7 (6)	4 (4)	4 (4)
SVR12 results available	23 (21)	15 (13)	4 (4)	1 (1)	3 (3)
SVR12 obtained	24 (22)	16 (14)	64 (4)	1 (1)	3 (3)
Cascade turnaround time data available	29 (26)	19 (17)	5 (4)	4 (4)	1 (1)
**Cascade outcomes available for the six laboratory-based POC assay arms**					
HCV antibody tested†	0	0	0	0	0
HCV RNA tested	4 (4)	0	3 (3)	0	1 (1)
Post-RNA assessment	1 (1)	0	1 (1)	0	0
Started treatment	5 (4)	2 (1)	2 (2)	0	1 (1)
SVR12 results available	5 (4)	2 (1)	2 (2)	0	1 (1)
SVR12 obtained	5 (4)	2 (1)	2 (2)	0	1 (1)
Cascade turnaround time data available	4 (3)	2 (1)	1 (1)	0	1 (1)
**Cascade outcomes available for the 13 laboratory-based comparator SOC assay arms**					
HCV antibody tested†	3 (3)	1 (1)	0	2 (2)	0
HCV RNA tested	6 (6)	1 (1)	2 (2)	3 (3)	0
Post-RNA assessment	3 (3)	1 (1)	1 (1)	1 (1)	0
Started treatment	12 (11)	6 (5)	3 (3)	3 (3)	0
SVR12 results available	4 (3)	2 (2)	1 (1)	1 (1)	0
SVR12 obtained	5 (4)	3 (2)	1 (1)	1 (1)	0
Cascade turnaround time data available	8 (7)	4 (3)	2 (2)	2 (2)	0

Data are number of arms (number of studies). HCV=hepatitis C virus. POC=point of care. SOC=standard of care. SVR12=sustained virological response 12 weeks after treatment.

* General populations that also contain high-risk groups.

† The denominator for HCV antibody tested was the population entering prison for five arms (three POC and two SOC), the population in a harm-reduction cohort for two arms (one POC and one SOC), and the population attending a harm-reduction centre for one arm (POC).

The appendix summarises for each study the different POC and SOC groups, model-of-care category, and available outcome data for viral load testing and treatment uptake (pp 30–33) and turnaround times (pp 34–35).

Overall, there were clear differences in the overall pooled turnaround time reported between HCV antibody testing and treatment initiation between arms with POC assays versus those with laboratory-based SOC assays. Time between HCV antibody testing and treatment initiation was shorter for the ten onsite POC arms (weighted median of medians 19 days [95% CI 14–53]) than for the one laboratory-based POC assay arm (64 days [64–64]) and the two arms that used SOC assays (67 days [50–67]; table 5). The one small study (n=44) that used a POC assay in a mobile unit reported a median of 0 days (95% CI 0–0) between HCV antibody testing and treatment initiation (table 5).

**Table 5:** Weighted medians of the median time in days between steps in the HCV cascade of care with use of POC compared with laboratory-based SOC HCV RNA assays, stratified according to model of care

			**HCV antibody test to RNA test**			**RNA sample collection to test**			**RNA test to results made available**			**RNA test to treatment start**			HCV antibody test to treatment start*		
			Arms	Participants	Weighted† median (95% CI) of the median days‡ between cascade steps	Arms	Participants	Weighted† median (95% CI) of the median days‡ between cascade steps	Arms	Participants	Weighted† median (95% CI) of the median days‡ between cascade steps	Arms	Participants	Weighted† median (95% CI) of the median days‡ between cascade steps	Arms	Participants	Weighted† median (95% CI) of the median days‡ between cascade steps
All study groups			22	14 005	0 (0–0)	14	9963	2 (0–2)	19	12 017	4 (0–4)	28	7497	14 (13–57)	14	5640	53 (17–64)
POC assay groups																	
	Onsite		15	6734	0 (0–0)	9	4421	0 (0–1)	13	4938	0 (0–0.01)	17	4010	13 (10–14)	10	3070	19 (14–53)
		Same site, same visit	4	302	0 (0–0)	5	329	0 (0–0)	5	809	0 (0–0)	5	186	0 (0–0)	4	177	0 (0–0)
		Same site, different visit	7	4246	0 (0–0)	3	3472	0 (0–4)	6	3500	0 (0–0)	7	2214	13 (3–45)	2	1334	14 (14–53)
		Different site, different visit	4	2186	0 (0–5)	1	620	0 (0–0)	2	629	0 (0–0)	5	1610	14 (10–57)	4	1559	19 (17–57)
	Mobile		2	209	0 (0–0)	1	197	0 (0–0)	1	197	0 (0–0)	2	77	0 (0–1)	1	44	0 (0–0)
		Same site, same visit	1	12	0 (0–0)	0	0	NA	0	0	NA	0	0	NA	0	0	NA
		Same site, different visit	0	0	NA	0	0	NA	0	0	NA	1	33	1 (1–1)	0	0	NA
		Different site, same visit	1	197	0 (0–0)	1	197	0 (0–0)	1	197	0 (0–0)	1	44	0 (0–0)	1	44	0 (0–0)
		Different site, different visit	0	0	NA	0	0	NA	0	0	NA	0	0	NA	0	0	NA
	Laboratory based		2	4973	0 (0–1)	1	4211	2 (2–2)	1	762	0 (0–0)	4	2514	62 (5–62)	1	1835	64 (64–64)
		Same site, different visit	1	4211	0 (0–0)	1	4211	2 (2–2)	0	0	NA	3	1984	62 (7–62)	1	1835	64 (64–64)
		Different site, different visit	1	762	1 (1–1)	0	0	NA	1	762	0 (0–0)	1	530	5 (5–5)	0	00	NA
	Laboratory-based SOC assay group		3	2089	0 (0–1)	3	1134	6 (5–15)	4	6120	4 (4–9)	5	896	43 (31–107)	2	691	67 (50–67)
	Same site, different visit		1	1038	0 (0–0)	1	83	15 (15–15)	2	5069	4 (4–7)	2	184	107 (62–107)	0	0	NA
	Different site, different visit		2	1051	1 (0–1)	2	1051	5 (5–6)	2	1051	7 (7–9)	3	712	43 (31–266)	2	691	67 (50–67)

Onsite POC refers to HCV RNA assays done onsite; mobile POC refers to assays done in mobile units; laboratory-based POC refers to assays done in one centralised laboratory using a POC device with samples taken at different clinical sites; and SOC refers to centralised, laboratory-based, high-throughput assays. Models of care: same site, same visit refers to testing and treatment initiation at the same site and on the same visit; same site, different visit refers to testing and treatment initiation at the same site but treatment initiation on a different visit; different site, same visit refers to testing at one site with referral to another site for treatment initiation on the same visit; and different site, different visit refers to testing and treatment initiation at different sites and on different visits. HCV=hepatitis C virus. NA=not available. POC=point of care. SOC=standard of care.

* Note that the other components of the HCV cascade will not necessarily add up to the total for antibody test to treatment start as different numbers of studies are contributing data on different aspects of the cascade.

† Weighted by the number of participants in the step of the cascade for each arm.

‡ Information was available in different units from different studies, with some reporting in days and some reporting in hours and minutes. Hours and minutes have been converted to days.

After further stratification by model of care, pooled times between HCV antibody testing and treatment initiation were shorter for the simplest POC models: onsite with same site and same visit for testing and treatment (weighted median of medians 0 days [95% CI 0–0]; four arms) compared with onsite with same site but different visit for testing and treatment (14 days [14–53]; two arms) and onsite with different sites and visits for testing and treatment (19 days [17–57]; four arms; table 5). The longest turnaround times were seen with one laboratory-based POC study arm with testing and treatment at the same site but on different visits (weighted median of medians 64 days [95% CI 64–64]) and two laboratory-based SOC arms with testing and treatment at different sites on different visits (67 days [50–67]; table 5).

Most of the reduced turnaround time from HCV antibody testing to treatment initiation with use of POC assays was due to reduced turnaround time from viral load testing to treatment initiation (table 5). There were few differences between the POC groups and the SOC groups in the pooled turnaround times from HCV antibody testing to viral load testing. For the 54 (84%) of 64 arms in which antibody testing was performed, all used rapid antibody diagnostic tests, except for three (27%) of 11 SOC arms (appendix pp 15−29). However, there were reductions in turnaround times between RNA viral load sample collection and testing and between viral load testing and the results being made available to the patient.

Seven studies with data on turnaround times had at least one POC arm and a SOC arm, enabling direct within-study comparisons of turnaround times: three among people who inject drugs, were homeless, or both; two among the general and mixed populations; and two among people incarcerated in prison (appendix p 38). The study arms using POC assays had shorter turnaround times than those using SOC across all population groups. The within-study differences in pooled time between viral load sample collection and testing were 5·7 days (95% CI 2·0 to 9·4; three studies) shorter for the POC groups than the SOC groups, and 4·9 days (1·8 to 8·0; four studies) shorter between viral load testing and results being made available. There was no evidence of differences from antibody test to viral load test (difference 0·3 days [95% CI –3·5 to 4·2]; three studies), from viral load testing to treatment initiation (difference 3·3 days [–59·9 to 66·5]; five studies), or overall for antibody testing to treatment initiation (difference –1·8 days [–109·7 to 106·1]; two studies).

Overall, there was a high degree of heterogeneity across studies within each model-of-care category (*I*^2^>75%) for all outcomes and across all categories (appendix pp 50−57). The uptake of viral load testing when a POC viral load assay was onsite was 95% (95% CI [89–99]; 22 arms), 84% (43–100; six arms) when it was in a mobile unit, and 92% (68–100; four arms) for laboratory-based POC assays versus 82% (53–99; five arms) when using the SOC assay (table 6). There was no evidence of a difference in uptake of viral load testing regardless of whether the viral load assay was POC (onsite, mobile, or laboratory-based) or SOC (p=0·31; table 7). For POC and SOC arms, higher viral load testing uptake was seen with the same-site models than with different-site models (all within-category p≤0·0001; table 6; appendix pp 13, 52). There was no evidence of small study effects (publication bias) for any outcome (appendix p 60).

**Table 6:** Pooled estimates for percentage uptake of HCV RNA test and of treatment with POC assay group and laboratory-based SOC assay group, stratified by model of care

		**RNA tested**			**Treated**		
		Arms	Participants	Estimate (95% CI)	Arms	Participants	Estimate (95% CI)
**Overall**							
Onsite POC assay		22	8729	95% (89–99)	34	23 705	77% (72–83)
	Same site, same visit	4	302	100% (99–100)	5	197	97% (92–100)
	Same site, different visit	12	5851	97% (93–99)	20	20 154	74% (66–81)
	Different site, different visit	6	2576	82% (68–92)	9	3354	74% (64–82)
	Within-category p value	..	..	<0·0001	..	..	<0·0001
Mobile POC assay		6	820	84% (43–100)	5	231	81% (60–97)
	Same site, same visit	1	15	80% (52–96)	0	0	NA
	Same site, different visit	2	118	95% (90–99)	2	36	100% (99–100)
	Different site, same visit	1	197	100% (98–100)	1	71	62% (50–73)
	Different site, different visit	2	490	55% (51–60)	2	124	66% (58–74)
	Within-category p value	..	..	<0·0001	..	..	<0·0001
Laboratory-based POC assay		4	6598	92% (68–100)	5	4758	89% (66–100)
	Same site, different visit	2	5208	99% (99–99)	4	4218	85% (65–98)
	Different site, different visit	2	1390	79% (77–81)	1	540	98% (97–99)
	Within-category p value	..	..	<0·0001	..	..	0·030
Laboratory-based SOC comparator assay		5	3526	82% (53–99)	12	5820	53% (31–75)
	Same site, different visit	5	3441	90% (62–100)	8	4931	41% (12–73)
	Different site, different visit	1	85	27% (18–38)	4	889	77% (62–89)
	Within-category p value	..	..	0·0001	..	..	0·049
**People who inject drugs, were homeless, or both**							
Onsite POC assay		12	6154	93% (83–99)	19	5373	73% (64–82)
	Same site, same visit	1	139	100% (97–100)	2	103	94% (88–99)
	Same site, different visit	5	3430	99% (93–100)	9	3205	71% (54–85)
	Different site, different visit	6	2576	82% (68–92)	8	2065	70% (58–81)
	Within-category p value	..	..	<0·0001	..	..	0·0003
Mobile POC assay		6	820	84% (43–100)	5	231	81% (60–97)
	Same site, same visit	1	15	80% (52–96)	0	0	NA
	Same site, different visit	2	118	95% (90–99)	2	36	100% (99–100)
	Different site, same visit	1	197	100% (98–100)	1	71	62% (50–73)
	Different site, different visit	2	490	55% (51–60)	2	124	66% (58–74)
	Within-category p value	..	..	<0·0001	..	..	<0·0001
Laboratory-based POC assay		0	0	NA	2	151	99% (97–100)
	Same site, different visit	0	0	NA	2	151	99% (97–100)
	Different site, different visit	0	0	NA	0	0	NA
	Within-category p value	..	..	NA	..	..	NA
Laboratory-based SOC comparator assay		1	85	27% (18–38)	6	1148	59% (25–88)
	Same site, different visit	0	0	NA	2	259	16% (11–20)
	Different site, different visit	1	85	27% (18–38)	4	889	77% (62–89)
	Within-category p value	..	..	NA	..	..	<0·0001
General and mixed*populations							
Onsite POC assay		4	1076	99% (94–100)	7	15 897	83% (71–92)
	Same site, same visit	2	132	100% (99–100)	2	81	98% (92–100)
	Same site, different visit	2	944	100% (99–100)	5	15 816	76% (61–87)
	Different site, different visit	0	0	NA	0	0	NA
	Within-category p value	..	..	0·80	..	..	0·0006
Mobile POC assay		0	0	NA	0	0	NA
	Same site, same visit	0	0	NA	0	0	NA
	Same site, different visit	0	0	NA	0	0	NA
	Different site, same visit	0	0	NA	0	0	NA
	Different site, different visit	0	0	NA	0	0	NA
	Within-category p value	..	..	NA	..	..	NA
Laboratory-based POC assay		3	2340	88% (42–100))	2	1177	87% (85–89)
	Same site, different visit	1	950	100% (100–100)	1	637	71% (67–74)
	Different site, different visit	2	1390	79% (77–81)	1	540	98% (97–99)
	Within-category p value	..	..	<0·0001	..	..	<0·0001
Laboratory-based SOC comparator assay		2	1750	97% (96–98)	3	4487	69% (24–99)
	Same site, different visit	2	1750	97% (96–98)	3	4487	69% (24–99)
	Different site, different visit	0	0	NA	0	0	NA
	Within-category p value	..	..	NA	..	..	NA
**People incarcerated in prison**							
Onsite POC assay		3	174	92% (77–100)	4	126	89% (67–100)
	Same site, same visit	1	31	100% (89–100)	1	13	100% (75–100)
	Same site, different visit	2	143	85% (78–90)	3	113	84% (57–99)
	Different site, different visit	0	0	NA	0	0	NA
	Within-category p value	..	..	0·0011	..	..	0·14
Mobile POC assay		0	0	NA	0	0	NA
	Same site, same visit	0	0	NA	0	0	NA
	Same site, different visit	0	0	NA	0	0	NA
	Different site, same visit	0	0	NA	0	0	NA
	Different site, different visit	0	0	NA	0	0	NA
	Within-category p value	..	..	NA	..	..	NA
Laboratory-based POC assay		0	0	NA	0	0	NA
	Same site, different visit	0	0	NA	0	0	NA
	Different site, different visit	0	0	NA	0	0	NA
	Within-category p value	..	..	NA	..	..	NA
Laboratory-based SOC comparator assay		3	1691	81% (44–100)	3	185	20% (14–26)
	Same site, different visit	3	1691	81% (44–100)	3	185	20% (14–26)
	Different site, different visit	0	0	NA	0	0	NA
	Within-category p value	..	..	NA	..	..	NA
**People living with HIV**							
Onsite POC assay		3	1334	96% (85–100)	4	2309	75% (50–93)
	Same site, same visit	0	0	NA	0	0	NA
	Same site, different visit	3	1334	96% (85–100)	3	1020	68% (39–92)
	Different site, different visit	0	0	NA	1	1289	91% (89–92)
	Within-category p value	..	..	NA	..	..	0·063
Mobile POC assay		0	0	NA	0	0	NA
	Same site, same visit	0	0	NA	0	0	NA
	Same site, different visit	0	0	NA	0	0	NA
	Different site, same visit	0	0	NA	0	0	NA
	Different site, different visit	0	0	NA	0	0	NA
	Within-category p value	..	..	NA	..	..	NA
Laboratory-based POC assay		1	4258	99% (99–99)	1	3430	53% (52–55)
	Same site, different visit	0	0	NA	1	3430	53% (52–55)
	Different site, different visit	1	4258	99% (99–99)	0	0	NA
	Within-category p value	..	..	NA	..	..	NA
Laboratory-based SOC comparator assay		0	0	NA	0	0	NA
	Same site, different visit	0	0	NA	0	0	NA
	Different site, different visit	0	0	NA	0	0	NA
	Within-category p value	..	..	NA	..	..	NA

Onsite POC refers to HCV RNA assays done onsite; mobile POC refers to assays done in mobile units; laboratory-based POC refers to assays done in one centralised laboratory using a POC device with samples taken at different clinical sites; and SOC refers to centralised, laboratory-based, high-throughput assays. Models of care: same site, same visit refers to testing and treatment initiation at the same site and on the same visit; same site, different visit refers to testing and treatment initiation at the same site but treatment initiation on a different visit; different site, same visit refers to testing at one site with referral to another site for treatment initiation on the same visit; and different site, different visit refers to testing and treatment initiation at different sites and on different visits. HCV=hepatitis C virus. NA=not available. POC=point of care. SOC=standard of care.

* General populations that also contain high-risk groups.

**Table 7:** p values for differences in pooled estimates for percentage uptake of HCV RNA testing and treatment

	**Overall**		**People who inject drugs, were homeless, or both**		General and mixed*populations		**People incarcerated in prison**		**People living with HIV**	
	RNA tested	Treated	RNA tested	Treated	RNA tested	Treated	RNA tested	Treated	RNA tested	Treated
p value for onsite and mobile POC assay subgroups *vs* SOC assay group	0·30	0·029	<0·0001	0·36	0·34	0·53	0·48	<0·0001	NA	NA
p value for onsite, mobile, and laboratory-based POC assay subgroups *vs* SOC assay group	0·31	0·019	<0·0001	0·27	0·81	0·48	0·48	<0·0001	NA	NA
p value for onsite and mobile POC assay subgroups *vs* laboratory-based POC assay subgroup	0·93	0·29	NA	<0·0001	0·37	0·38	NA	NA	0·38	0·089
p value for group laboratory-based POC assay subgroup *vs* SOC assay group	0·48	0·025	NA	0·0010	0·51	0·36	NA	NA	NA	NA

Onsite POC refers to HCV RNA assays done onsite; mobile POC refers to assays done in mobile units; laboratory-based POC refers to assays done in one centralised laboratory using a POC device with samples taken at different clinical sites; and SOC refers to centralised, laboratory-based, high-throughput assays. HCV=hepatitis C virus. NA=not available. POC=point of care. SOC=standard of care.

* General populations that also contain high-risk groups.

Treatment uptake was higher with use of POC assays than with use of SOC assays: 77% (95% CI 72–83; 34 arms) for onsite POC assays, 81% (60–97; five arms) for mobile POC assays, and 89% (66–100; five arms) for laboratory-based POC assays versus 53% (31–75; 12 arms) when using SOC assays (p=0·019; Table 6, Table 7). Treatment uptake was higher in the same-site models than in the different-site models in most comparisons (table 6; appendix p 55). An additional analysis by country income status (high, middle, or low income) for HCV viral load testing uptake and treatment uptake showed no consistent differences, and there was a high degree of heterogeneity within income categories (appendix p 59).

Table 6 and the appendix (pp 43–44) show the pooled estimates for viral load testing and treatment uptake for POC assays versus SOC assays across different population subgroups. However, few comparisons had large numbers of patients and comparable model-of-care arms to allow systematic comparisons. Overall, there was some evidence of higher viral load testing uptake among people who inject drugs, were homeless, or both in the 12 onsite POC arms (93% [95% CI 83–99]) and the six mobile POC arms (84% [43–100]) than in the single SOC assay arm (27% [18–38]; between-group p<0·0001; table 6). There was also some evidence of increased treatment uptake among people incarcerated in prisons for the onsite POC same site and visit model (100% [95% CI 89–100]; one arm) versus the model using onsite POC assays with testing and treatment at the same site but on different visits (85% [78–90]; two arms; p=0·0011). Testing and treatment uptake was higher with same-site care models than with different-site models in people living with HIV (table 6).

**Figure 2:**
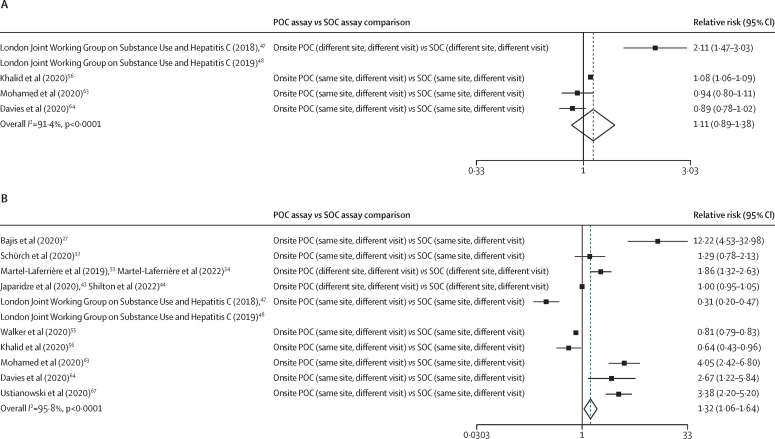
Meta-analysis of within-study comparisons of POC versus SOC HCV RNA assay groups for the relative risks of RNA testing uptake (A) and treatment uptake (B) Weights are from the random-effects analysis. HCV=hepatitis C virus. POC=point of care. SOC=standard of care.

11 studies had at least one POC arm and a SOC arm, enabling direct within-study comparisons for uptake of testing or treatment (figure 2)*.* There was increased treatment uptake (pooled relative risk 1·32 [95% CI 1·06−1·64]) in ten studies with POC versus SOC assays, but no evidence of an increase in viral load testing uptake with POC versus SOC assays (1·11 [0·89–1·38]) in four studies. In the analysis stratified by population group (appendix p 49), there was only one study each among people who inject drugs, were homeless, or both and the general and mixed populations, and both showed higher viral load testing uptake for the POC groups (relative risk 2·11 [95% CI 1·47–3·03] for people who inject drugs, were homeless, or both and 1·08 [1·06–1·09] for the general and mixed populations) versus SOC groups. There was no evidence for increased treatment uptake with POC groups in the five studies among people who inject drugs, were homeless, or both (relative risk 1·38 [95% CI 0·70–2·71]), and preliminary evidence of decreased treatment uptake in the two general and mixed population studies (0·79 [0·67–0·92]), but these studies were still ongoing, and the SOC comparator was based on historical data. Among people incarcerated in prison, there was no evidence of increased testing uptake with use of POC assays compared with use of SOC assays in two studies (relative risk 0·91 [95% CI 0·82–1·01]), but there was higher treatment uptake than with SOC assays in three studies (3·47 [2·56–4·71]).

Of the 45 studies, the risk of bias was rated as high in 16 (36%), moderate in 18 (40%), and low in 11 (24%; appendix p 13). Overall, the quality of evidence was graded as being low due to the absence of randomised controlled trials, with only observational studies being available (appendix pp 60–63).

## Discussion

This is the first global systematic review and meta-analysis to examine the effectiveness of POC HCV viral load testing as a diagnostic alternative to centralised, laboratory-based viral load assays to confirm presence of HCV viraemic infection and, therefore, need for treatment. The analysis was based on 45 mostly single-arm observational studies (with 64 arms in total) that had used POC viral load and had data on outcomes across the cascade of care, especially HCV viral load testing and treatment uptake, and turnaround times. 11 studies had within-study POC versus SOC comparator arms, providing a more rigorous evidence base.

There were several key findings. First, compared with SOC viral load testing, the pooled median turnaround times between HCV antibody testing and treatment initiation was reduced with onsite POC assays (19 days) versus laboratory-based POC assays (64 days) or laboratory-based SOC assays (67 days), mainly due to reduced time from viral load testing to treatment initiation. Second, there was an overall increase in treatment uptake with onsite (77%) or mobile POC assays (81%) compared with SOC assays (53%). Third, increased HCV viral load testing and treatment uptake with POC viral load assays was greatest among people who inject drugs, were homeless, or both for viral load testing uptake, and among people incarcerated in prison for treatment uptake. Fourth, direct within-study comparisons of groups receiving POC and SOC testing confirmed the shorter turnaround times in the POC groups in seven studies and improved treatment uptake in ten studies (relative risk 1·32 [95% CI 1·06–1·64]) and some evidence to suggest increased viral load testing uptake among those receiving POC testing (1·11 [0·89–1·38]) in four studies. Finally, the impact of POC viral load was greatest when positioned in a simplified care model in which testing and treatment were done at the same site, and, where possible, on the same day.

There were several key strengths of this review. First, we adopted a rigorous stratification of studies by different models of care so that evaluation of impact of POC was analysed in the context of the level of simplified care model in place—ie, whether testing and treatment were delivered at the same or different sites and on the same or different days. This is crucial, as previous work has shown that full decentralisation of HCV testing and treatment at the same site compared with partial or no decentralisation is associated with increased uptake of viral load testing and treatment.^3^ Second, direct within-study comparisons of people receiving POC and SOC testing was done in 11 studies, and pooled analysis in this subgroup of studies confirmed our overall findings of increased treatment uptake and reduced turnaround times. Third, almost half of the studies were from LMICs, and they accounted for all the studies among the general population and people living with HIV.

The key limitation to this review was the absence of randomised controlled trials that directly compared use of POC assays and laboratory-based SOC assays, so our analysis was largely based on single-arm observational studies of POC RNA assays, and the overall risk-of-bias rating was high. Although 12 studies had a comparator SOC group, these studies often relied on historical rather than concurrent data. There were few studies of people incarcerated in prison, and all studies in this population were from high-income countries, whereas the four studies in people living with HIV were all from LMICs. Although the majority of studies had data on the two key outcomes of uptake of viral load testing and treatment, less than 20% had data on turnaround times. As with our previous review evaluating effectiveness of decentralisation, integration, and task sharing,^3^ there was considerable heterogeneity in the models of care and other interventions adopted across the different studies, and the reporting of this information was not consistent and often missing. Although the majority reported using rapid diagnostic HCV antibody tests, there was variable adoption of the simpler fingerstick^19^, ^21^, ^23^, ^25^, ^27^, ^30^, ^31^, ^59^, ^60^, ^61^, ^64^ rather than venous blood sampling for POC viral load testing, reflex viral load testing,^42^, ^44^ task sharing to primary care physicians and nurses,^19^, ^20^, ^23^, ^24^, ^26^, ^27^, ^28^, ^34^, ^35^, ^38^, ^55^, ^62^ and provision of cash incentives, food vouchers, and travel reimbursement.^19^, ^24^, ^27^, ^28^, ^47^, ^48^, ^52^ Importantly, not all studies offered services and treatment free of charge, and the requirement of out-of-pocket expenditure in some would be a major barrier to viral load testing and treatment uptake.^53^ It is noteworthy that only a few studies specifically stated the objective of same-day testing and treatment.^29^, ^49^, ^61^ Several of the earlier studies using POC assays still had restrictive treatment criteria, required genotyping,^21^, ^23^, ^34^, ^55^, ^63^, ^69^ a series of visits before treatment initiation, or treatment visits scheduled from 2 weeks to 12 weeks from positive viral load test or to coincide with twice monthly clinics,^19^, ^20^, ^24^, ^27^, ^58^, ^63^ and, therefore, would not reflect the full impact of the faster turnaround time with the onsite POC viral load assay. With progressive simplification of the care pathway, including adoption of a treat-all approach, use of pan-genotypic regimens (and so dispensing with the need for genotyping), task sharing to non-specialist doctors and nurses, and reduced visits, the uptake and turnaround times with POC assays are now much shorter.

POC molecular viral load assays are widely used and already recommended by WHO for diagnosis of other infectious diseases, such as tuberculosis^9^ and HIV^7^ for early infant diagnosis^8^, ^72^ and routine viral load monitoring for people living with HIV on antiretroviral therapy.^7^ The recent updated HIV guidance was based on high-quality data from randomised controlled trials and, consistent with our own findings for HCV, showed that HIV POC molecular testing was associated with faster result delivery time and antiretroviral therapy initiation in HIV-positive infants.^8^, ^72^ Similarly, HIV viral load monitoring using a POC RNA assay resulted in faster return of results to patients (0 *vs* 28 days, hazard ratio [HR] 17·7 [95% CI 13·0–24·2]) and clinicians (11·7 [8·9−15·3]), and time to clinical action for elevated viral load (0 *vs* 76 days, 10·9 [2·1–57·5]) than laboratory-based SOC comparators.^17^, ^72^ Many of these same principles and benefits in use of POC platforms with HIV and tuberculosis apply to HCV, despite differences in the care models, and can be regarded as indirect evidence to support its use. The COVID-19 pandemic has also prompted considerable expansion in molecular diagnostics capacity in many LMICs, including use of POC platforms for SARS-CoV-2 testing. This provides a further opportunity to leverage this capacity for multidisease testing.

This systematic review has several major policy and clinical management implications for scale-up of testing and treatment needed to achieve global HCV elimination targets. First, this evidence base alongside that of HIV POC viral load testing has informed new 2022 WHO recommendations for use of POC HCV viral load as an alternative approach to laboratory-based platforms both for diagnosis of HCV viraemic infection and as a test of cure.^73^ This will be particularly relevant for promoting linkage to care at decentralised, co-located HCV testing and treatment sites that might include harm-reduction services, primary or secondary care level clinics, prisons, and HIV clinics.

Second, other recent work has shown that one of the most important interventions to promote access and improve uptake of HCV testing and treatment is delivery of fully decentralised testing and treatment at the same site, a so-called one-stop shop, ideally alongside other services.^3^ Six studies in our review had a same-day test and treat model. Our review reinforces this message, with the demonstration that the impact of POC viral load was greatest when delivered as part of a one-stop shop service. It is recognised that effective innovations such as use of POC platforms might not achieve expected outcomes if other barriers in the care pathway are not addressed, such as requiring patients to attend multiple appointments before treatment initiation, the treatment site being far from where they live, or costs of viral load assays being high.

Third, regarding the optimal implementation strategy for POC HCV viral load testing, this review provides strong evidence that the best outcomes are seen when these assays are used closer to the patients as true POC assays, particularly when placed onsite or in mobile units, rather than in hub laboratories. The optimal settings for the provision of POC HCV viral load are likely to be where there are populations at high risk of attrition, such as homeless populations and people who inject drugs, or in hard-to-reach remote settings. Some studies included within this review incorporated innovative strategies for delivering POC viral load to hard-to-reach populations through mobile units^23^, ^26^, ^29^, ^36^, ^49^ and special campaigns that offered same-day testing and treatment.^61^ For people living in prison, fast-tracking diagnosis and treatment initiation upon entry to prison increases their chance of completing their treatment and being cured before release.^63^, ^64^, ^65^, ^67^ The choice of where to optimally deploy POC viral load versus laboratory assays will depend on various factors, including assay characteristics, cost, and characteristics of the testing site (including site location and number of patients treated there). The introduction of multidisease POC testing platforms brings new opportunities for integration of HCV viral load testing and might be able to provide substantial system efficiencies and cost savings.^74^ Similarly, there are examples of where a centralised, laboratory-based system has been highly effective when supported by efficient sample transport and result delivery networks.^43^

Finally, the review highlights the need for more rigorous comparative studies of the use of POC molecular platforms for HCV viral load for diagnosis and treatment monitoring. This should be considered alongside other interventions to promote the uptake of viral hepatitis testing and linkage to care and monitoring, such as use of peer workers to promote linkage, dried blood spots, and reflex viral load testing, as well as studies in other vulnerable groups, such as people who are homeless but do not inject drugs. Future studies should provide a full description of the testing and treatment care pathway and service delivery models and all relevant interventions. This might include which clinical staff are providing testing and treatment, and details of other interventions in addition to use of POC assays (and whether fingerstick or venepuncture sampling) to promote access and uptake. Evaluation should capture effectiveness of interventions across the entire continuum of care including uptake of testing, linkage to care, and treatment initiation, as well as turnaround times.

## Data sharing

Aggregate, rather than individual-level, data were included in these analyses from published manuscripts and conference publications, which are publicly available. For aggregate data taken from the grey literature that are not publicly available, please contact the corresponding author.

## Declaration of interests

We declare no competing interests.
