# Supplementary material for: Impact of hepatitis C virus point-of-care RNA viral load testing compared with laboratory-based testing on uptake of RNA testing and treatment, and turnaround times: a systematic review and meta-analysis
Source: Lancet Gastroenterol Hepatol. 2023 Jan 24;8(3):253–70. doi: 10.1016/S2468-1253(22)00346-6 (PMC11810864; doi:10.1016/S2468-1253(22)00346-6)
Supplement: Supplementary appendix [file mmc1.pdf]

THE LANCET
Gastroenterology & Hepatology

## Supplementary appendix

This appendix formed part of the original submission and has been peer reviewed. We post it as supplied by the authors.

Supplement to: Trickey A, Fajardo E, Alemu D, Artenie AA, Easterbrook P. Impact of hepatitis C virus point-of-care RNA viral load testing compared with laboratory-based testing on uptake of RNA testing and treatment, and turnaround times: a systematic review and meta-analysis. *Lancet Gastroenterol Hepatol* 2023; published online Jan 24. https://doi.org/10.1016/S2468-1253(22)00346-6.

**Effect of hepatitis C virus (HCV) point-of-care RNA viral load testing compared with laboratory-based testing on uptake of RNA testing and treatment, and turnaround times: a systematic review and meta-analysis**

**SUPPLEMENTARY MATERIALS**

2023/01/19

Adam Trickey, Emmanuel Fajardo, Daniel Alemu, Andreea Adelina Artenie, Philippa Easterbrook

**TABLE OF CONTENTS**

Page 3 - Why it is important to do this review

Page 4 - Supplementary table 1: Outline of PICO (Population, intervention, comparator, outcomes) question.

Page 5 - Supplementary table 2: Point of care hepatitis C virus ribonucleic acid testing platforms

Page 6 - Supplementary table 3: Search strategy

Page 7 - Methods: Search strategy and terms

Page 8 – Methods: Criteria for selection of studies for inclusion

Page 9 - Supplementary table 4: Categorising the turnaround time information provided by the studies

Page 11 - Quantitative time data (days) for steps not covered in the manuscript

Page 12 - Methods: Risk of bias assessment

Page 13 - Supplementary table 5: Risk of bias score for each study

Page 14 - Supplementary figure 1: Geographic distribution of countries from 45 studies included within the review

Page 15 - Supplementary table 6: Detailed description of each study

Page 30 - Supplementary table 7: Summary of study characteristics and outcomes across HCV cascade of care for the 64 arms from the 45 studies with at least one PoC assay arm.

Page 34 - Supplementary table 8: Median time in days between key steps in the HCV cascade of care for each of the 27 studies with time data available, stratified by model of care arm.

Page 36 - Outcome 1: Turn-around times

Page 38 - Direct within-study comparisons of median turnaround times

Page 39 - Supplementary table 9: Weighted within-study differences in the median days between HCV cascade of care steps, stratified by population group, for 7 studies with PoC and lab-based RNA assay comparator arms

Page 40 - Outcome 2a: HCV RNA testing uptake (all population groups combined)

Page 41 - HCV RNA testing and treatment uptake among population subgroups (outcomes 2a and 2b)

Page 43 - Supplementary table 10: Pooled estimates for percentages uptake of RNA testing and treatment for use of PoC RNA assays compared to lab-based RNA assays, stratified by model of care categorisation.

Page 45 - Direct within-study comparisons of cascade of care proportions

Page 47 - Supplementary table 11: Percentage of patients achieving each step in the cascade for PoC vs non-PoC RNA assay arms

Page 49 - Supplementary figure 2a-b: Meta-analysis of within study comparisons of PoC vs lab assay arms for the relative risks of a) RNA testing uptake and b) treatment uptake, stratified by population type.

Page 50 - Supplementary figure 3a-h: Forest plots of cascade of care outcomes

- Page 50 - a) HCV antibody tested
- Page 51 - b) HCV antibody positive, of those tested
- Page 52 - c) HCV RNA tested, of those anti-HCV positive
- Page 53 - d) HCV RNA positive, of those tested
- Page 54 - e) Had post-RNA assessment, of those RNA positive
- Page 55 - f) Started treatment, of those RNA positive
- Page 56 - g) SVR12 assessed, of those that started treatment
- Page 57 - h) SVR12 achieved of those with SVR12 assessed

Page 58 - Outcomes across other cascade of care steps

Page 59 - Comparing outcomes across country income groups

Page 60 - Supplementary table 12: Test for publication bias* for each cascade outcome proportion using a regression-based Egger test (Random-effects model)

Page 61 - Grading the quality of evidence

Page 62 - Supplementary table 13: Grading the quality of evidence for time to event data, using the indirect pooled comparisons of PoC RNA assays with lab-based RNA assays.

Page 63 - Supplementary table 14: Grading the quality of evidence for the proportion of people with uptake of RNA testing and HCV treatment, comparing PoC RNA assays with lab-based RNA assays.

Page 64 – References

## *WHY IT IS IMPORTANT TO DO THIS REVIEW*

Understanding the impact of use of a point of care (POC) nucleic acid testing (NAT) compared to conventional laboratory-based NAT testing in promoting uptake of diagnosis of hepatitis C virus (HCV) viraemic infection will help inform updated World Health Organization (WHO) testing guidelines.

The primary and secondary research questions and related PICO (population, intervention, comparator, and outcome) question are given below.

**Primary research question:**

- Does use of point of care HCV viral load testing increase uptake of HCV viral load testing and HCV treatment and reduce time to treatment initiation in HCV antibody positive persons compared to standard-of-care laboratory-based NAT testing approaches?

**Secondary research question:**

- Is there a differential effect of point of care HCV viral load testing when used in specific models of care (fully decentralized testing and treatment services at same site compared to partially decentralized services with testing at one site and referral for treatment) and in specific populations, e.g. stigmatized or hard to reach populations with poor access to care and higher rates of loss to follow-up, such as people who inject drugs (PWID), homeless and migrant populations, or persons living with HIV (PLHIV), compared to general population/primary care settings?

**PICO question:** Does point of care HCV viral load testing increase uptake of HCV viral load testing and HCV treatment and reduce time to HCV viral load test results and treatment initiation in HCV antibody positive persons compared to laboratory-based standard-of-care approaches?

**Study design:** All study designs were included.

Further information on the study can be found on the PROSPERO protocol: https://www.crd.york.ac.uk/PROSPERO/display_record.php?RecordID=218239

Preferred Reporting Items for Systematic Reviews and Meta-Analysis (PRISMA) was used for the study design and reporting[1].

**Supplementary table 1:** Outline of PICO question.

| Population | Intervention | Comparator | Outcomes |
|---|---|---|---|
| Persons at risk of HCV infection targeted for testing in harm reduction settings, HIV clinics, prisons, primary care, or other community-based settings. All countries/locations eligible.<br><br>Subgroups of interest: PWID, prisoners, PLHIV, men who have sex with men (MSM), people who are homeless, general population in primary care settings.<br><br>Stratified by:<br><br>**Location of PoC test** (on-site and mobile) and Lab-based assay (PoC or high throughput lab-based standard of care)<br><br>**Model of care:** (same-site and same-day for testing and treatment; same-site but different day for testing and treatment; different site but same-day for testing and treatment; different day and different site for testing and treatment). | Use of point of care HCV NAT viral load assay<br><br>- Onsite at same location as testing site<br><br>- In mobile unit<br><br>- Lab-based PoC assay as "hub and spoke" model | Use of laboratory-based NAT HCV viral load standard of care assay | **Outcome 1: Turn-around times (days):**<br>- Overall time from HCV antibody test to treatment start.<br>- HCV antibody test to HCV RNA test.<br>- RNA sample collection to test.<br>- RNA test to results made available.<br>- RNA test to treatment start.<br><br>**Outcome 2: Cascade of care proportions:**<br>- Uptake of HCV viral load test in those anti-HCV positive,<br>- Uptake of treatment in those HCV RNA positive,<br>- Uptake of sustained virologic response (SVR) testing in those completing treatment, and those achieving SVR12 among those tested.<br>Overall and stratified by population, and model of care |

**Supplementary table 2:** Point of care (PoC) hepatitis C virus (HCV) ribonucleic acid (RNA) testing platforms[2]

| **Platform** | **Company** |
|---|---|
| **Developed:** | |
| GeneXpert® System | Cepheid |
| Mini8 Plus Real-Time PCR system | Coyote Bioscience |
| Genedrive® HCV ID kit | Epistem Ltd |
| **In the pipeline:** | |
| Alere™ q | Alere |
| GeneXpert® Omni | Cepheid |
| Truenat™ (previously Truelab™ Real Time [illegible] | Molbio Diagnostics Pvt. Ltd |
| RT CPA HCV Viral Load Test | Ustar Biotechnologies |

In general, these PoC devices can be battery operated and so are not dependent on continuous electricity to run, use reagents that do not require refrigeration and can be stored at ambient temperatures. Testing procedures can use a fingerstick sample or whole blood and the result can be available within 2 hours to inform clinical decision-making.

**Supplementary table 3:** Search strategy and terms

| | Concept | Terms |
|---|---|---|
| 1 | Point of Care | (Point of Care) OR (Point-of-Care) OR (POC) OR (POCT) OR (Rapid) OR (decentrali* test*) OR (community-based test*) OR (outreach test*) OR (same-day diagnosis) OR (GeneXpert) OR (Xpert) OR (Cepheid) OR (Coyote) OR (Mini8) OR (Genedrive) OR (Epistem) OR (Genedrive plc) OR (AlereQ) OR (Alere) OR (Truenat) OR (Truelab) OR (Molbio) OR (Molbio Diagnostics) OR (Ustar) OR (RT CPA) |
| 2 | HCV | (HCV) OR (Hepatitis C Virus) OR (Hepatitis C) OR (Hepatitis C infection) OR (chronic infection) OR (active infection) |
| 3 | Viral load | (Viral load) OR (VL) OR (RNA) OR (PCR) OR (RT-PCR) OR (molecular test*) OR (NAT) OR (NAAT) OR (nucleic acid testing) |
| 4 | Uptake | (Uptake) OR (effect) OR (linkage to care) OR (enrolment) OR (utilization) OR (diagnostic cascade) OR (test and treat) |
| 5 | HCV treatment | (HCV treatment) OR (HCV therapy) OR (DAA) OR (DAA regimen) OR (direct acting antiviral) OR (HCV drugs) OR (antiviral agents) |
| 6 | Time to treatment initiation | (time treatment initiation) OR (treatment initiation) OR (treatment cascade) OR (same-day treatment) OR (care cascade) |
| 7 | Standard of care | (standard of care) OR (laboratory-based testing) OR (centrali* testing) |
| 8 | 1 AND 2 AND 3 AND (4 OR 5 OR 6 OR 7) | |
| 9 | Restricted 01/01/2016 – 23/09/2020 | |

*Search terms (or equivalent):*

((Point of Care) OR (Point-of-Care) OR (POC) OR (POCT) OR (Rapid) OR (decentrali* test*) OR (community-based test*) OR (outreach test*) OR (same-day diagnosis) OR (GeneXpert) OR (Xpert) OR (Cepheid) OR (Coyote) OR (Mini8) OR (Genedrive) OR (Epistem) OR (Genedrive plc) OR (AlereQ) OR (Alere) OR (Truenat) OR (Truelab) OR (Molbio) OR (Molbio Diagnostics) OR (Ustar) OR (RT CPA))

AND

((HCV) OR (Hepatitis C Virus) OR (Hepatitis C) OR (Hepatitis C infection) OR (chronic infection) OR (active infection))

AND

((Viral load) OR (VL) OR (RNA) OR (PCR) OR (RT-PCR) OR (molecular test*) OR (NAT) OR (NAAT) OR (nucleic acid testing))

AND

((Uptake) OR (effect) OR (linkage to care) OR (enrolment) OR (utilization) OR (diagnostic cascade) OR (test and treat) OR (HCV treatment) OR (HCV therapy) OR (DAA) OR (DAA regimen) OR (direct acting antiviral) OR (HCV drugs) OR (antiviral agents) OR (time treatment initiation) OR (treatment initiation) OR (treatment cascade) OR (same-day treatment) OR (care cascade) OR (standard of care) OR (laboratory-based testing) OR (centrali* testing))

AND

("2016/01/01"[Date - Publication] : "2020/09/23"[Date - Publication])

*METHODS: SEARCH STRATEGY*

We carried out a search of PubMed, Embase, and Web of Science for studies that used HCV PoC RNA assays and contained data on HCV cascade of care outcomes and/or turnaround times for steps in the cascade of care. The search was carried out on the 23rd September 2020 on studies in English published from 1st January 2016 (to be conservative as the WHO pre-qualification of the Cepheid GeneXpert was in 2017[3]).

In addition to this main search, which captured abstracts from key relevant conferences, an additional search was performed for relevant conference abstracts from 2016-2020, which were not picked up by the main search. These were the European Association for the Study of the Liver's (EASL) International Liver Conference 2020 (that was too recent to be picked up by the main search), the International Network on Hepatitis in Substance Users (INHSU) symposiums from 2016-2019 (which were not picked up by the main search – the 2020 conference did not take place), and the International Viral Hepatitis Elimination Meeting (IVHEM) 2020 (too recent to be picked up by the main search). Other relevant conferences, such as older International Liver Conferences, and those of the American Association for the Study of Liver and the Asia-Pacific Association for the Study of Liver were picked up by our main search strategy. Finally, we used the WHO partner organisations and contacts database to contact key WHO contacts in relevant organisations, such as the manufacturers of the assays, Médecins Sans Frontieres (MSF), and the Foundation for Innovative New Diagnostics (FIND) for knowledge of other studies (either completed or ongoing) that involved use of HCV PoC RNA assays. The reference lists of all retrieved articles, as well as review articles identified during the initial search, were screened for citations to relevant studies.

For the main search, authors AT and EF conducted the search and independently evaluated the articles (first the titles/abstracts and then the full-texts of those selected from the title/abstract screening) to determine the study eligibility, with PE reviewing the final selection and arbitrating differences between the primary reviews. For the search of the EASL 2020, INHSU 2016-2019, and IVHEM 2020 abstracts, authors AT and DA conducted the search, independently evaluating the articles for study eligibility, with EF arbitrating the differences between the two primary reviewers and making the final selection. For the other studies identified through contacts, AT and EF independently evaluated each proposed study for eligibility, with PE arbitrating the differences and making the final selection. Manuscript references were checked by AT and DA, with EF arbitrating selection differences.

*METHODS: CRITERIA FOR SELECTION OF STUDIES FOR INCLUSION*

Original studies were included if they contained use of HCV RNA assays that could be used as PoC assays (even if used in a laboratory setting) with or without a comparator centralised laboratory-based assay (standard-of-care) and had data on HCV cascade of care outcomes and/or turnaround times between different steps. Cascade of care outcomes were defined as data on at least two adjacent steps in the HCV cascade of care (to be able to calculate a proportion from a numerator and denominator). These steps were the:

- number and proportion tested for HCV antibodies who were offered testing;
- number and proportion HCV antibody positive of those tested;
- number and proportion tested for HCV RNA of those HCV antibody positive;
- number and proportion testing HCV RNA positive of those tested for HCV RNA;
- number and proportion attending post-RNA pre-treatment assessment visits of those testing HCV RNA positive;
- number and proportion initiating treatment who had a post-RNA pre-treatment assessment;
- number and proportion completing treatment of those who initiated treatment;
- number and proportion who had an SVR12 assay of those who completed treatment and were eligible;
- and number and proportion of those achieving SVR12 who had an SVR12 visit test.

Additionally, studies would also be included if they contained data on times between HCV cascade of care stages, including intermediate steps such as the time for receipt of results to patients. Reviews were excluded. All population groups were included. All study designs were eligible if the separate data were available for patients undergoing testing with the PoC RNA assays and from other assays. Studies with any type of HCV treatment (DAAs, interferon-based, or a combination) were eligible.

If necessary, data obtained from abstracts or grey literature were returned to authors for verification or provision of additional information. Studies with a sample size of ten or less for the largest denominator were excluded.

To make the analyses of cascade of care proportion of those initiating treatment more homogenous across studies, we instead used the number of HCV RNA positive individuals as the denominator, rather than the post-RNA treatment assessment visits, which were not universally performed. However, this information is still presented for information in supplementary table 7.

**Supplementary table 4:** Categorising the turnaround time information provided by the studies

| |
|---|
| **Entry to Ab testing (for prisons)** |
| Arrival to screening test performed |
| Patient entry to test |
| **Ab test to RNA test** |
| Days between RDT and qual PCR: |
| Days between RDT and Xpert: |
| RDT collection - RNA test |
| HCV screening and sample collection for confirmation |
| Time from RDT to RNA test |
| Screen date to sample collection for VL |
| Median time from HCV antibody positive diagnosis to obtaining HCV VL results |
| **RNA sample collected to RNA tested** |
| Sample collection for VL to viremia test date |
| VL sample collection and testing completion |
| Time between sample collection and testing on Xpert |
| **RNA test returned to patient** |
| RNA Results available on same day |
| Days between RNA test and results available |
| Provisional Xpert FS assay results were delivered to participants |
| Test performed to RNA result |
| VL sample testing completion to patient notification |
| Time from RNA test and result available: |
| Median time from VL results being made available |
| Median turnaround time (sample collection to receipt of lab results by workers at clinic) for Xpert |
| Median turnaround time (sample collection to receipt of lab results by workers at clinic) for Roche |
| **RNA test to treatment start** |
| Viremia testing to treatment initiation |
| Time from final RNA test run date to DAA initiation date |
| The response time between RNA test result and treatment start |
| days between RNA and treatment start |
| Days between Xpert and treatment start |
| Delay between initial visit (RNA) and treatment initiation |
| days from RNA to treatment |
| RNA test to tx start |
| Median time to treatment initiation from screening (RNA): |
| VL result to patient and patient started treatment |
| The median time from RNA tests to treatment |
| Median time from HCV diagnosis to treatment initiation |
| Median days from RNA tests to treatment start |
| **Ab test to treatment start** |
| Total time |
| RDT collection - tx start |
| Total time (screening to treatment start) |
| The median time from Anti-HCV tests to treatment |

| | |
|---|---|
| **Ab testing to clinical assessment** |
| Ab test result to clinical assessment |
| **RNA testing to clinical assessment** |
| RNA test result to clinical assessment |
| **Clinical assessment to treatment** |
| Clinical assessment to treatment commenced |
| **Other times captured by the studies, that were not classified into the categories above** |
| Test performed to Ab result |
| Test performed to genotype result |
| Time from lab receipt to RNA result dispatch |
| Median turnaround time (sample collection to receipt of lab results by workers at clinic) for genotyping |
| Days between qual PCR and quant PCR |
| Days between Quant PCR and treatment start |
| Median time between 1st (PoC tests) and 2nd visit (follow-up) |
| Days between RNA results available and referral for assessment |
| Median time from patient arrival to departure (This refers to receiving tests, assessment and generally results, and also treatment if positive, but is not consistent in what it's measuring as it differs for each patient) |
| VL testing to database entry |

**Quantitative time data (days) for steps not covered in the main document**

Median time in days between arrival and antibody testing for each study arm

| | |
|---|---|
| Shiha (C1) | 0 |
| Shiha (C2) | 0 |
| Mohamed (C2) | 6 |
| Mohamed (C1) | 2 |
| Valencia | 0 |
| Davies (C1) | 1 |
| Llerena | 0 |
| Ustianowski (C1) | 1 |
| Sonderup | 0 |

Median time in days between Ab test and assessment for each study arm

| | |
|---|---|
| Shiha (C1) | 0 |
| Shiha (C2) | 0 |
| Mohamed (C1) | 3 |
| Valencia | 0 |
| Llerena | 0 |
| Sonderup | 0 |

Median time in days between RNA test and assessment for each study arm

| | |
|---|---|
| Shiha (C1) | 0 |
| Shiha (C2) | 0 |
| Mohamed (C2) | 14 |
| Valencia | 0 |
| Stone | 0 |
| Llerena | 0 |
| Sonderup | 0 |

Median time in days between assessment and treatment start for each study arm

| | |
|---|---|
| Shiha (C1) | 0 |
| Shiha (C2) | 0 |
| Mohamed (C2) | 36 |
| Mohamed (C1) | 1 |
| Valencia | 0 |
| Stone | 0 |
| Llerena | 0 |
| Sonderup | 0 |

*METHODS: RISK OF BIAS*

Authors EF and AT both assessed the risk of bias for each study using a previously published and modified risk of bias tool used for observational studies that report binary outcomes based on tools by Hoy et al and the ROBINS-I tool[4-6]. Due to a lack of any RCTs, the question “Was some form of random selection used to select the sample?” was modified to “Was prospective selection used to select the sample?”. Where there was a disagreement between the two overall risk of bias assessments from the initial assessors, a third author, AAA, arbitrated.

**Domain of bias:**

Selection bias:

1. Was the study’s target population pre-specified and appropriately chosen to answer the study question(s)?
2. Was the sampling frame a true or close representation of the target population?
3. Was prospective selection used to select the sample?

Bias due to missing outcome data:

4. Was the proportion of missing outcome data minimal and were there none/minimal differences between those with and without missing outcome data?

Bias in measurement of the outcome:

5. Were data collected directly from the subjects (as opposed to a proxy?)
6. Was an acceptable case definition used in the study?
7. Was the study instrument that measured the parameter of interest (e.g. proportion of results obtained on the same day) shown to have reliability and validity?
8. Was the same mode of data collection used for all subjects?
9. Was the length of the shortest measurement period appropriate for the parameter of interest?

Bias in analysis:

10. Were the numerator(s) and denominator(s) for the parameter of interest appropriate?

**Overall judgement of risk of bias:**

Low risk: all domains indicated as low risk

Some/medium risk: 1 or no domains have signalling question indicating high risk; OR, at least one signalling question without information.

High risk: 2 or more domains have signalling questions indicating high risk

**Supplementary table 5:** Risk of bias score for each study

| Study | 1. Target population | 2. Sampling frame | 3. Prospective selection | 4. Missing outcome | 5. Data from subjects | 6. Acceptable case definition | 7. Study instrument | 8. Same mode of collection | 9. Appropriate parameter | 10. Num/den appropriate | Overall bias |
|---|---|---|---|---|---|---|---|---|---|---|---|
| Bajis | Y | Y | Y | N | Y | Y | Y | Y | Y | Y | Medium |
| Mohamed | Y | Y | Y | Y | Y | Y | Y | Y | Y | Y | Low |
| Davies | Y | Y | Y | Y | Y | Y | Y | Y | N | Y | Medium |
| Llerena | Y | Y | Y | Y | Y | Y | Y | Y | Y | Y | Low |
| Agwuocha | Y | Y | Y | N | Y | Y | Y | Y | Y | N | High |
| MSF Uttar Pradesh | Y | Y | Y | N | Y | Y | Y | Y | Y | Y | Medium |
| MSF Phnom Penh | Y | Y | Y | N | Y | Y | Y | Y | Y | Y | Medium |
| MSF Karachi | Y | Y | N | N | Y | Y | Y | Y | N | Y | High |
| Qureshi | Y | Y | Y | N | Y | Y | Y | Y | N | Y | High |
| Qureshi | Y | Y | N | Y | Y | Y | Y | Y | N | Y | High |
| Hamid | Y | Y | Y | Y | Y | Y | Y | Y | N | Y | Medium |
| Shiha | Y | Y | Y | Y | Y | N | Y | Y | Y | Y | Medium |
| Chevaliez | Y | Y | Y | Y | Y | Y | Y | Y | N | Y | Medium |
| Lens | Y | Y | Y | Y | Y | Y | Y | Y | Y | Y | Low |
| Lazarus | Y | Y | Y | Y | Y | Y | Y | Y | N | Y | Medium |
| Rogers | Y | Y | N | N | Y | Y | Y | Y | N | Y | High |
| Antonini | Y | Y | Y | N | Y | Y | Y | Y | N | Y | High |
| Remy | Y | Y | Y | Y | Y | Y | Y | Y | Y | Y | Low |
| Bajis | Y | Y | Y | N | Y | Y | Y | Y | Y | Y | Medium |
| Williams | Y | Y | Y | N | Y | Y | Y | Y | Y | Y | Medium |
| Valencia | Y | Y | Y | Y | Y | Y | Y | Y | Y | Y | Low |
| Saludes | Y | Y | N | Y | Y | Y | Y | Y | Y | Y | Medium |
| Schurch | Y | Y | N | N | Y | Y | Y | Y | N | Y | High |
| Martel-Laferriere | Y | Y | Y | Y | Y | Y | Y | Y | Y | Y | Low |
| Feld | Y | Y | Y | N | Y | Y | Y | Y | N | Y | High |
| Thingnes | Y | Y | Y | Y | Y | Y | Y | Y | Y | Y | Low |
| Stone | Y | N | Y | Y | Y | Y | Y | Y | Y | Y | Medium |
| MSF Mafalala | Y | N | N | N | Y | Y | Y | Y | Y | Y | High |
| Butsashvili | Y | Y | N | N | Y | Y | Y | Y | Y | N | High |
| Aung | Y | Y | Y | N | Y | Y | Y | Y | Y | Y | Medium |
| HEADSTART Manipur | Y | Y | Y | Y | Y | Y | Y | Y | Y | Y | Low |
| HEADSTART Georgia | Y | Y | Y | Y | Y | Y | Y | Y | Y | Y | Low |
| HEADSTART Myanmar | Y | Y | Y | Y | Y | Y | Y | Y | Y | Y | Low |
| HEADSTART Punjab | Y | Y | Y | N | Y | Y | Y | Y | Y | Y | Medium |
| MSF Maputo | Y | Y | N | N | Y | Y | Y | Y | N | Y | High |
| MSF Mykolaiv | Y | Y | N | N | Y | Y | Y | Y | N | Y | High |
| MSF Dawei | Y | Y | N | Y | Y | Y | Y | Y | N | Y | High |
| LJWG Homeless | Y | Y | Y | N | Y | Y | Y | Y | N | Y | High |
| LJWG NSPs | Y | Y | Y | N | Y | N | Y | Y | N | Y | High |
| Ustianowski | Y | Y | N | N | Y | Y | Y | Y | Y | Y | High |
| SOS hepatites | Y | N | N | Y | Y | Y | Y | Y | Y | Y | Medium |
| Morris | Y | Y | Y | Y | Y | Y | Y | Y | Y | Y | Low |
| Wansom | Y | Y | Y | Y | Y | Y | Y | Y | N | Y | Medium |
| Sonderup | Y | Y | Y | Y | Y | Y | Y | Y | N | Y | Medium |
| Zhang (MSF) | Y | Y | Y | Y | Y | Y | Y | Y | N | Y | Medium |

**Supplementary figure 1:** Geographic distribution of countries from 45 studies included within the review (shaded in red).

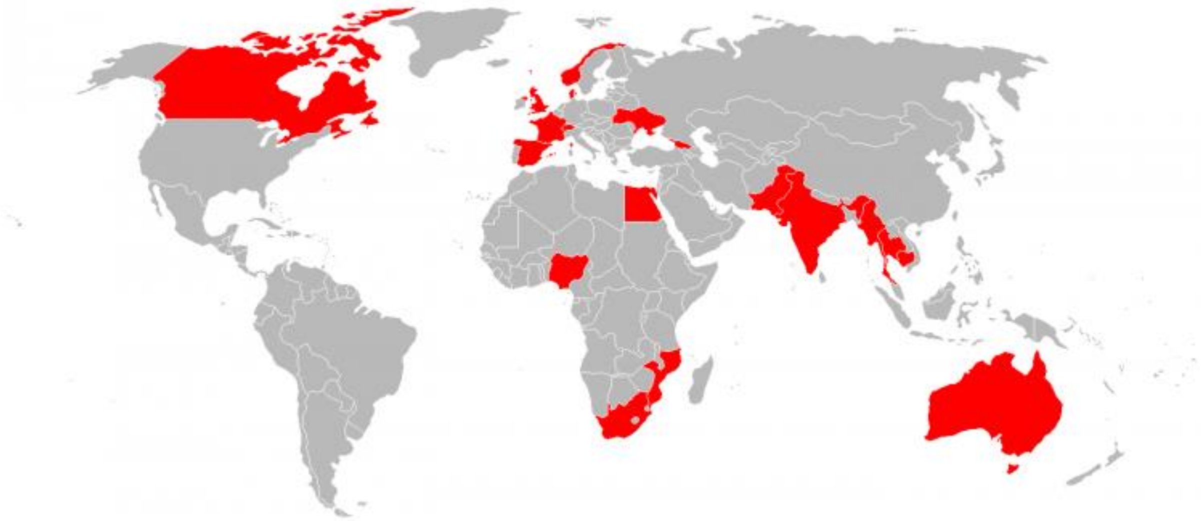

WHO region: **Africa** – Mozambique (n=2), Nigeria (n=1), South Africa (n=1). **Americas** – Canada (n=2). **Eastern Mediterranean** – Egypt (n=1), Pakistan (n=4). **Europe** – Denmark (n=1), France (n=4), Georgia (n=2), Norway (n=1), Spain (n=4), Switzerland (n=1), UK (n=8), Ukraine (n=1). **South-east Asia** – India (n=3), Myanmar (n=3), Thailand (n=1). **West-Pacific** – Australia (n=3), Cambodia (n=2)

2 (4%) from low-income countries, 19 (42%) from middle-income countries, and 24 (53%) from high-income countries)

**Supplementary table 6:** Detailed description of each included study

| Reference #; Author, Year, Location, Population | Description |
|---|---|
| **HOMELESS** | |
| 7<br>Bajis; 2019<br>Sydney, Australia<br>Homeless | **Testing approach:** Recruitment campaign at study site and clinic site staff encouragement. At enrolment participants received an education resource package and a $20 voucher.<br>**Serological testing:** None<br>**Viral load testing:** Fingerstick whole-blood GeneXpert RNA. Participants were not provided their GeneXpert results as not approved in Australia. GeneXpert results provided to clinic staff to inform subsequent follow-up. DBS venepuncture SOC samples used to confirm HCV infection. Enrolment visit also included survey and Fibroscan for liver disease staging by GP or nurse, and in some cases blood tests for routine clinical care.<br>**Linkage to care/assessment visit:** Following nurse/GP assessment, where applicable, a referral was made to a specialist for follow-up. All participants (including those negative) were asked to return 2-12 weeks post-enrolment to receive test results and for clinical follow-up (received further $20 voucher whether negative or positive). Where possible, RNA+ were scheduled for follow-up appointments. Linkage to care defined as clinical follow-up attendance.<br>**Treatment eligibility:** All RNA+ were evaluated for treatment and prescribed by a GP post March 2016. **Treatment initiation:** DAAs available in March 2016 (almost beginning of study: Feb). Treatment initiation by GP. (Not sure how long after assessment visit). **Treatment monitoring:** NA<br>**Other features:** At enrolment participants received an educational resource package and $20 voucher. |
| 8<br>London Joint Working Group on Substance Use and Hepatitis C (LJWG); 2020<br>London, UK<br>Homeless | **Testing approach:** Testing events targeted specific accommodation blocks where people with high-risk factors for HCV were temporarily being housed. In some cases, information about the pop-up testing was circulated before the visit.<br>**Serological testing:** PoC antibody test<br>**Viral load testing:** Two approaches used (a) GeneXpert, or (b) capillary RNA test<br>**Linkage to care/assessment visit:** Either testing procedure was followed by a Fibroscan if the test came back positive<br>**Treatment eligibility:** NA. **Treatment initiation:** For active infection, the area's operational delivery network (ODN) would be contacted to refer the patient with peer support to attend an outreach clinic led by the ODN. In many cases nurses from the relevant ODN area attended the testing days to facilitate this. **Treatment monitoring:** NA<br>**Other features:** NA |
| **PWID** | |
| 9<br>Chevaliez; 2020<br>Paris, France<br>PWID | **Testing approach:** Consecutive HCV-seropositive PWID were invited to participate in further evaluation by undergoing a clinical review with a liver assessment and blood tests including fingerstick capillary whole blood POC HCV RNA testing and fingerstick DBS sampling. Questionnaire was administered.<br>**Serological testing:** None as everyone was anti-HCV+.<br>**Viral load testing:** Whole blood RNA measured by GeneXpert fingerstick assay. Onsite. RNA also measured on capillary whole blood specimens collected using the DBS technique. Questionnaire was administered at same time. Genotype was also assessed and Fibroscan performed.<br>**Linkage to care/assessment visit:** NA<br>**Treatment eligibility:** NA. **Treatment initiation:** Elsewhere and on another day due to prescriber type restrictions. **Treatment monitoring:** Whole blood using DBS performed on the Roche or GeneXpert viral load assays.<br>**Other features:** Pregnant women excluded. Prescriber type restrictions for DAA therapy. |
| 10<br>Lens; 2020 | **Testing approach:** On site point of care antibody and RNA screening, liver stiffness measurement, treatment delivery and SVR12 assessment. Dried blood spot was collected at baseline, SVR12 and every 6 months in order to differentiate relapse vs reinfection. |

| | |
|---|---|
| Barcelona, Spain<br>PWID | **Serological testing:** Anti-HCV RDT<br>**Viral load testing:** If anti-HCV+ then test GeneXpert Fingerprick. Fibroscan at same time as GeneXpert<br>**Linkage to care/assessment visit:** NA<br>**Treatment eligibility:** NA. **Treatment initiation:** Onsite, possibly occurring on another day. Pan-genotypic regimens. **Treatment monitoring:** GeneXpert and DBS. Adherence assessed by daily or weekly visits.<br>**Other features:** Program included educative and harm-reduction interventions. |
| [11]<br>Lazarus; 2020<br>Copenhagen, Denmark<br>PWID | **Testing approach:** All clients were eligible for testing but only those with a legal residence in Denmark could be referred for treatment.<br>**Serological testing:** RDT In-Tech anti-HCV testing.<br>**Viral load testing:** GeneXpert fingerstick.<br>**Linkage to care/assessment visit:** Eligible HCV-RNA+ individuals were offered assisted referral to a fast-track hospital clinic for treatment, and peers provided assistance as needed.<br>**Treatment eligibility:** Those with a legal residence in Denmark. **Treatment initiation:** At hospital clinic. Treatment initiation is often between 1 to 4 weeks after the initial screening – depending on genotype results. Recently pan-genotypic medicine has been administered within a week. **Treatment monitoring:** NA<br>**Other features:** Among those initiating treatment, 14 were connected to drug treatment services and could be treated while receiving OST. The peer-led service assisted all treated with inter alia communication with the hospital nurse, collecting medicine, and accompaniment to follow-up visits. |
| [12]<br>Rogers; 2020<br>Leicestershire, England<br>PWID | **Testing approach:** Those who the pharmacy staff felt might benefit from a test could be directly invited or could themselves request a test.<br>**Serological testing:** None<br>**Viral load testing:** GeneXpert. Patients returned after 1-hour to receive their results or at an agreed alternative time. Patients were given £5 food voucher with completing a test and attending for the result.<br>**Linkage to care/assessment visit:** All positive patients were offered a nurse-led outreach clinic appointment (different day). There are a number of outreach clinics, which are usually within a drug treatment services setting. Individuals seen at whichever site is the most convenient for them (usually close to the pharmacy, very occasionally treated at the same site as they were tested).<br>**Treatment eligibility:** Pregnancy ineligible. **Treatment initiation:** Patients were then discussed at the multidisciplinary team meeting (which run every two weeks) and treatment prescribed by doctors there. **Treatment monitoring:** NA<br>**Other features:** Individual pharmacists received training on use of the GeneXpert platform, as well as the consent and testing process. |
| [13]<br>Antonini; 2018<br>Paris, France<br>PWID | **Testing approach:** Screening proposed to outpatients of an addiction center. On site screening was announced by the staff one month before.<br>**Serological testing:** RDT In-Tech anti-HCV Fingerstick (Toyo, Nephrotek)<br>**Viral load testing:** Fingerstick, GeneXpert. DBS to confirm HCV and assess HBsAg and HIV. HCV RNA results could be available the same day. DBS were processed in the clinical virology lab; results were given at the following consultation.<br>**Linkage to care/assessment visit:** Following results referred for specialized consultation with a hepatologist (elsewhere) within <2 weeks to consider treatment, but only 2 attended.<br>**Treatment eligibility:** NA. **Treatment initiation:** Specialized consultation with hepatologist. **Treatment monitoring:** NA<br>**Other features:** Patients were also asked to complete a short questionnaire. |
| [14]<br>Remy; 2019<br>Perpignan, France<br>PWID | **Testing approach:** Multidisciplinary mobile clinic deployed to various sites for outreach sessions in drug units, a local prison, and social care units<br>**Serological testing:** Patients identified using anti-HCV DBS tests, then nurse-facilitated referral to a dedicated test-to-treat session within 2 weeks<br>**Viral load testing:** GeneXpert.<br>**Linkage to care/assessment visit:** Fibroscan (same day), nurse-led pre-treatment counselling, group session: motivational interview, treatment education and harm reduction counselling, and DAA initiation<br>**Treatment eligibility:** NA. **Treatment initiation:** Same day RNA testing and starting treatment (given 1 month's worth on-site) through an on-site hepatologist, with a report sent to GP. **Treatment monitoring:** NA<br>**Other features:** NA |

| | |
|---|---|
| [15]<br>Bajis; 2020<br>New South Wales & Queensland & South Australia, Australia<br>PWID | **Testing approach:** Campaign days and recruitment posters at study sites. Info about study provided by staff at the centres and peer workers. Participants received $20 voucher at enrolment.<br>**Serological testing:** None<br>**Arm 1: No GeneXpert and no availability of subsidized unrestricted DAAs (pre early-2016)**<br>**Viral load testing:** Enrolment assessments included fingerstick RNA, DBS, survey, Fibroscan and nurse or medical practitioner assessment, and also provided venepuncture for SOC RNA testing to confirm HCV infection (as pre-GeneXpert registration period) and completed interview.<br>**Linkage to care/assessment visit:** Following Fibroscan, where applicable a referral was made to a specialist for follow-up. All participants were asked to return 2-12 weeks post-enrolment to receive test results and for clinical follow-up and interview. Linkage to care defined as clinical follow-up attendance. Participants received further $20 voucher upon completing follow-up interview.<br>**Treatment eligibility:** NA. **Treatment initiation:** No availability of subsidized unrestricted DAAs. **Treatment monitoring:** NA<br>**Other features:** NA<br>**Arm 2: GeneXpert and Subsidized unrestricted DAAs (early 2016 onwards – historical comparator without PoC RNA assay)**<br>**Viral load testing:** GeneXpert fingerstick RNA testing added to study procedures for participants recruited after 14/01/2016 (previously completed using in-house qualitative PCR on DBS collected by fingerstick). Enrolment assessments included survey, Fibroscan and nurse or medical practitioner assessment and completed interview.<br>Linkage to care/assessment visit: Following Fibroscan, where applicable a referral was made to a specialist for follow-up. All participants were asked to return 2-12 weeks post-enrolment to receive test results and for clinical follow-up and interview. Linkage to care defined as clinical follow-up attendance. Participants received further $20 voucher upon completing follow-up interview.<br>**Treatment eligibility:** Unrestricted. **Treatment initiation:** Introduced March 2016, so HCV+ participants were evaluated for treatment and subsequently prescribed treatment as per guidelines.<br>**Treatment monitoring:** NA<br>**Other features:** NA |
| [16]<br>Williams; 2019<br>Melbourne, Australia<br>PWID | **Testing approach:** Posters were displayed in the NSP to generate awareness for the study and clients were offered participation at the NSP desk and by staff.<br>**Serological testing:** Oraquick (oral fluid samples) – either self-collected or by nurse. Results returned to participants on-site after 20 minutes. Ab- test patients were offered standard lab testing for HCV confirmation (as neither POC tests used in this study were approved for diagnostic use in Australia).<br>Viral load testing: Those Ab+ underwent venepuncture for POC RNA, performed on-site by nurse or by self-collection. Tested using GeneXpert. Additional blood specimens were collected and sent for standard lab-based HCV testing and for all tests recommended for DAA treatment work-up in Australia. Patients could either remain at the clinic for the result (105 mins) or receive result by text/phone later that day, or on return to the clinic.<br>**Linkage to care/assessment visit:** All participants with blood sent for pathology testing had follow-up visit scheduled 1-2 weeks after POC tests. Assessed for treatment by nurse at the site. Liver fibrosis assessed using APRI<br>**Treatment eligibility:** Study criteria: 18+, not currently engaged in HCV care. **Treatment initiation:** Each site had specialist drug and alcohol services and GPs able to prescribe DAAs. At this follow-up visit RNA+ patients were assessed for HCV treatment (APRI, Fibroscan) by nurse and linked to GP within same service if there were no indications for specialist management. Where possible, the GP would review the participant directly after the nurse-assessment. Where not possible, an appointment was organised for a later date. **Treatment monitoring:** NA<br>**Other features:** Participants reimbursed $30 for time to participate in study (split after first and second) |
| [17] [18]<br>Valencia; 2019<br>Madrid, Spain<br>PWID/migrants/homeless | **Testing approach:** HCV and HBV rapid tests were conducted in locations where vulnerable population gather<br>**Serological testing:** Screening is carried out with a rapid test.<br>**Viral load testing:** Positive results confirmed within minutes using an onsite GeneXpert fingerstick. Test results returned within 58 minutes.<br>**Linkage to care/assessment visit:** If HCV or HIV then a PCR is performed and participants offered referral to hospital on same day – they are driven there.<br>**Treatment eligibility:** NA. **Treatment initiation:** The patient is able to take medication from the hospital pharmacy on the same day. **Treatment monitoring:** NA<br>**Other features:** Interdisciplinary health team. 65% homeless. |
| [19]<br>Saludes; 2020<br>Catalonia, Spain | **Testing approach:** Convenience sample. Questionnaire at enrolment used for data collection. Participants were asked to choose from the following options of receiving HCV-RNA results: receiving results on the same day, even if they had to wait for an hour, receiving results another day when returning to the center, or no expressed preference.<br>**Serological testing:** At recruitment in the drug consumption room, each participant underwent fingerstick capillary blood collection for: HCV Ab-RDT testing |

| | |
|---|---|
| PWID | **Arm 1: Xpert**<br>**Viral load testing:** At recruitment blood collection for HCV RNA-PoC testing. The personnel at the drug consumption room received direct support from the central laboratory in order to resolve incidents and remotely validate the results in real time. Comparing RNA testing by nursing staff at the drug consumption room with that done by DBS in the lab. Drug consumption room professionals delivered HCV viral load and HIV serology results to participants, performed post-test counseling, and referred participants to care when needed. As this study started before the Xpert Fingerstick assay obtained approval, a provisional report with Xpert fingerstick assay results was generated after remote validation from the laboratory as advised by Cepheid and delivered to participants on the same day of testing when possible. Confirmed results were available on the next working day (on Mondays) and delivered to participants at a second visit to the drug consumption room together with HIV serology results and the economic incentive.<br>**Linkage to care/assessment visit:** During this second visit, referral to care was initiated for all those who were viremic and were not already linked to care. Delivery of GeneXpert FS assay and reference method results as well as referral to care were recorded. No follow-up was performed to assess linkage to care after referral.<br>**Treatment eligibility:** NA. **Treatment initiation:** NA. **Treatment monitoring:** NA<br>**Other features:** The plasma samples were also used for HIV antigen/antibody testing, and for HCV antibody testing by conventional serology when an HCV Ab-PoC false negative result was suspected. Genotyping also performed.<br>**Arm 2: Comparator ("reference method"):**<br>**Viral load testing:** At recruitment blood collection for DBS collection for HCV-RNA detection and genotyping (comparator). See above for the rest of the details.<br>Linkage to care/assessment visit: See above - delivery of Xpert fingerstick assay and reference method results as well as referral to care were recorded.<br>**Treatment eligibility:** NA. **Treatment initiation:** NA. **Treatment monitoring:** NA<br>**Other features:** 6 euros was offered upon delivery of the confirmed test results obtained by the reference method. See above. |
| [20]<br>Schurch; 2020<br>Aargau, Switzerland<br>PWID | **Arm 1: GeneXpert cohort**<br>**Testing approach:** At study entry and yearly follow-up, free HIV/HCV antibody rapid testing, noninvasive liver fibrosis assessment and since August 2017 capillary HCV RNA rapid testing with the GeneXpert are offered to improve HCV screening and treatment uptake. Whenever possible, follow-up continues if patients discontinue OAT or leave the canton. These data are for new entrants to cohort who would be tested by GeneXpert.<br>**Serological testing:** Free HIV/HCV antibody rapid testing<br>**Viral load testing:** Since August 2017 capillary HCV RNA rapid testing with the GeneXpert are offered to improve HCV screening and treatment uptake. For POC testing, the GeneXpert must be on-site. Therefore, only cohort patients in a few centralised settings could benefit.<br>**Linkage to care/assessment visit:** NA<br>**Treatment eligibility:** NA. **Treatment initiation:** NA. **Treatment monitoring:** NA<br>**Other features:** "In cohort patients, who, since August 2017, benefit from capillary HCV RNA rapid testing with the GeneXpert® [29], this proportion was reduced to 11%. Even though the GeneXpert® is easily transportable in a passenger car, its use as a point-of-care test is restricted to centralised settings, because it is not cost efficient to visit every GP with only one to three OAT patients."<br>**Arm 2: Comparator cohort (historical data split by year, the early arm being without PoC RNA assay)**<br>**Testing approach:** For the decentralised settings, in June 2013 consent forms, questionnaires, and rapid antibody tests were sent to the physicians that provide OST. Flyers inviting participation were sent to the OST patients in pharmacies. Physicians received CHF 50 per patient for obtaining informed consent, performing the rapid tests and completing the questionnarire.<br>**Serological testing:** Rapid testing using capillary blood (OraQuick) for HCV and also HIV.<br>**Viral load testing:** NA (Pre-GeneXpert)<br>**Linkage to care/assessment visit:** Fibroscan scheduled after the rapid antibody test results at the outpatient clinic of the Cantonal Hospital Aarau. On some rare occasions the fibroscan was offered in the family practice on an appointed day.<br>**Treatment eligibility:** NA. **Treatment initiation:** NA. **Treatment monitoring:** NA<br>**Other features:** NA |
| [21,22]<br>Martel-Laferriere; 2019 | **Arm 1: Accelerated cohort** |

| | |
|---|---|
| Quebec, Canada<br>PWID | **Testing approach:** Patients (PWID >=18 years old, HCV infected but unaware if eligible for treatment, not linked to HCV care) identified and referred by their regular team (excluding patients already involved in HCV management).<br>**Serological testing:** None<br>**Viral load testing:** GeneXpert<br>**Linkage to care/assessment visit:** Medical assessment and fibroscan during same visit as GeneXpert. Then referral to family physician (for everyone) and harm reduction discussion and possible referral, and psychiatry referral if needed.<br>**Treatment eligibility:** Excluded if patient is unable to give consent, pregnant, already involved in HCV management, or has a defibrillator/pacemaker. **Treatment initiation:** If positive and treatment eligible they start treatment at 2nd visit (after reimbursement approval) - not including the anti-HCV testing. Those negative or treatment ineligible are followed up after 6 months. **Treatment monitoring:** NA<br>**Other features:** Variations in reimbursement criteria overtime: prior to March 2015: universal access, March 2015-March 2017: F3-F4, March 2017-March 2018: >=F2 or poor prognosis factors, Since March 2018: universal access.<br>**Arm 2: Historical comparator cohort from the CHUM addiction medicine clinic fulfilling the same inclusion criteria with usual standard of care (without PoC RNA assay) spanning several visits**<br>**Testing approach:** New patients seen at the CHUM addiction medicine clinic between Oct 2014 and Nov 2016. Patients in the historical control set meet the inclusion criteria for the study (except consent).<br>**Serological testing:** Many patients were already diagnosed before referral to the CHUM. Otherwise, antibody testing was performed at the CHUM central lab<br>**Viral load testing:** If positive, RNA was performed (second blood draw, not reflex) (in house)<br>**Linkage to care/assessment visit:** Medical assessment and fibroscan during. Then referral to family physician (for everyone) and harm reduction discussion and possible referral, and psychiatry referral if needed. Genotype is centralized in the provincial lab. CBC, INR, liver panel etc. performed at the CHUM lab. Blood draws were performed by nurses at the addiction clinic<br>**Treatment eligibility:** Excluded if patient is pregnant, already involved in HCV management, or has a defibrillator/pacemaker. **Treatment initiation:** NA. **Treatment monitoring:** NA<br>**Other features:** Variations in reimbursement criteria overtime: prior to March 2015: universal access, March 2015-March 2017: F3-F4 |
| [23]<br>Feld; 2019<br>Toronto, Canada<br>PWID | **Testing approach:** Offering rapid POC RNA testing to users of an SCS. An onsite HCV nurse completed baseline surveys with participants to capture socio-demographics and history of HCV care.<br>**Serological testing:** None<br>**Viral load testing:** HCV RNA testing conducted by nurse. Testing using capillary bloods and GeneXpert. Test results available in 60 mins.<br>**Linkage to care/assessment visit:** RNA +ve patients were immediately connected with an onsite HCV program and follow-up with the HCV program nurse.<br>**Treatment eligibility:** NA. **Treatment initiation:** Onsite HCV program. **Treatment monitoring:** NA<br>**Other features:** Staffed by nurses, health promoters and harm reduction workers with lived experience of drug use. Participants that tested negative were invited for repeat testing every 3 months for one year. |
| [24] [25]<br>Thingnes; 2019<br>Oslo, Norway<br>PWID | **Testing approach:** HCV testing offered along with the ordinary services during the study period.<br>**Serological testing:** Patients anti-HCV screened with rapid test (20 mins) at the mobile service.<br>**Viral load testing:** Anti-HCV +ve patients then offered GeneXpert VL test on capillary blood (60 mins).<br>**Linkage to care/assessment visit:** RNA +ve were offered a prescription at site. No patients stayed long enough to have both anti-HCV test and RNA test done at same visit. Anti-HCV +ve were attempted to be retrieved by phone or outreach (Hereafter all clients will be screened directly with the POC test) (No patients waited for results after POC testing either and time and resources were required to track patients afterwards).<br>**Treatment eligibility:** NA. **Treatment initiation:** NA. **Treatment monitoring:** NA<br>**Other features:** NA |
| [26]<br>Stone; 2019<br>South Yorkshire, UK<br>PWID | **Testing approach:** At risk patients in drug services and community were identified by peers, who accompanied patients to weekly afternoon hospital outpatient clinic.<br>**Serological testing:** None.<br>**Viral load testing:** Patients underwent fingerprick blood sampling and GeneXpert and/or HIV RNA POCT.<br>**Linkage to care/assessment visit:** HCV RNA +ve patients underwent specialist nurse assessment (inc fibroscan) on same day |

| | |
|---|---|
| | **Treatment eligibility:** Patients with Metavir F0-2 (median transient elastography (TE) ≤9.5kPa). **Treatment initiation:** On same day, patients with Metavir F0-2 (median transient elastography (TE) ≤9.5kPa) were assessed and counselled for HCV treatment with 12 weeks Sofosbuvir/Velpastasvir (Epclusa) and provided with the first 4 weeks of Epclusa at the same appointment, with a 4 week follow up appointment made. Patients with Metavir F3-4 (median transient elastography >9.5kPa) were given a separate appointment for an abdominal ultrasound, additional blood tests and medical assessment prior to treatment initiation. (No patient tested had detectable HIV RNA.) **Treatment monitoring:** NA<br>**Other features:** Peers identified PWIDs in the community and supported hospital outpatient clinic attendance. |
| [27]<br>Gutierrez (MSF); 2019<br>Mafalala, Mozambique<br>PWID | **Testing approach:** Community-based counselling and testing for HIV, HCV, and HBV.<br>**Serological testing:** RDT<br>**Viral load testing:** Linkage to CHAM in Maputo where GeneXpert was used<br>**Linkage to care/assessment visit:** NSP, TB screening campaigns, referral and counter-referral to health units for care and treatment (CHAM Maputo). Linking HIV, TB and HCV care and treatment in health units.<br>**Treatment eligibility:** NA. **Treatment initiation:** Done in Maputo (see study R37). **Treatment monitoring:** NA<br>**Other features:** Outreach for adherence support. |
| [28]<br>Butsashvili; 2019<br>Tblisi & Zugdidi & Batumi, Georgia<br>PWID | **Testing approach:** All PWID receiving services at harm reduction centers are offered anti-HCV testing.<br>**Serological testing:** All PWID receiving services at harm reduction centers are offered anti-HCV testing. Anti-HCV test is offered at the time of enrolment in the harm reduction program. Anti-HCV negatives are periodically offered anti-HCV re-testing.<br>**Viral load testing:** GeneXpert for confirmation available on-site for three of the sites.<br>**Linkage to care/assessment visit:** Before HCV treatment initiation, 3 visits are usually carried out.<br>**Treatment eligibility:** A simplified laboratory testing algorithm was introduced and patients having FIB4>1.45 are referred to specialized clinics for treatment while patients with FIB4<1.45 are treated at HR center. **Treatment initiation:** HCV infected PWID can start treatment on-site (at harm reduction center), if they are receiving services at the pilot HR centers with integrated care services, or at the regular specialized HCV treatment clinics. **Treatment monitoring:** NA<br>**Other features:** NA |
| [29]<br>Thuang 2020<br>Myanmar<br>PWID (but also other marginalized populations) | **Testing approach:** Patients were referred by peers, community workers, civil society groups, the General Practitioners Society, the National AIDS Program, the Myanmar Anti-Narcotic Association, MSF, and MDM. HCV treatment naïve or experienced. (pegIFN and RBV only). The intervention combined HCV and HIV testing, simplified HCV treatment, and HIV treatment initiation for those with HIV co-infection not yet on ART. Some were already known to be anti-HCV+. Laboratory results were also obtained from the medical record if reported within the specified window (namely HCV RNA at any time prior to study entry).<br>**Serological testing:** SD bioline and HIV antibody testing. Also tested for haemoglobin, platelets, AST, ALT, bilirubin, albumin, creatinine, blood ureas nitrogen, pregnancy, prothrombin, HBV, CD4.<br>**Arm 1: Viral load testing (Kachin Asian Harm Reduction Network):** HCV RNA testing undertaken for all with anti-HCV+ results. Confirmation was via near POC GeneXpert. Then referral for treatment. The viral load testing did not occur on the same day as serological testing. GeneXpert was performed on site at the clinic in Kachin. In most circumstances – the serological tests were done by referring clinics. Clinical decisions were based on the viral load testing.<br>**Arm 2: Viral load testing (Myanmar Liver Foundation; Mandalay):** HCV RNA testing undertaken for all with anti-HCV+ results. Confirmation was via near POC GeneXpert and/or Roche assay. Then referral for treatment. The viral load testing did not occur on the same day as serological testing. GeneXpert and Roche was performed at lab in Yangon for the Yangon and Mandalay sites. In most circumstances – the serological tests were done by referring clinics. Clinical decisions were based on the viral load testing. Roche served as standard of care VL assay prior to validation of GeneXpert. Results related to patients tested by Roche rather than GeneXpert are not included in the extracted data.<br>**Arm 3: Viral load testing (Myanmar Liver Foundation; Yangon):** HCV RNA testing undertaken for all with anti-HCV+ results. Confirmation was via near POC GeneXpert and/or Roche assay. Then referral for treatment. The viral load testing did not occur on the same day as serological testing. GeneXpert and Roche was performed at lab in Yangon for the Yangon and Mandalay sites. In most circumstances – the serological tests were done by referring clinics. Clinical decisions were based on the viral load testing. Roche served as standard of care VL assay prior to validation of GeneXpert. Results related to patients tested by Roche rather than GeneXpert are not included in the extracted data. |

| | |
|---|---|
| | **Linkage to care/assessment visit:** Medical history and clinical assessment were undertaken at baseline. Pre-treatment assessment included clinical exam, APRI, blood investigation like CP, liver function and renal function, and mHealth linkage to care. The pre-treatment visit occurred on the same day as the serological test was done BUT the treatment initiation was done on a separate day. Genotyping also performed in the lab in Yangon.<br>**Treatment eligibility:** Could have previous experience of pegIFN and RBV. Ineligible HCV+ patients identified through screening are eligible for an observation arm. Excluded decompensated cirrhosis or end stage renal disease. Aged 18+ with or without HIV co-infection. Active TB excluded, or known allergy/hypersensitivity to components of drugs, end stage renal disease, prior DAA treatment. **Treatment initiation:** DAAs. **Treatment monitoring:** GeneXpert for VL monitoring.<br>**Other features:** The sites with lower SVR12 percentages had higher proportions of PWID (62% overall) |
| [30]<br>HEADSTART; 2020<br>Manipur, India<br>PWID | **Testing approach:** Adults with a past or current history of injecting drug use and their sexual partners were consecutively enrolled on the study. Non-residents, children, or patients already diagnosed with HCV or already initiated on treatment for the management of HCV infection were excluded. The two study clinics were established for the purposes of this study. The three HRS in the study were already established centres. The outreach screening involved peer educators raising awareness of HCV services for the study and to offer screening and recruitment to the study.<br>**Arm 1: Study sites**<br>**Serological testing:** Anti-HCV RDT at study site.<br>**Viral load testing:** Reflex or next day venous sample for HCV viral load and sample for pre-treatment assessment bloods. HCV viral using GeneXpert. Also, at study site.<br>**Arm 2: Harm reduction sites/ICC sites**<br>**Serological testing:** Anti-HCV RDT at harm reduction site<br>**Viral load testing:** Next visit, with same day result to patient and referral to study site for venous sample for HCV viral and sample for pre-treatment assessment bloods. HCV VL using GeneXpert. If RNA +ve email/print report from private lab to study clinic sites, phone result to patient.<br>**Arm 3: Outreach**<br>**Serological testing:** Anti-HCV RDT at harm reduction site - outreach testing also done<br>**Viral load testing:** Next visit, with same day result to patient and referral to study site for venous sample for HCV VL and sample for pre-treatment assessment bloods. HCV VL using GeneXpert. If RNA +ve email/print report from private lab to study clinic sites, phone result to patient.<br>**Linkage to care/assessment visit:** Reflex use of existing sample for pre-treatment bloods, with pre-treatment assessment blood assays at a private lab (HBsAg, complete blood count, liver function tests, creatinine).<br>**Treatment eligibility:** NA. **Treatment initiation:** Treatment assessment (doctor consultation) and initiation visit. **Treatment monitoring:** Venous sample, GeneXpert at study sites<br>**Other features:** NA |
| [31,32]<br>HEADSTART; 2020<br>Georgia<br>PWID | **Testing approach:** Participants eligible for study if they tested HCV+ on the same day and did not have a prior confirmed diagnosis.<br>**Serological testing:** RDT at harm reduction centre<br>**Arm 1: GeneXpert**<br>**Viral load testing:** Same day reflex venous sample for HCV VL with same day onsite GeneXpert VL and result to patient both at harm reduction centre.<br>**Linkage to care/assessment visit:** Referral to designated treatment centre for initial assessment visit and pre-treatment assessment bloods.<br>**Treatment eligibility:** NA. **Treatment initiation:** Separate visit for treatment initiation. (3 visits in total to treatment start). **Treatment monitoring:** Venous sample SVR12 at designated treatment centres with sample transport to designated labs.<br>**Other features:** NA<br>**Arm 2: Centralised cAg, no PoC RNA assay**<br>**Viral load testing:** Same day reflex venous sample for HCV VL (with sample transport to reference centre), core antigen assay at reference center. Results uploaded into national database with phone/in-person result to patient.<br>**Linkage to care/assessment visit:** Then initial assessment visit and pre-treatment assessment bloods on second visit<br>**Treatment eligibility:** NA. **Treatment initiation:** Separate visit for treatment initiation (3 visits in total to treatment start). **Treatment monitoring:** Venous sample SVR12 at designated treatment centres with sample transport to designated labs.<br>**Other features:** NA |

| | |
|---|---|
| | **Arm 3: SOC**<br>**Viral load testing:** Referral to designated treatment centre for HCV VL sample collection with sample transport to designated lab (visit 2). VL results go from lab to clinic then clinic to patient.<br>**Linkage to care/assessment visit:** If RNA +ve they have initial assessment visit and pre-treatment assessment bloods.<br>**Treatment eligibility:** NA. **Treatment initiation:** Separate visit for treatment initiation (4 visits in total to treatment start). **Treatment monitoring:** Venous sample SVR12 at designated treatment centres with sample transport to designated labs.<br>**Other features:** NA |
| [33 34]<br>Draper, HEADSTART; 2020<br>Myanmar<br>PWID | **Testing approach:** For Burnet site, participants recruited by peer worker at methadone centres or PWID networks or through direct presentation at the clinic for needle/syringe services. For the MLF site, participants were recruited through recall from the patient registration list, including patients already registered for upcoming no-cost programs at the clinic. Participants were also recruited from patients attending the site on study recruitment days. Participants had not previously tested positive for HCV RNA and had no prior treatment or HIV, HBV, TB co-infection.<br>**Serological testing:** RDT at study sites using whole blood venous samples).<br>**Viral load testing:** Confirmation via GeneXpert RNA testing on-site.<br>**Linkage to care/assessment visit:** If HCV RNA+ they have pre-treatment assessment using remaining blood samples: HIV RDT, HBV RDT, ALT, AST, bilirubin, albumin, ALP, platelets, haemoglobin, creatinine, eGFR. RDTs were conducted on site and other blood tests were conducted at nearby private, external lab with samples collected from study sites daily and results returned via email within 24 hours. Also, APRI score used to assess fibrosis and inform treatment length. Patient return visit to Study Clinic for pre-treatment results – often the day after their RNA test<br>**Treatment eligibility:** Participants with (1) ALT or AST >200 U/L, (2) bilirubin above upper limit of normal (1.14mg/dL), (3) albumin <35 units without other obvious cause, (4) jaundice, (5) ascites, (6) hepatic encephalopathy or (7) haematemesis and melena were referred to a hepatologist for review. **Treatment initiation:** If results of pre-treatment tests are uncomplicated then patients initiate treatment at the study clinic on same day, whilst they initiate treatment with specialists if the results of the pre-treatment tests are complicated. Pre-treatment start counselling. Medication dispensed onsite. **Treatment monitoring:** GeneXpert for SVR12<br>**Other features:** NA |
| [35,36]<br>London Joint Working Group on Substance Use and Hepatitis C (LJWG); 2019<br>London, UK<br>PWID | **Testing approach:** Pharmacy staff ask PWID using NSP if they would like HCV test.<br>**Arm 1: Pilot 1 – historical comparator**<br>**Serological testing:** Oraquick<br>**Viral load testing:** Refer to hospital for further tests and treatment<br>**Linkage to care/assessment visit:** Positive tests referred to hospitals for further tests and treatment.<br>**Arm 2: Pilot 2**<br>**Serological testing:** None<br>**Viral load testing:** GeneXpert capillary blood test. Client can either wait for results or organise a follow-up appointment.<br>Linkage to care/assessment visit: Positive tests referred to hospitals for further tests and treatment.<br>**Treatment eligibility:** NA. **Treatment initiation:** NA. **Treatment monitoring:** NA<br>**Other features:** Some PWID were also receiving OST. Service users were provided with information on HCV, current HCV treatment and safer injecting practices. Additionally, all PWID tested were provided with a £5 contingency voucher upon receipt of their result. |
| [37]<br>SOS Hepatitis BFC team; 2020<br>Burgundy, France<br>PWID | **Testing approach:** Mobile unit visits various places where the users had already been informed that the visit would take place.<br>**Serological testing:** RDT.<br>**Viral load testing:** GeneXpert<br>**Linkage to care/assessment visit:** Fibroscan also performed for all at same time as other tests.<br>**Treatment eligibility:** No cirrhosis, no comorbidities. **Treatment initiation:** NA. **Treatment monitoring:** NA<br>**Other features:** Issues with getting enough blood for GeneXpert. A doctor was always present who could prescribe. Also tested for HIV and syphilis. GeneXpert also used to investigate reinfections in those believing themselves cured of HCV. |

| | |
|---|---|
| 38<br>Morris; 2020<br>West Midlands, UK<br>Homeless/PWID | **Testing approach:** 14 pre-arranged testing sessions. Those identified at risk were offered anti-HCV testing and were subsequently tested for RNA.<br>**Serological testing:** Rapid antibody tests (Orasure swab testing)<br>**Viral load testing:** GeneXpert on the same day<br>**Linkage to care/assessment visit:** NA<br>**Treatment eligibility:** None. **Treatment initiation:** All RNA +ve individuals were referred to a hospital hepatology multidisciplinary team for treatment. Treatment was at the testing venue or another setting requested by the patient, usually within 2 weeks but with some local variation. **Treatment monitoring:** NA<br>**Other features:** NA |
| 39<br>Wansom (C-Free Study); 2021<br>Bangkok & Chiangmai & Songkhla & Narathiwat in Thailand<br>PWUD | **Testing approach:** Offering HIV, HBV, and HCV testing every 3 months. Former or active injecting or non-injecting drug users or their partners. Patients referred by community outreach workers, hospitals, and other participants.<br>**Serological testing:** On-site antibody testing for HIV, HCV, and HBV<br>**Viral load testing:** GeneXpert for HIV, HCV, and HBV located at the community drop-in center.<br>**Linkage to care/assessment visit:** NA<br>**Treatment eligibility:** Participants with HCV infection without evidence of decompensated cirrhosis, hepatocellular carcinoma, or end stage renal disease, were offered a 12-week course of SOF/VEL. **Treatment initiation:** If they were found to have HCV and met eligibility criteria immediately at enrolment; they would be scheduled to a day with physician clinic at the community drop-in and could start treatment at that visit at community drop-in centers in weekly visits by physicians **Treatment monitoring:** NA<br>**Other features:** NA |
| 40<br>Sonderup; 2021<br>Pretoria, South Africa<br>PWID | **Testing approach:** Point of service model among PWID. Community-based recruitment from Pretoria's PWID population occurred over 11 months.<br>**Serological testing:** Point of service HIV/HCV/HBV screening (Oraquick)<br>**Viral load testing:** HCV PCR testing on site (Genedrive). At baseline, an HCV RNA 'detected' confirmed viremia as part of eligibility for treatment assessment.<br>**Linkage to care/assessment visit:** NA<br>**Treatment eligibility:** NA. **Treatment initiation:** Treatment initiated on-site in same facility on same day. If HIV+ and not on ART, then ART was initiated and HCV treatment delayed for 3 months.<br>**Treatment monitoring:** GeneDrive<br>**Other features:** OST support with food parcels. Harm reduction and adherence support through directly observed therapy, peer support, a stipend and transport, was provided. Four GeneDrive units available. |
| **NON-SPECIFIC/MIXED POPULATION GROUPS** | |
| 41<br>Agwuocha; 2019<br>Nasawara state, Nigeria<br>Non-specific population | **Testing approach:** Using existing GeneXperts set up for TB to test for HCV viral load in 6-month pilot.<br>**Serological testing:** Laboratory personnel (Medical Laboratory Scientists, Medical Laboratory Technicians, Medical Laboratory Assistants) perform the serology testing using antibody RDTs which are offered at Health Facilities across Nigeria.<br>**Viral load testing:** GeneXpert. (During the pilot GeneXpert viral load was performed at Dalhatu Araf Specialist Hospital, a tertiary hospital in Nasawara State Nigeria). Routine viral load using GeneXpert is now performed at 8 hospitals in Nasarawa state. 4 of these sites also offer treatment. Also looked to support decentralization of diagnostic and treatment access through near POC testing at 1 tertiary hospital. Some primary and secondary facilities for referral testing. Time to RNA result receipt for patient 1-2 days<br>**Linkage to care/assessment visit:** After patients are confirmed viremic, in addition to clinical examination, patients are sent for a minimum of tests to enable APRI score calculation. All state facilities have existing and functional clinical chemistry labs which provide access to this testing (no additional support is provided by the program). These tests were performed by the laboratory personnel. However, prior to the program inception and HCV integration, patients had to access VL services and genotyping outside of the state, mainly in private facilities. But the test mentioned above were provided at the state facilities. |

| | |
|---|---|
| | **Treatment eligibility:** Prioritised people with cirrhosis, high-risk groups, and those with HIV or HBV but did not seem to exclude people. **Treatment initiation:** Routine treatment now occurs in 2 Tertiary sites in Lagos, 1 Tertiary site in the Federal Capital Territory, 1 Tertiary site in Rivers state and 10 Tertiary and Secondary sites in Nasawara state (4 of these treatment sites also offer GeneXpert VL). **Treatment monitoring:** NA<br>**Other features:** Increased lab hours from 8 to 12 to accommodate HCV tests. The high out of pocket expenses associated with the medication remain a barrier to treatment access. Prior to HCV integration and implementation of the public health approach, treatment was limited to the specialist doctors. |
| [42]<br>MSF; 2020<br>Uttar Pradesh, India<br>Non-specific population | **Testing approach:** NA<br>**Serological testing:** RDT (anti-HCV, HIV, HBV) at district and medical teaching hospital site<br>**Viral load testing:** Decentralized diagnostics at district hospital site, POC viral load from GeneXpert, minimising number of visits for patient, lesser number of tests for patients with APRI<1. RDT (anti-HCV, HIV, HBV) viral load, blood tests (more if APRI>1). Staging of liver disease with APRI (AST and platelet count). If APRI>1 – additional blood tests.<br>**Linkage to care/assessment visit:** NA<br>**Treatment eligibility:** NA. **Treatment initiation:** Started on treatment also at district hospital. **Treatment monitoring:** NA<br>**Other features:** Describes old model of care: treatment of APRI<2 cases at treatment centres, treatment of APRI>2 cases at model treatment centres (but data unavailable). Complicated and costly initial testing for all patients. RDT then viral load at model treatment centres, treatment of APRI<2 in district hospitals, treatment of complicated cases in medical colleges. Have improved retention of care through POC diagnostics, reducing visits from 14 initially to 5. High number of patients coming for screening initially vs limited diagnostic capacity. Moved from test and treat to test all but treat only APRI>1 leaving many without treatment. |
| [43]<br>MSF – Walker; 2018<br>Phnom Penh, Cambodia<br>Mixed population | **Arm 1: Full care model - First 6 months of the program, containing comprehensive testing, and consultations (September 2016 – March 2017 – historical comparator)**<br>**Testing approach:** Clinic was run by MSF only for HCV patients on the grounds of a national hospital. Patients referred from hepatologists (they self-presented and were briefly seen by a hepatologist before being screened, due to hospital policies). Also, PLHIV and at-risk populations.<br>**Serological testing:** Visit 1: HCV pre-test counselling, ELISA test for HCV antibody.<br>**Viral load testing:** Visit 2: ELISA disclosure and counselling, HCV PCR VL test. (External lab used for ELISA and PCR confirmation and another for genotyping) APRI scoring<br>**Linkage to care/assessment visit:** Visit 3: PCR disclosure and counselling. Visit 4: Genotype test. Visit 5: Fibroscan performed by physician. Visit 6: Pre-treatment assessment and counselling, baseline blood test, pregnancy test. Visit 7: Family planning + HIV test. Visit 8: Endoscopy and ultrasound.<br>**Treatment eligibility:** Fibrosis restrictions. **Treatment initiation:** Visit 9: Treatment initiation. **Treatment monitoring:** 8 consultations, with 6 patient education sessions. Extra labs were taken. Two SVR12 visits.<br>**Other features:** Task-shifting to nurses<br>**Arm 2: Simplified model - Gradually simplified diagnostic pathway, decreasing number and types of tests, counselling (August 2017 to April 2018)**<br>**Testing approach:** Patients referred from hepatologists. Also PLHIV and at-risk populations.<br>**Serological testing:** Visit 1: POC serology & PCR & Fibroscan & RDT disclosure. (SD Bioline and GeneXpert were the POC tests used)<br>**Viral load testing:** (See visit 1)<br>**Linkage to care/assessment visit:** Visit 2: PCR disclosure, pre-treatment assessment and tests.<br>**Treatment eligibility:** All. **Treatment initiation:** Visit 3: Treatment initiation. **Treatment monitoring:** 2 consultations. 1 patient education session (at initiation). One SVR12 visit.<br>**Other features:** Task-shifting to nurses and pharmacists. (There was a transition phase of March 2017 to August 2017 between the two models) |
| [44]<br>Khalid (MSF); 2020<br>Karachi, Pakistan<br>Non-specific/high-risk population | **Testing approach:** All patients presenting to the primary health care clinic passed by the triage desk, except for pregnant patients, who went directly to the maternity unit. At the triage desk, patients were directed to the proper unit (general outpatient dept, HCV clinic, or vaccination unit). All the patients who were sent to the outpatient department were then routinely assessed for HCV risk factors by outpatient department doctors. Patients with signs/symptoms/risk factors were referred to the lab (within the clinic) for HCV screening.<br>**Serological testing:** Oraquick RDT<br>**Arm 1: Pre-Feb 2017 - Arm population divided into 2 groups: RDT pre-October 2016, APRI cut-off >1; RDT October 2016 to January 2017, APRI cut-off >0.5 – historical comparator, no PoC RNA**<br>**Viral load testing:** RDT +ve patients were then tested by PCR. RDT +ve patients first tested by qualitative PCR at external lab, if +ve patient underwent APRI scoring and then testing for VL through quantitative PCR (again at external lab). |

| | |
|---|---|
| | **Linkage to care/assessment visit:** If PCR +ve they were referred to HCV unit (within same premises) for assessment by HCV doctor and treatment initiation (eligibility based on APRI, mental health, not pregnant). APRI score used to determine fibrosis stage.<br>**Treatment eligibility:** APRI >1 was cut-off for treatment eligibility until Oct 2016, then decreased to >0.5. **Treatment initiation:** NA. **Treatment monitoring:** NA<br>**Other features:** NA<br>**Arm 2: After February 2017 - RDT February 2017 to September 2017, APRI cut-off >0.5 with testing by GeneXpert.**<br>**Viral load testing:** Post Feb 2017, RDT+ patients were offered PCR testing internally in the clinic using GeneXpert VL on the same day as the RDT, followed by APRI staging.<br>**Linkage to care/assessment visit:** NA<br>**Treatment eligibility:** APRI cut-off >0.5. **Treatment initiation:** Not on same visit. **Treatment monitoring:** NA<br>**Other features:** The treatment initiation censoring date (28th November 2017) will affect comparisons. |
| [45]<br>Qureshi; 2018<br>Karachi, Pakistan<br>Non-specific population | **Testing approach:** Patients suffering from HCV either as treatment naïve or relapsed or non-responders who had detected HCV RNA were selected as cases.<br>**Serological testing:** Patients are screened using HCV RDT and those found positive are checked for virus using PCR. However, many patients present as HCV RNA+.<br>**Viral load testing:** PCR was done using GeneXpert on the day of consultation and results were made available within 2 hours.<br>**Linkage to care/assessment visit:** On same day as GeneXpert blood tests (complete blood count, ALT, AST) and ultrasound were done.<br>**Treatment eligibility:** NA. **Treatment initiation:** Patients offered treatment for 6 months. The treatment of HCV+ patients starts once all the reports are received i.e. PCR and other lab tests. Patients are called on the next day. **Treatment monitoring:** First follow-up done at 5-6 weeks when blood tests and PCR were repeated and clinical and virological response were reassessed. Second and third follow-up were done at 4 and 6 months and the last follow-up was SVR12.<br>**Other features:** At private clinic cost of diagnosis and treatment borne by patients, whilst at civil society organisations it's borne by a non-governmental organization. First follow-up was done at 5-6 weeks into treatment when blood tests and PCR were repeated. Test results available within 2 hours. One window solution to all patients i.e., test and treatment are conducted in the same location. |
| [46]<br>Qureshi; 2020<br>Islamabad, Pakistan<br>Non-specific/high-risk population | **Testing approach:** Community health workers (CHWs) visit every dwelling in the slum and offer household members aged ≥18 screening for HCV by a rapid antibody test.<br>**Serological testing:** Rapid antibody test<br>**Viral load testing:** Those that test positive are referred to an established clinic for diagnosis of viral load by GeneXpert. RNA results are made available to participants within 2 hours.<br>**Linkage to care/assessment visit:** If RNA+, additional blood is obtained and tested to calculate APRI. Subjects then receive counselling and their first 4-week supply of treatment and the first of three doses of HBV vaccine during initial clinic visit.<br>**Treatment eligibility:** NA. **Treatment initiation:** Same visit as assessment. **Treatment monitoring:** Patients seen every 4 weeks at the clinic and given refills.<br>**Other features:** Free of charge hepatitis C testing and treatment and utilizes trained community health workers (CHWs). None of the 126 who initiated treatment had been previously tested or treated for HCV. |
| [47-49]<br>Hamid; 2019<br>Karachi, Pakistan<br>Non-specific/high-risk population | **Arm 1: Community camp (GeneXpert) – historical comparator**<br>**Testing approach:** Community awareness (flyer distribution, education seminars).<br>**Serological testing:** Fingerstick RDT carried out in community screening camps (room in community centre/mosque)<br>**Viral load testing:** Bloods sent to hospital for GeneXpert (2 cartridge) and assessment tests. RNA could not be made a true point-of-care test, due to lack of basic facilities in the community, like stable electric power supply. Moreover, blood samples had to be carried some distances to a central testing facility, which results in some delay in linkage to care. The samples were initially collected at a nearby lab facility and then at the screening camps. The GeneXpert platform was located at the main university campus approximately 30km away.<br>**Linkage to care/assessment visit:** Blood draw for complete blood count, ALT, AST, Creatinine, HBsAG – used to calculate APRI, FIB4. Tests at university campus.<br>**Treatment eligibility:** NA. **Treatment initiation:** If positive then people were called back to the community camp to start the treatment when staff were next around. **Treatment monitoring:** NA<br>**Other features:** Initial efforts at linkage to care were not fruitful. Even with the subsidized treatment as part of the Extended Community Health Outcomes (ECHO) program, many people did not accept treatment due to multiple reasons; Non-affordability, Stigma associated with HCV infection, Fear and denial of the disease, Difficulty to travel (even in a radius of 5 km to treatment sites), particularly for females due to their dependence on male family members for transportation, And preference to seek care through traditional healers easily available in the community.<br>**Arm 2: House-to-house screening (Non-GeneXpert)** |

| | |
|---|---|
| | **Testing approach:** Going house-to-house in the community and taking blood samples<br>**Serological testing:** Rapid test<br>**Viral load testing:** Abbott in district hospital lab, which was closer to the community than the university lab. Was initially in the same lab as the community camps and used GeneXpert but this was deemed impractical.<br>**Linkage to care/assessment visit:** From blood draw for RNA.<br>**Treatment eligibility:** NA. **Treatment initiation:** At the person's house (returned after lab tests). **Treatment monitoring:** Went back each month to check for compliance to treatment<br>**Other features:** NA |
| [50]<br>Shiha; 2020<br>Dakahlia and Cairo, Egypt<br>Non-specific/high-risk population | **Testing approach:** Pre-study awareness campaigns at both sites.<br>**Serological testing:** Anti-HCV and HBsAg screening done using RDTs.<br>**Viral load testing:** Same day GeneXpert for PCR (venous at site 1 and fingerstick at site 2 for HCV). Also tested for HBV RNA using GeneXpert – at both sites additional venous sample was taken for additional tests. Anti-HCV and HBsAg 20 mins. PCR 105 mins at site 1, 59 mins at site 2 for HCV (105 mins for HBV). Two arms are used, both with PoC RNA assay, for different sites within the project<br>**Linkage to care/assessment visit:** Same day Fibroscan and abdominal ultrasound for staging of disease and screening for hepatocellular carcinoma. Then clinical assessment, screening for non-communicable diseases and treatment counselling (also same day). Blood counts, liver functions etc within 60 mins. Fibroscan 15 mins, ultrasound 15-20 mins.<br>**Treatment eligibility:** Excluded those with focal hepatic lesions and pregnant women as both required further examination. **Treatment initiation:** Same day treatment initiation for both HCV and HBV. **Treatment monitoring:** Follow-up in the village for site 1 or by the medical sector of the State office for site 2.<br>**Other features:** NA |
| [51]<br>Zhang (MSF), 2021<br>Battambang, Cambodia<br>Non-specific population | **Testing approach:** Voluntary HCV screening was initiated for (almost) all patients presenting at health center. The health centers and hospital imposed a service fee for screening and consultation which was waived for patients holding Cambodian government identification card for low wage individuals. Non-residents of Moung Russei not included in data. People aged ≥18 eligible for screening, regardless of previous HCV treatment experience. PLHIV were referring to national center for HIV/AIDS. Pregnant women and people with TB also ineligible.<br>**Serological testing:** Pre-test counselling, then RDT at any of the sites.<br>**Viral load testing:** If positive, venous blood sample was drawn immediately and transported same day to referral hospital lab for viral load testing by GeneXpert. Viral load results informed via telephone the same day test results were available, and a consultation at the HCV clinic for treatment eligibility assessment was scheduled at earliest available appointment.<br>**Linkage to care/assessment visit:** At HCV clinic. Fibroscan, serum creatinine for some subgroups, ALT for HbsAg+ patients,<br>**Treatment eligibility:** None. **Treatment initiation:** Simple pre-treatment evaluation performed at HCV clinic. Differentiated care based on simple of complicated cases (decompensated cirrhosis, previous DAA, HBV, or other comorbidities). **Treatment monitoring:** Differentiated follow-up performed at either the health centers or HCV clinic depending on complexity of case. From March 2019 patient tracing was stopped for patient not presenting for SVR12 testing due to high workload this caused.<br>**Other features:** MSF provided tailored training on HCV diagnosis, treatment, and patient management for clinical staff identified as responsible for HCV care from existing staff at the health centers and referral hospital. All screening material and DAAs supplied by MSF. Four nurse supervisors were hired by MSF to coordinate patient management. Some changes to the assessment tests performed were made during the project as well as changing the definition of HCV VL positive from >10 to >1000IU/mL. |
| **PRISONERS** | |
| [52]<br>Mohamed; 2020<br>London, UK<br>Prisoners (on OST in the intervention arm) | **Arm 1: Opt-out screening pathway (In place since early 2017 – historical comparator)**<br>**Testing approach:** Universal opt-out policy for new arrivals to the prison<br>**Serological testing:** Primary health screen (including BBV testing) performed on arrival in prison. DBS subsequently performed by healthcare assistant (prequalification nurse practitioner) during a more comprehensive secondary health screen in prison wing (within 7 days of arrival). DBS samples delivered by mail to the lab at a hospital lab elsewhere. DBS samples processed for HIV, anti-HCV, and HBV.<br>**Viral load testing:** Reflex testing performed for all sero+ samples, in case of HCV Ab+ both qualitative RNA and genotype measurements performed. Results uploaded on electronic patient record. |

| | |
|---|---|
| | **Linkage to care/assessment visit:** Between Sept 2017 and Dec 2018 there was a twice monthly prison hepatology clinic. At assessment visit, blood test done including blood, liver, renal tests and HCV RNA test (DBS) and Fibroscan. Clinician led.<br>**Treatment eligibility:** All eligible inmates were approved DAAs at a bi-monthly team meeting. Individuals with sentence long enough to complete treatment were started on treatment, whilst those with shorter sentences were encouraged to access treatment in the community. **Treatment initiation:** Treatment dispensed by the hospital pharmacy and delivered to the prison in monthly instalments. Individuals with sentence long enough to complete treatment were started on treatment, whilst those with shorter sentences were encouraged to access treatment in the community.<br>**Treatment monitoring:** NA<br>**Other features:** NA<br>**Arm 2: Rapid POC pathway for those on OST**<br>**Testing approach:** Between Sep & Dec 2018, individuals on OST were offered this new pathway (as well as the other). Individuals requiring OST were triaged to the substance misuse unit within 24 hour of prison arrival.<br>**Serological testing:** A specific BBV assistant responsible for all pre-test counselling and rapid testing (Oraquick). As part of induction assessment healthcare assistant did anti-HCV saliva swab with result within 20 mins.<br>**Viral load testing:** Imperial hepatology team informed of an Ab+ result and patient was assessed within one week of referral. At assessment visit, blood test done including blood, liver, renal tests and fibroscan. Clinician led. Additionally, venepuncture sample obtained from each HCV seropositive patient. On the same day as sample collection, 100µl of venous blood was pipetted directly from one of the samples into the GeneXpert Fingerstick assay by a clinician. Time to result was 60 min. The corresponding sample was sent to the local virology laboratory for standard of care plasma HCV viral load and genotyping<br>**Linkage to care/assessment visit:** NA<br>**Treatment eligibility:** All patients with +ve GeneXpert result were offered DAAs irrespective of sentence length. Those who agreed were approved via fast-track team meeting. **Treatment initiation:** Medication dispensed monthly. Appointment made at the hospital for those released before end of treatment. **Treatment monitoring:** NA<br>**Other features:** Used RDT testing and fast-tracked clinical assessment and treatment approval at team meeting |
| [53]<br>Davies; 2020<br>Swansea, England<br>Prisoners | **Arm 1: Rapid pathway**<br>**Testing approach:** Individuals screened on admission with a two-tiered point of care testing strategy.<br>**Serological testing:** All admissions were first screened for antibodies using a mouth swab (OraSure) on second day of arrival (secondary health screen)<br>**Viral load testing:** Antibody reactive individuals were then tested for PCR using a capillary blood sample (GeneXpert fingerstick). Same day as serological testing<br>**Linkage to care/assessment visit:** NA<br>**Treatment eligibility:** NA **Treatment initiation:** PCR positive individuals were immediately referred for treatment to the Health Board Bloodborne virus team. Pan-genotypic therapy. **Treatment monitoring:** NA<br>**Other features:** The imprisoned men have work commitments within the prison making follow-up difficult. Also, project delivered with no extra staff, so increased staff workload. The 418 individuals not tested were either recently tested or known positives, missed testing or declined testing (some were released before this was possible).<br>**Arm 2: Historical comparator without PoC RNA assay**<br>**Testing approach:** NA<br>**Serological testing:** Prior to starting this project, testing and treatment uptake was considered to be low using traditional testing methodologies (DBS).<br>**Viral load testing:** Venous bloods<br>**Linkage to care/assessment visit:** NA<br>**Treatment eligibility:** NA. **Treatment initiation:** NA. **Treatment monitoring:** NA<br>**Other features:** These methods led to lengthy turnaround times from the time of the test until the time the result was available. This resulted in patients with active infections being 'lost to follow up' due to prison transfers or release prior to result availability. |
| [54,55]<br>Llerena; 2020<br>Santander, Spain | **Testing approach:** Assisted by the center's "Navigator", systematic screening of HCV is performed by detection of antibodies by Oraquick, of those that are positive, viral load is determined by GeneXpert in capillary blood.<br>**Serological testing:** Antibodies by Oraquick. |

| | |
|---|---|
| People with non-custodial sentences at a "Centre for Social Insertion" | **Viral load testing:** GeneXpert in capillary blood<br>**Linkage to care/assessment visit:** All cases with detectable viral load are evaluated by the hepatology staff by telemedicine and then antiviral treatment is prescribed.<br>**Treatment eligibility:** All eligible. **Treatment initiation:** Same day. Initiate treatment either in the hepatology or infectious units in case of HIV co-infected. **Treatment monitoring:** NA<br>**Other features:** The Navigator figure facilitates continuity for medical care and social assistance of these individuals (accompaniment to the hospital, adherence to treatment etc.). |
| [56]<br>Ustianowski; 2020<br>Manchester, UK<br>Prisoners | **Arm 1: New pathway - March-October 2019**<br>**Testing approach:** Upon arrival, individuals spend first day in a "reception area", includes healthcare check with opt-out offer of rapid HCV test. Repeat testing was also offered for those that initially declined.<br>**Serological testing:** None<br>**Viral load testing:** Opt-out GeneXpert at entry (providing a result in 1-2 hours, whilst still in "reception area")<br>**Linkage to care/assessment visit:** Patients assessed within this first day<br>**Treatment eligibility:** NA. **Treatment initiation:** Medications issued and provided, patient reviewed within 1 week. **Treatment monitoring:** NA<br>**Other features:** Some DBS also performed during this period (507/1037 tests from Mar-Dec were GeneXpert) due to lack of available GeneXpert ports, some reluctance for testing, and lack of staff trained.<br>**Arm 2: Previous pathway: 2018 – historical comparator**<br>**Testing approach:** Upon arrival, individuals spend first day in a "reception area", includes healthcare check but no HCV test. HCV testing was targeted at those deemed most at-risk<br>**Serological testing:** Individual brought back for HCV testing within 2 weeks - DBS test<br>**Viral load testing:** DBS test.<br>**Linkage to care/assessment visit:** Results provided to individual several weeks later and referral made to specialist in-reach hepatitis team who then reviews them.<br>**Treatment eligibility:** NA. **Treatment initiation:** Patient commences treatment following the review. **Treatment monitoring:** NA<br>**Other features:** NA |
| **PLHIV** | |
| [57 31]<br>HEADSTART; 2020<br>Punjab, India<br>PLHIV | **Testing approach:** HIV-positive adults were consecutively enrolled at ART clinics. Non-residents, children or patients already diagnosed with HCV or already initiated on treatment for management of HCV infection were excluded from the study.<br>**Arm 1: ART clinics at district hospitals with GeneXpert Hubs**<br>**Serological testing:** RDT anti-HCV at these district hospitals withpost-test counselling<br>**Viral load testing:** Same visit reflex sample for HCV viral load then GeneXpert. GeneXpert hub uploads viral results to portal same day with result to ART patient given at routine HIV/ART refill visit. Linkage to care: Next visit: initial assessment visit and pre-treatment assessment (including bloods) at medical outpatient department clinic (same hospital). Cirrhosis assessed.<br>**Arm 2: ART clinics at district hospitals**<br>**Serological testing:** RDT at district hospitals with post-test counselling<br>**Viral load testing:** Reflex sample for HCV VL at district hospitals with sample transport to GeneXpert sites at other hospitals, viral load tested using GeneXpert at the other hospitals. GeneXpert hub uploads viral load results to portal same day with result to ART patient given at routine HIV/ART refill visit<br>**Linkage to care/assessment visit:** Next visit: initial assessment visit and pre-treatment assessment (including bloods) at medical outpatient department clinic (same hospital). Cirrhosis assessed.<br>**Treatment eligibility:** NA. **Treatment initiation:** Then treatment initiation visit. Doctor consultation/ultrasound. (3 visits to treatment start). **Treatment monitoring:** Venous sample for SVR12 at district hospitals. Sample transport to 4 GeneXpert hub sites for viral load.<br>**Other features:** NA |
| [58]<br>MSF; 2019<br>Maputo, Mozambique; | **Testing approach:** Routine activity. Enrolment on site. Patients in Maputo often have drug use as a past or present issue.<br>**Serological testing:** RDTs. Non-decentralized initially, now testing with RDTs in drop-in-center. Testing is now also decentralized in a drop-in center for PWUD in Mafalala.<br>**Viral load testing:** GeneXpert for HCV RNA (and HIV VL) – not same day but same site. |

| | |
|---|---|
| PLHIV | **Linkage to care/assessment visit:** Fibroscan, genotyping by DBS. HBV testing. Simplification in 2018 with genotype only in case of cirrhosis.<br>**Treatment eligibility:** NA. **Treatment initiation:** Integration in routine HIV care now. **Treatment monitoring:** NA<br>**Other features:** Telemedicine available. |
| [59]<br>MSF; 2019<br>Mykolaiv, Ukraine<br>PLHIV | **Testing approach:** By the national HIV center (number unclear). Routine activity. The PCC and RND did the initial anti-HCV test with support from the global fund. Data on the numbers tested are unavailable.<br>**Serological testing:** RDTs<br>**Viral load testing:** GeneXpert HCV – not on same day as screening<br>**Linkage to care/assessment visit:** Genotype. APRI, Fibroscan, HBV testing (a simplification model was used by January 2020 where genotype and Fibroscan was dropped)<br>**Treatment eligibility:** HCV care offered to HIV patients followed up in a national HIV center plus PWID but stable on methadone (were referred to the HIV center for treatment). **Treatment initiation:** After the assessment patients were put on a list to be discussed at a weekly round table meeting with all doctors involved (MSF doctors but also local chief doctor): during these sessions, treatment initiation was planned. **Treatment monitoring:** NA<br>**Other features:** Telemedicine available. |
| [59]<br>MSF; 2019<br>Dawei, Myanmar<br>PLHIV | **Testing approach:** Testing was offered to HIV infected patients in 1 clinic<br>**Serological testing:** RDTs – Venous blood was taken by the nurse for other biochemistry tests as well, then RDT test was done by a lab technician<br>**Viral load testing:** Confirmation with GeneXpert for RNA on different day (an appointment was made on the day or after one week)<br>**Linkage to care/assessment visit:** APRI, Fibroscan. Genotype (via hospital in Geneva by DBS). HBV testing. Simplification in 2018 with genotype only for cirrhotics, task-shifting to nurses, lab monitoring decreased.<br>**Treatment eligibility:** HCV care offered to HIV patients followed in an HIV center. **Treatment initiation:** Was done after testing RNA positive and discussion by the team **Treatment monitoring:** NA<br>**Other features:** Telemedicine available. GeneXpert is also used TB diagnosis and Rifampicin resistance detection, and HIV DNA for early infant diagnosis. |

ALT: Alanine aminotransferase. APRI: AST to Platelet Ratio Index. ART: Antiretroviral Therapy. AST: Aspartate aminotransferase. BBV: Bloodborne virus. DAA: Direct acting antiviral. DBS: Dried blood spot. ELISA: Enzyme-linked immunosorbent assay. GP: General practitioner. HBsAg: Hepatitis B surface antigen. HBV: Hepatitis B Virus. HCV: Hepatitis C Virus. MSF: Médecins Sans Frontières. NSP: Needle and syringe programme. OST: Opiate substitution therapy. PCR: Polymerase chain reaction. PLHIV: Persons living with HIV. POC: Point of care. PWID: People who inject drugs. PWUD: People who use drugs. RDT: Rapid diagnostic tests. RNA: Ribonucleic acid. SOC: Standard of care. SVR: Sustained virologic response. TB: Tuberculosis. VL: Viral load.

**Supplementary table 7:** Summary of study characteristics and outcomes across HCV cascade of care for the 64 arms from the 45 studies with at least one PoC assay arm.

| Author | Population | Study Arm | Model of care categorisation* | # visits to from 1st test to treatment | HCV antibody tested ȣ | HCV anitbody +ve | HCV RNA tested | HCV RNA +ve ω | Pre-treatment assessment | Started treatment ǂ ε | SVR12 results available ǂ | SVR12 achieved |
|---|---|---|---|---|---|---|---|---|---|---|---|---|
| Bajis[7] | Homeless | PoC RNA assay | S site, NS visit (RNA on site) | 3 | | | | 45/195 (23%) ω | 27/45 (60%) | 21/27 (78%) [21/45 (47%)] | 13/21 (62%) | 13/13 (100%) |
| LJWG Homeless[8] | Homeless | PoC RNA assay | D site, NS visit (RNA mobile) | 2 | | 119/1054 (11%) | 119/119 (100%) | 72/119 (61%) | | 43/72 (60%) | | |
| Chevaliez[9] | PWID | PoC RNA assay | D site, NS visit (RNA on site) | 3 | | | 89/89 (100%) | 34/89 (38%) | 18/34 (53%) | 16/18 (89%) [16/34 (47%)] | 13/16 (81%) | 13/13 (100%) |
| Lens[10] | PWID | PoC RNA assay | S site, NS visit (RNA on site) | 2 | 386/845 (46%) | 333/386 (86%) | 333/333 (100%) | 212/333 (64%) | | 149/212 (70%) | 98/149 (66%) | 80/98 (82%) |
| Lazarus[11] | PWID | PoC RNA assay | D site, NS visit (RNA mobile) | 4 | | 371/580 (64%) | 125/371 (34%) | 52/125 (42%) | | 39/52 (75%) | | |
| Rogers[12] | PWID | PoC RNA assay | D site, NS visit (RNA on site) | 3 | | | | 30/203 (15%) ω | 23/30 (77%) | 16/23 (70%) [16/30 (53%)] | 13/16 (81%) | 13/13 (100%) |
| Antonini[13] | PWID | PoC RNA assay | D site, NS visit (RNA on site) | 5 | | 19/26 (73%) | 9/19 (47%) | 6/9 (67%) | 2/6 (33%) | | | |
| Remy[14] | PWID | PoC RNA assay | S site, NS visit (RNA mobile) | 2 | | 93/512 (18%) | 93/93 (100%) | 33/93 (35%) | 33/33 (100%) | 33/33 (100%) | | |
| Bajis[15] | PWID | PoC RNA assay | S site, NS visit (RNA on site) | 3 | | | | | 80/189 (42%) | 56/80 (70%) [56/189 (30%)] | | |
| Bajis[15] | PWID | SoC RNA assay | S site, NS visit (RNA off site) | 3 | | | | | 107/165 (65%) | 4/107 (4%) [4/165 (2%)] | | |
| Williams[16] | PWID | PoC RNA assay | S site, NS visit (RNA on site) | 3 | | 150/174 (86%) | 140/150 (93%) | 76/140 (54%) | 48/76 (63%) | | | |
| Valencia[17] | PWID / homeless | PoC RNA assay | D site, S visit (RNA mobile) | 1 | | 197/1577 (12%) | 197/197 (100%) | 71/197 (36%) | | 44/71 (62%) | | |
| Saludes[19] | PWID | PoC RNA assay | S site, NS visit (RNA on site) | NA | | 89/100 (89%) ς | | 63/100 (63%) ς | | | | |
| Saludes[19] | PWID | SoC RNA assay | S site, NS visit (RNA off site) | NA | | | | | | | | |
| Schurch[20] | PWID | PoC RNA assay | S site, NS visit (RNA on site) | 3 | 35/41 (85%) | 14/35 (40%) | | 7/14 (50%) | | 5/7 (71%) | | 5/5 (100%) |
| Schurch[20] | PWID | SoC RNA assay | S site, NS visit (RNA off site) | 4 | 290/291 (100%) | 142/290 (49%) | | 94/142 (66%) | | 52/94 (55%) | | 48/52 (92%) |
| Martel-Laferriere[21] | PWID | PoC RNA assay | D site, NS visit (RNA on site) | 3 | | | | 94/103 (91%) | | 51/64 (80%) τ | | |
| Martel-Laferriere[21] | PWID | SoC RNA assay | D site, NS visit (RNA off site) | 4 | | | | 49/76 (64%) | | 21/32 (66%) [21/49 (43%)] τ | | |

| | | | | | | | | | | | |
|---|---|---|---|---|---|---|---|---|---|---|---|
| Feld[23] | PWID | PoC RNA assay | S site, NS visit (RNA on site) | 3 | | | 47/114 (41%) ω | 25/49 (51%) σ | 9/25 (36%) [9/47 (19%)] | | |
| Thingnes[24] | PWID | PoC RNA assay | S site, NS visit (RNA mobile) | 2 | 25/46 (54%) | 8/25 (32%) | 3/8 (38%) | | 3/3 (100%) | | |
| Stone[26] | PWID | PoC RNA assay | S site, S visit (RNA on site) | 1 | | | 9/27 (33%) ω | | 9/9 (100%) | 8/9 (89%) | 8/8 (100%) |
| MSF Mafalala (Gutierrez) [27] | PWID | PoC RNA assay | D site, NS visit (RNA on site) | NA | | | 38/155 (25%) | 28/38 (74%) | | | |
| Butsashvili[28] | PWID | PoC RNA assay | S site, NS visit (RNA on site) | 3 | | | 209/255 (82%) | | | | |
| Aung[29] | PWID | PoC RNA assay | S site, NS visit (RNA on site) | 2 | | | | | 311/311 (100%) | 290/311 (93%) | 223/290 (77%) |
| Aung[29] | PWID | PoC RNA assay | S site, NS visit (RNA off site) | 2 | | | | | 83/83 (100%) | 75/83 (90%) | 68/75 (91%) |
| Aung[29] | PWID | PoC RNA assay | S site, NS visit (RNA off site) | 2 | | | | | 66/68 (97%) | 64/66 (97%) | 61/64 (95%) |
| HEADSTART Manipur[30] | PWID | PoC RNA assay | S site, NS visit (RNA on site) | 2 | 1532/3273 (47%) | 1396/1532 (91%) | 1192/1396 (85%) | | 975/1192 (82%) | 746/975 (77%) | 630/746 (84%) |
| HEADSTART Manipur[30] | PWID | PoC RNA assay | D site, NS visit (RNA on site) | 3 | 527/1030 (51%) | 411/527 (78%) | 342/411 (83%) | | 300/342 (88%) | 254/300 (85%) | 232/254 (91%) |
| HEADSTART Manipur[30] | PWID | PoC RNA assay | D site, NS visit (RNA on site) | 3 | 1562/3593 (43%) | 1084/1562 (69%) | 943/1084 (87%) | | 786/943 (83%) | 623/786 (79%) | 516/623 (83%) |
| HEADSTART Georgia (Japaridze) [31] | PWID | PoC RNA assay | D site, NS visit (RNA on site) | 3 | | | 514/620 (83%) | | 432/514 (84%) | 308/432 (71%) | 299/308 (97%) |
| HEADSTART Georgia (Japaridze) [31] | PWID | SoC RNA assay | D site, NS visit (RNA off site) | 3 | | | 400/485 (82%) | | 318/400 (80%) | 233/318 (73%) | 231/233 (99%) |
| HEADSTART Georgia (Japaridze) [31] | PWID | SoC RNA assay | D site, NS visit (RNA off site) | 4 | | | 422/516 (82%) | | 373/422 (88%) | 256/373 (69%) | 255/256 (100%) |
| HEADSTART Myanmar (Draper) [33] | PWID | PoC RNA assay | S site, NS visit (RNA on site) | 2 | 606/633 (96%) | 606/606 (100%) | 535/606 (88%) | | 488/535 (91%) | 457/488 (94%) | 421/457 (92%) |
| LJWG NSPs[35] | PWID | SoC RNA assay | D site, NS visit (RNA off site) | 3 | 95/178 (53%) | 23/85 (27%) | 18/23 (78%) | | 16/18 (89%) | | |
| LJWG NSPs[36] | PWID | PoC RNA assay | D site, NS visit (RNA on site) | 2 | | 176/308 (57%) | 66/176 (38%) ω | 21/60 (35%) | 18/21 (86%) [18/66 (27%)] | 4/18 (22%) | 4/4 (100%) |
| SOS hepatites[37] | PWID | PoC RNA assay | S site, S visit (RNA mobile) | 1 | 15/108 (14%) | 12/15 (80%) | 8/12 (67%) | | | | |
| Morris[38] | PWID / homeless | PoC RNA assay | D site, NS visit (RNA on site) | 3 | 71/141 (50%) | 71/71 (100%) | 42/71 (59%) | | 41/42 (98%) | | |
| Wansom[39] | PWID | PoC RNA assay | S site, NS visit (RNA on site) | 2 | 809/1188 (68%) | 809/809 (100%) | 667/809 (82%) | | 573/667 (86%) | 353/573 (62%) | 326/353 (92%) |

| | | | | | | | | | | | | |
|---|---|---|---|---|---|---|---|---|---|---|---|---|
| Sonderup[40] | PWID | PoC RNA assay | S site, S visit (RNA on site) | 1 | | | 139/139 (100%) | 94/139 (68%) | | 86/94 (91%) | 77/86 (90%) | 64/77 (83%) |
| Agwuocha[41] | Gen-pop | PoC RNA assay | D site, NS visit (RNA off site) | 4 | | | 240/612 (39%) | 132/240 (55%) | | | | |
| MSF Uttar Pradesh[42] | Gen-pop | PoC RNA assay | S site, NS visit (RNA on site) | 5 | | 9340/11840 (79%) | | | | 3470/3628 (96%) | 3284/3470 (95%) | 3284/3284 (100%) |
| MSF Phnom Penh (Walker) [43] | Mixed | PoC RNA assay | S site, NS visit (RNA on site) | 3 | | | | 1882/2578 (73%) | | 1391/1882 (74%) | 739/1391 (53%) | 719/739 (97%) |
| MSF Phnom Penh (Walker) [43] | Mixed | SoC RNA assay | S site, NS visit (RNA off site) | 9 | | | | 3445/4969 (69%) | | 3142/3445 (91%) | 2110/3142 (67%) | 2051/2110 (97%) |
| MSF Karachi (Khalid) [44] | Gen-pop / high-risk | PoC RNA assay | S site, NS visit (RNA on site) | 2 | | 752/1971 (38%) | 752/752 (100%) | | | 22/135 (16%) $\gamma$ | | |
| MSF Karachi (Khalid) [44] | Gen-pop / high-risk | SoC RNA assay | S site, NS visit (RNA off site) | 5 | | 1118/2958 (38%) | 1038/1118 (93%) | 643/1038 (62%) | 444/643 (69%) | 163/444 (37%) [163/643 (25%)] | | |
| Qureshi[45] | Gen-pop | PoC RNA assay | S site, NS visit (RNA on site) | NA | | | | | | 8260/10035 (82%) | 2223/8260 (27%) | 2215/2223 (100%) |
| Qureshi[46] | Gen-pop / high-risk | PoC RNA assay | S site, NS visit (RNA on site) | 2 | | 192/5209 (4%) | 177/192 (92%) | 136/177 (77%) | 126/136 (93%) | 126/126 (100%) [126/136 (93%)] | 18/126 (14%) | 18/18 (100%) |
| Hamid[47] | Gen-pop / high-risk | PoC RNA assay | S site, NS visit (RNA off site) | 2 | | 1514/8621 (18%) | 950/950 (100%) | 637/950 (67%) | | 452/637 (71%) | | |
| Hamid[48] | Gen-pop / high-risk | SoC RNA assay | S site, NS visit (RNA off site) | 2 | | 632/8385 (8%) | 632/632 (100%) | 399/632 (63%) | | 330/399 (83%) | | |
| Shiha[50] | Gen-pop | PoC RNA assay | S site, S visit (RNA on site) | 1 | | 125/475 (26%) | 56/56 (100%) $\upsilon$ | 43/56 (77%) | 40/43 (93%) | 40/40 (100%) [40/43 (93%)] | | |
| Shiha[50] | Gen-pop | PoC RNA assay | S site, S visit (RNA on site) | 1 | | 157/3188 (5%) | 76/76 (100%) $\upsilon$ | 38/76 (50%) | 38/38 (100%) | 38/38 (100%) [38/38 (100%)] | | |
| Zhang (MSF)[51] | Gen-pop | PoC RNA assay | D site, NS visit (RNA off site) | 2 | | 778/10425 (7%) | 762/778 (98%) | 540/762 (71%) | 533/540 (99%) | 530/533 (99%) [530/540 (98%)] | 466/530 (88%) | 459/466 (98%) |
| Mohamed[52] | Prisoners | PoC RNA assay | S site, NS visit (RNA on site) | 3 | 162/181 (90%) | 28/162 (17%) | 24/28 (86%) | 20/24 (83%) | | 17/20 (85%) | | |
| Mohamed[52] | Prisoners | SoC RNA assay | S site, NS visit (RNA off site) | 4 | 2442/5239 (47%) | 91/2442 (4%) | 83/91 (91%) | 62/83 (75%) | 37/62 (60%) | 13/37 (35%) [13/62 (21%)] | 8/13 (62%) | 8/8 (100%) |
| Davies[53] | Prisoners | PoC RNA assay | S site, NS visit (RNA on site) | 2 | 835/1253 (67%) | 93/835 (11%) | 97/115 (84%) | 33/97 (34%) | | 32/33 (97%) | | |
| Davies[53] | Prisoners | SoC RNA assay | S site, NS visit (RNA off site) | 3 | 110/826 (13%) | 18/110 (16%) | 17/18 (94%) | 11/17 (65%) | | 4/11 (36%) | | |
| Llerena[54] | Prisoners | PoC RNA assay | S site, S visit (RNA on site) | 1 | 388/425 (91%) | 31/388 (8%) | 31/31 (100%) | 13/31 (42%) | | 13/13 (100%) | 8/13 (62%) | 8/8 (100%) |
| Ustianowski[56] | Prisoners | PoC RNA assay | S site, NS visit (RNA on site) | 2 | | | | 60/507 (12%) ω | | 38/60 (63%) | | |
| Ustianowski[56] | Prisoners | SoC RNA assay | S site, NS visit (RNA off site) | 4 | | | 806/1582 (51%) | 112/806 (14%) ω | | 21/112 (19%) | | |
| HEADSTART Punjab (Markby) [31] | PLHIV | PoC RNA assay | S site, NS visit (RNA on site) | 3 | | 883/4050 (22%) | 870/883 (99%) | 677/870 (78%) | | 359/677 (53%) | 85/359 (24%) | 62/85 (73%) |

| | | | | | | | | | | | |
|---|---|---|---|---|---|---|---|---|---|---|---|
| HEADSTART Punjab (Markby) [31] | PLHIV | PoC RNA assay | S site, NS visit (RNA off site) | 3 | 4258/20691 (21%) | 4211/4258 (99%) | 3430/4211 (81%) | | 1835/3430 (53%) | 574/1835 (31%) | 472/574 (82%) |
| MSF Maputo (Nguyen) [58] | PLHIV | PoC RNA assay | S site, NS visit (RNA on site) | 4 | 49/1398 (4%) | 49/49 (100%) | 42/49 (86%) | | 25/42 (60%) | | |
| MSF Mykolaiv[59] | PLHIV | PoC RNA assay | D site, NS visit (RNA on site) | 4 | | | | | 1168/1289 (91%) [1168/1289 (91%)] | 968/1168 (83%) | 912/968 (94%) |
| MSF Dawei (Nguyen) [59] | PLHIV | PoC RNA assay | S site, NS visit (RNA on site) | 4 | 402/4559 (9%) | 344/402 (86%) | 301/344 (88%) | | 265/301 (88%) [265/301 (88%)] | 250/265 (94%) | 243/250 (97%) |

*Model of care categorisation relates to whether testing and treatment took place at the same or different site, and on the same or a different day.

S site: Same site. D site: Different site. S visit: Same visit. NS visit: Not same visit.

NA: Not available. SVR12: Sustained virological response 12 weeks after treatment. PoC: Point of care. SoC: Standard of care. MSF: Medicins Sans Frontiers. LJWG: London Joint Working Group on Substance Use and Hepatitis C. PWID: people who inject drugs. PLHIV: Persons living with HIV.

ȣ The denominator for HCV antibody tested was the population entering prison for 5 arms (from 3 studies: Davies, Mohamed, Llerena), the population in a harm reduction cohort for 2 arms (from 1 study: Schurch), and the population presented at a harm reduction center for 1 arm (1 study: Lens).

ω Some low RNA percentages were due to the model starting at RNA testing and some were recruiting a population already previously determined to be anti-HCV+. All studies where <50% of anti-HCV+ patients were RNA+ were among PWID or prisoners.

ǂ Some studies were ongoing when results were reported, particularly affecting the percentages starting treatment and assessed for SVR.

$\gamma$ RNA results not recorded in electronic database for 617 patients

$\varepsilon$ For started treatment the numbers given not in the square brackets are those accounting for pre-treatment assessment as the denominator, which is available for some studies, whilst the numbers in the square brackets use the people RNA-positive as the denominator and are used in the quantitative analyses

$\upsilon$ Excluded those previously treated

$\varsigma$ All study participants were tested for antibodies and RNA at the same time

τ Denominator affected by treatment eligibility criteria

σ 2 additional RNA+ results at follow-up visit where baseline results were invalid

**Supplementary table 8:** Median time in days between key steps in the HCV cascade of care for each of the 27 studies with time data available, stratified by model of care arm.

| Study arm | Population category | Year | Model of care* | Ab test to RNA test | RNA sample collection to test | RNA test to results made available | RNA test to treatment start | Ab test to treatment start |
|---|---|---|---|---|---|---|---|---|
| Bajis [7] | PWID/Homeless | 2019 | PoC assay on site: S site, NS visit | . | . | . | 98 (Range: 20-641) | . |
| Lens [10] | PWID/Homeless | 2020 | PoC assay on site: S site, NS visit | 0 | . | . | . | . |
| Antonini [13] | PWID/Homeless | 2018 | PoC assay on site: D site, NS visit | . | . | 0 | . | . |
| Remy [14] | PWID/Homeless | 2019 | PoC assay mobile: S site, NS visit | . | . | . | 1 | . |
| Valencia [17] | PWID/Homeless | 2020 | PoC assay mobile: D site, S visit | 0 | 0 | 0 | 0 | 0 |
| Saludes (C1) [19] | PWID/Homeless | 2020 | PoC assay on site: S site, NS visit | . | . | 1 (Range: 1-8) | . | . |
| Saludes (C2) [19] | PWID/Homeless | 2020 | Lab assay off site: S site, NS visit | . | . | 7 (IQR: 5-11) | . | . |
| Martel-Laferriere (C1) [21] | PWID/Homeless | 2019 | PoC assay on site: D site, NS visit | . | . | . | 70 (IQR: 52-106) | . |
| Martel-Laferriere (C2) [21] | PWID/Homeless | 2019 | Lab assay off site: D site, NS visit | . | . | . | 266 (IQR: 150-449) | . |
| Feld [23] | PWID/Homeless | 2019 | PoC assay on site: S site, NS visit | . | . | 0.04 | . | . |
| Stone [26] | PWID/Homeless | 2019 | PoC assay on site: S site, S visit | . | 0 | 0 | 0 | . |
| Aung (C1) [29] | PWID/Homeless | 2019 | PoC assay on site: S site, NS visit | . | . | . | 14 (IQR: 7-30) | . |
| Aung (C2) [29] | PWID/Homeless | 2019 | Lab PoC assay off site: S site, NS visit | . | . | . | 7 (IQR: 5-11) | . |
| Aung (C3) [29] | PWID/Homeless | 2019 | Lab PoC assay off site: S site, NS visit | . | . | . | 7 (IQR: 4-16) | . |
| HEADSTART Manipur (Ramachandran) (C1) [30] | PWID/Homeless | 2019 | PoC assay on site: S site, NS visit | 0 (IQR: 0-1) | . | . | 13 (IQR: 6-27) | 14 (IQR: 7-30) |
| HEADSTART Manipur (Ramachandran) (C2) [30] | PWID/Homeless | 2019 | PoC assay on site: D site, NS visit | 0 (IQR: 0-3) | . | . | 13.5 (IQR: 6-30) | 17 (IQR: 8-49.25) |
| HEADSTART Manipur (Ramachandran) (C3) [30] | PWID/Homeless | 2019 | PoC assay on site: D site, NS visit | 5 (IQR: 1-15) | . | . | 10 (IQR: 5-19) | 18.5 (IQR: 10-40) |
| HEADSTART Georgia (Japaridze) (C1) [31] | PWID/Homeless | 2020 | PoC assay on site: D site, NS visit | 0 (IQR: 0-0) | 0.07 (IQR: 0.07-0.08) | 0.01 (IQR: 0.01-0.02) | 57 (IQR: 37-87) | 57 (IQR: 39-87) |
| HEADSTART Georgia (Japaridze) (C2) [31] | PWID/Homeless | 2020 | Lab assay off site: D site, NS visit | 0 (IQR: 0-0) | 5.9 (IQR: 3.1-8.0) | 8.9 (IQR: 6.0-15.0) | 31 (IQR: 23-61) | 50 (IQR: 38-80) |
| HEADSTART Georgia (Japaridze) (C3) [31] | PWID/Homeless | 2020 | Lab assay off site: D site, NS visit | 1 (IQR: 0-4) | 5.1 (IQR: 1.2-7.9) | 6.8 (IQR: 3.9-12.8) | 43 (IQR: 29-68) | 67 (IQR: 45-92) |
| HEADSTART Myanmar (Draper) [33] | PWID/Homeless | 2020 | PoC assay on site: S site, NS visit | 0 (IQR: 0-0) | . | . | 3 (IQR: 2-5) | . |
| SOS hepatites [37] | PWID/Homeless | 2020 | PoC assay mobile: S site, S visit | 0 | . | . | . | . |
| Morris [38] | PWID/Homeless | 2020 | PoC assay on site: D site, NS visit | 0 | . | . | 19 (Range: 8-83) | 19 (Range: 8-83) |
| Wansom [39] | PWID/Homeless | 2021 | PoC assay on site: S site, NS visit | 0 | . | . | . | . |
| Sonderup [40] | PWID/Homeless | 2021 | PoC assay on site: S site, S visit | 0 | 0 | 0 | 0 | 0 |

| | | | | | | | | |
|---|---|---|---|---|---|---|---|---|
| MSF Phnom Penh (Walker) (C1) [43] | Gen-pop/mixed | 2018 | PoC assay on site: S site, NS visit | . | 0 (IQR: 0-0) | 0 (IQR: 0-0) | . | . |
| MSF Phnom Penh (Walker) (C2) [43] | Gen-pop/mixed | 2018 | Lab assay off site: S site, NS visit | . | . | 4 (IQR: 3-5) | . | . |
| MSF Karachi (Khalid) (C1) [44] | Gen-pop/mixed | 2020 | PoC assay on site: S site, NS visit | 2 (IQR: 0-74) | . | . | 68 (IQR: 56-120) | . |
| MSF Karachi (Khalid) (C2) [44] | Gen-pop/mixed | 2020 | Lab assay off site: S site, NS visit | 0 | . | . | 107 (IQR: 64-139) | . |
| Qureshi [46] | Gen-pop/mixed | 2020 | PoC assay on site: S site, NS visit | . | . | 0.08 | . | . |
| Shiha (C1) [50] | Gen-pop/mixed | 2019 | PoC assay on site: S site, S visit | 0 | 0 | 0 | 0 | 0 |
| Shiha (C2) [50] | Gen-pop/mixed | 2019 | PoC assay on site: S site, S visit | 0 | 0 | 0 | 0 | 0 |
| MSF (Zhang) [51] | Gen-pop/mixed | 2021 | Lab PoC assay off site: D site, NS visit | 1 (IQR: 0-10) | | 0 | 5 (IQR: 3-8) | |
| Mohamed (C1) [52] | Prisoners | 2020 | PoC assay on site: S site, NS visit | . | 4 (IQR: 3-7) | 0.04 | . | . |
| Mohamed (C2) [52] | Prisoners | 2020 | Lab assay off site: S site, NS visit | . | 15 (IQR: 12-19) | . | . | . |
| Davies (C1) [53] | Prisoners | 2020 | PoC assay on site: S site, NS visit | 0 | . | . | . | . |
| Llerena [54] | Prisoners | 2020 | PoC assay on site: S site, S visit | 0 | 0 | 0 | 0 | 0 |
| Ustianowski (C1) [56] | Prisoners | 2020 | PoC assay on site: S site, NS visit | . | . | 0.08 | 6 (IQR: 3.75-10.25) | . |
| Ustianowski (C2) [56] | Prisoners | 2020 | Lab assay off site: S site, NS visit | . | . | . | 62 | . |
| HEADSTART Punjab (Markby) (C1) [31] | PLHIV | 2020 | PoC assay on site: S site, NS visit | 0 (IQR: 0-0) | 1 (IQR: 0-1) | . | 45 (IQR: 16-127) | 53 (IQR: 19-133) |
| HEADSTART Punjab (Markby) (C2) [31] | PLHIV | 2020 | Lab PoC assay off site: S site, NS visit | 0 (IQR: 0-0) | 2 (IQR: 1-3) | . | 62 (IQR: 24-153) | 64 (IQR: 27-156) |

IQR: Interquartile range. Ab: Antibody. MSF: Medicins Sans Frontiers. LJWG: London Joint Working Group. PLHIV: Persons living with HIV

*Model of care categorisation relates to the where and when testing and treatment took place.

S site: Same site. D site: Different site. S visit: Same visit. NS visit: Not same visit.

***OUTCOME 1: TURN-AROUND TIMES***

Weighted medians of the median days between HCV cascade steps are shown in table 3.

In the 14 arms (n=5640) that measured time from HCV antibody testing to treatment initiation, the average number of days was 53 (95%CI: 17-64) across the included arms. There were clear differences in the overall pooled time reported between HCV antibody testing and treatment initiation between those with PoC assays compared to lab-based assays and in models of care. Due to rapid diagnostic tests, for most arms, patients received their anti-HCV results almost immediately.

Time between anti-HCV testing and treatment was shorter for the 10 arms with PoC RNA assays on site (n=3070) with median: 18.5 days [95%CI: 14-53]) than with lab-based PoC assays (64 days [95%CI: 64-64]: 1 arm, n=1835), or with lab-based non-PoC RNA assays (67 days [95%CI: 50-67]: 2 arms, n=691). There was only small study arm (n=44) that used a PoC RNA assay in a mobile unit and reported a median of 0 days between HCV antibody testing and treatment start.

Most of the differences in time from anti-HCV testing to treatment initiation were due to differing turnaround times from RNA testing to treatment start. The arms that used PoC RNA assays (with low numbers of patients) had the shortest pooled times (13 days [95%CI: 10-14]: 17 arms, n=4010 for PoC RNA assays on site; 0 days [95%CI: 0-1]: 2 arms, n=77 for PoC RNA assays in mobile units). The longest turnaround times were seen for the lab-based testing arms - 62 days ([95%CI: 5-62]: 4 arms, n=2514) for those with lab-based PoC RNA assays and 43 days ([95%CI: 31-107]: 5 arms, n=896) for those using lab-based high-throughput assays.

There were few differences between other earlier steps in the cascade pathway. No differences were observed in the pooled times between HCV antibody testing and RNA testing. For two other steps, there were modest reductions in turnaround times between RNA sample collection to that sample being tested and between RNA testing and the results being made available to the patient with use of PoC RNA assays. For RNA sample collection to sample testing, the shortest pooled times were for PoC RNA assays on site (0 days [95%CI: 0-1]: 9 arms, n=4421) and PoC RNA assays in mobile units (0 days [95%CI: 0-0]: 1 arm, n=197), and slightly longer times seen with lab-based PoC RNA assays (2 days [95%CI: 2-2]: 1 arm, n=4211) and lab-based high-throughput assays (5.9 days [95%CI: 5.1-15]: 3 arms, n=1134). Similarly, the pooled time between RNA testing and results becoming available was shorter when using PoC RNA assays either on site, with a pooled time of 0 days (95%CI: 0-0.01; 13 arms, n=4938) or in mobile units (0 days [95%CI: 0-0]: 1 arm, n=197), compared to with use of lab-based RNA assays. For lab-based PoC assays and lab-based high-throughput assays these times were 0 days ([95%CI: 0-0]: 1 arm, n=762) and 4 days ([95%CI: 4-8.9]: 4 arms, n=6120), respectively.

*Impact of model of care on turnaround times*

For the pooled times between anti-HCV testing and treatment initiation, the shortest times were seen for models 1a (0 days [95%CI: 0-0): 4 arms, n=177) and 2c (0 days [95%CI: 0-0): 1 arm, n=44), with longer times seen for models 1b (14 days [95%CI: 14-53]: 2 arms, n=1334) and 1d (18.5 days [95%CI: 17-57]: 4 arms, n=1559), and the longest times seen for models 3b (64 days [95%CI: 64-64]: 1 arm, n=1835) and 4d (67 days [95%CI: 50-67]: 2 arms, n=691).

The times between anti-HCV testing and RNA testing were similar across the models of care, with the longest pooled times being seen for models 3d (1 day [95%CI: 1-1]: 1 arm, n=762) and 4d (1 day [95%CI: 0-1]: 2 arms, n=1051), compared with 0 days (95%CI: 0-0) for models 1a, 1b, 2a, 2c, 3b, and 4b, and 0 days (95%CI: 0-5; 4 arms: n=2186) for model 1d.

The longest times between RNA sample collection and testing were seen for models 4b (15 days [95%CI: 15-15]: 1 arm, n=83) and 4d (5.1 days [95%CI: 5.1-5.9]: 2 arms, 1051), followed by model 3b (2 days [95%CI: 2-2]: 1 arm, n=4211). Shorter times (0 days [95%CI: 0-0]) were seen for models 1a and 2c, and models 1b (0 days [95%CI: 0-4]: 3 arms, n=3472) and 1d (0.07 days [95%CI: 0.07-0.07]: 1 arm, n=620).

For the times between RNA testing and the results being made available, the pooled median times and 95%CI were all <1 for models 1a, 1b, 1d, 2c, and 3d, with longer times seen for models 4b (4 days [95%CI: 4-7]: 2 arms, n=5069) and 4d (6.8 days [95%CI: 6.8-8.9]: 2 arms, n=1051).

The pooled times between RNA testing and treatment start were shortest for model 1a and 2c (both 0 days [95%CI: 0-0]), then model 2b (1 day [95%CI: 1-1]: 1 arm, n=33). The median time was 5 days (95%CI: 5-5: 1 arm, n=530) for model 3d, with pooled median times of 13 days (95%CI: 3-45: 7 arms, n=2214) for model 1b and 13.5 days (95%CI: 10-57: 5 arms, n=1610) for model 1d. Model 3b had a pooled median time of 62 days (95%CI: 7-62: 3 arms, n=1984), whilst model 4b had a median time of 107 days (95%CI: 62-107: 2 arms, n=184) and model 4d a time of 43 days (95%CI: 31-266: 3 arms, n=712).

***DIRECT WITHIN-STUDY COMPARISONS OF MEDIAN TURNAROUND TIMES***

Supplementary table 9 allows for direct comparison of median turnaround times for within-study PoC assay arms versus lab-based arms by pooling the differences between arms.

*PWID/homeless populations*

The time from RNA testing to results being made available was lower in the PoC arm (1 day [range: 1-8]) than in the lab-based arm (7 days [interquartile range[34]: 5-11]) in the study by Saludes et al[19]. Meanwhile the time from RNA testing to treatment initiation was shorter in the PoC arm (70 days [IQR: 52-106]) than the lab-based arm (266 [IQR: 150-449]) in the Martel-Laferriere study[21].

In the HEADSTART Georgia study[31], the time from antibody testing to RNA testing was short in each arm, but the time from RNA sample collection to testing was shorter in the PoC arm (0.07 days [IQR: 0.07-0.08]) than in the lab-based arms (5.9 days [IQR: 3.1-8.0] and 5.1 [IQR: 1.2-7.9]). Similarly, the time from RNA testing to the results being made available was shorter in the PoC assay arm (0.01 days [IQR: 0.01-0.02]) than in the lab-based arms (8.9 days [6.0-15.0] and 6.8 [IQR: 3.9-12.8]). However, the time from RNA testing to treatment initiation was longer in the PoC arm (57 days [IQR: 37-87]) than in the lab-based arms (31 days [IQR: 23-61] and 43 [IQR: 29-68]) – each arm had testing and treatment at different sites on different visits. Therefore, despite having a shorter time from antibody testing to the patient receiving their RNA result in the PoC arm, the overall times between antibody testing to treatment initiation were similar in the PoC arm (57 days [IQR: 39-87]) and the lab-based arms (50 days [IQR: 38-80] and 67 [IQR: 45-92]).

*General/mixed populations*

In the MSF Phnom Penh study[43], there was a shorter time from RNA testing to the results being made available in the PoC arm (0 days [IQR: 0-0]) than in the lab-based arm (4 days [IQR: 3-5]), whilst in the MSF Karachi study[44] the time between RNA testing and treatment initiation was shorter in the PoC arm (68 days [IQR: 56-120]) then in the lab-based arm (107 days [IQR: 64-139]).

*Prisoners*

There was a shorter time between RNA sample collection and the sample being tested in the PoC arm (4 days [IQR: 3-7]) than in the lab-based arm (15 days [IQR: 12-19]) of the Mohamed study[52]. In the study by Ustianowki et al[56] there was a shorter time from RNA testing to treatment initiation in the PoC arm (6 days [IQR: 3.75-10.25]) than in the lab-based arm (62 days [IQR unavailable]).

*Pooled analysis*

When pooling the within-study differences in time from antibody test to RNA test between the PoC assay arms and the lab-based arms for all population subgroups, there was little evidence of differences across the 3 studies (0.3 days difference [95%CI: -3.5, 4.2]). The pooled time between RNA sample collection and testing was 5.7 days (95%CI: 2.0, 9.4; 3 studies) shorter for the PoC arms, whilst it was 4.9 (95%CI: 1.8, 8.0; 4 studies) days shorter between RNA testing and results being made available. There was little evidence of within-study differences between RNA testing and treatment start (3.3 days difference [95%CI: -59.9, 66.5]; 5 studies), or for antibody testing to treatment start (-1.8 days difference [95%CI: -109.7, 106.1]).

Pooled analyses were only possible for the PWID subgroup due to the lack of studies in other populations. The results of these analyses were similar to the results for all populations combined.

**Supplementary table 9:** Weighted within-study differences in the median days between HCV cascade of care steps, stratified by population group, for 7 studies with PoC and lab-based RNA assay comparator arms

| | | **Weighted* within-study difference in the median days** between cascade steps (95%CI confidence interval) comparing PoC assay arms vs lab-based assay arms***** | | | | |
|---|---|---|---|---|---|---|
| **Study** | **Population** | **Ab test to RNA test** | **RNA sample collection to test** | **RNA test to results made available** | **RNA test to treatment start** | **Ab test to treatment start** |
| **Pooled** | **ALL** | **0.3 (-3.5, 4.2)** | **-5.7 (-9.4, -2.0)** | **-4.9 (-8.0, -1.8)** | **3.3 (-59.9, 66.5)** | **-1.8 (-109.7, 106.1)** |
| Martel-Laferriere (1d vs 4d) | PWID | | | | -196 | |
| Saludes (1b vs 4b) | PWID | | | -6 | | |
| HEADSTART Georgia (Japaridze) (Arm 1 vs 3) (1d vs 4d) | PWID | -1 | -5.03 | -6.79 | 14 | -10 |
| HEADSTART Georgia (Japaridze) (Arm 1 vs 2) (1d vs 4d) | PWID | 0 | -5.83 | -8.89 | 26 | 7 |
| **Pooled** | **PWID** | **-0.5 (-6.9, 5.8)** | **-5.4 (-10.5, -0.3)** | **-7.7 (-11.1, -4.3)** | **10.2 (-126.0, 146.4)** | **-1.8 (-109.7, 106.1)** |
| MSF Phnom Penh (Walker) (1b vs 4b) | Gen-pop | | | -4 | | |
| MSF Karachi (Khalid) (1b vs 4b) | Gen-pop | 2 | | | -39 | |
| **Pooled** | **Gen-pop** | **2 (NA)** | | **-4 (NA)** | **-39 (NA)** | |
| Ustianowski (1b vs 4b) | Prisoners | | | | -56 | |
| Mohamed (1b vs 4b) | Prisoners | | -11 | | | |
| **Pooled** | **Prisoners** | | **-11 (NA)** | | **-56 (NA)** | |

* Weighted by the number of participants in the step of the cascade for each study

** Information was available in different units from different studies, with some reporting in days and some reporting in hours and minutes. Hours and minutes have been converted to days.

*** Negative numbers indicate faster times in the PoC assay arm

Ab: antibody. PoC: Point of care. NA: Not available

*HCV RNA TESTING UPTAKE (ALL POPULATION GROUPS COMBINED)*

Table 4 presents the pooled estimates of percentages for outcomes at key steps across the HCV cascade of care for all population groups combined, stratified by model of care. Supplementary figures 3a-h show corresponding forest plots of cascade of care outcomes, including $I^2$ statistics for assessment of heterogeneity across studies within each model of care category, which was considerable (>75%) for all outcomes and across all categories. A summary of the outcomes across other cascade of care steps can be found in the supplementary table 10. There was no evidence of small study effects (publication bias) for any cascade of care outcome proportion shown in supplementary table 12.

***Impact of PoC versus lab-based assays***

When all population groups and different models of care (a to d) were combined, there was overall little evidence of difference in uptake of RNA testing according to whether the RNA assay was PoC (Arms 1 and 2) or lab-based (Arms 3 and 4). The percentage of participants receiving RNA testing when a PoC RNA assay was on site (Model 1) was 95% (95%CI: 89%-99%) (22 arms, n=8729), and 84% (95%CI: 43%-100%) (6 arms, n=820) when a PoC assay was in a mobile unit (Model 2), while it was 92% (95%CI: 68%-100%) (4 arms, n=6598) for lab-based PoC assays (Model 3), and 82% (95%CI: 53%-99%) (5 arms, n=3526) when using high-throughput lab-based assays (Model 4). The p-value when comparing PoC RNA assays on site or in mobile units (Arms 1 and 2) versus lab-based high-throughput RNA assays (model 4) was 0.30, and 0.93 when comparing Arms 1 and 2 with lab-based PoC-type RNA assays (model 3).

***Impact of model of care***

For both different PoC (Arms 1 and 2) and lab-based RNA testing, (Arms 3 and 4) there was heterogeneity and clear trends according to the additional model of care categorisations (models a-d) and whether testing and treatment were delivered at the same or different site, and on same or different day. In general, there was higher RNA testing uptake with same-site models a and b, compared to different site models c and d (p-values<0.01).

PoC assay on-site (Arms 1): For the 22 arms where a PoC RNA assay was on site, there was higher RNA testing uptake when testing and treatment were in the same location and on the same visit (model 1a: 100% [95%CI: 99%-100%]: 4 arms, n=302), or in the same location but not on the same visit (model 1b: 97% [95%CI: 93%-99%]: 12 arms, n=5851), compared to if there were in a different location on a different visit (model 1d: 82% [95%CI: 68%-92%]: 6 arms, n=2576) – p-value<0.01.

PoC assay in mobile unit (Arms 2): For 6 arms where a PoC RNA assay was in a mobile unit, data in each further model categorisation were available on only one or two arm and fewer patients. RNA testing uptake was high for (model 2a: 80% [95%CI: 52%-96%]: 1 arm, n=15), (model 2b: 95% [95%CI: 90%-99%]: 2 arms, n=118) and (model 2c: 100% [95%CI: 98%-100%]: 1 arm, n=197), but lower for different site and different day (model 2d: 55% [95%CI: 51%-60%]: 2 arms, n=490) – p-value<0.01.

Lab-based PoC RNA assay (Arms 3): For lab-based PoC RNA assays, there was available data on only two models 3b and 3d , with a higher RNA testing uptake with the same site model 3b: 99% [95%CI: 99%-99%]: 2 arms, n=5208) than for arms (model 3d: 79% [95%CI: 77-81%]: 2 arms, n=1390) where testing and treatment were in different locations – p-value<0.01.

Lab-based non-PoC RNA assay (Arms 4): For lab-based high-throughput RNA assays, there was available data on only two models 4b and 4d, with a higher RNA testing uptake for model 4b: 90% [95%CI: 62%-100%]: 5 arms, n=3441) than when testing and treatment were in a different location

on different visits (model 4d: 27% [95%CI: 18%-38%]: 1 stratum: n=85), although only one small study was included that used a different location (the London Joint Working Group NSP report[35]).

***HCV RNA TESTING AND TREATMENT UPTAKE AMONG POPULATION SUBGROUPS***

Table 4 presents the pooled estimates for the two main outcomes of interest across the HCV cascade of care (RNA testing uptake and treatment uptake) for different population subgroups (PWID/homeless, general/mixed population, prisoners, and PLHIV). However, in many cases there were just one or two studies and low numbers of patients within study arms. As a result, few comparisons had large numbers of patients and comparable models of care arms to allow systematic comparisons. Overall, there was some evidence for greater differences with use of PoC viral load compared to lab-based assays among PWID/homeless for RNA testing uptake and treatment uptake and for treatment uptake among prisoners.

*PWID/homeless populations*

Among PWID/homeless populations there were no arms available for RNA testing uptake in mobile units (Arms 3). However, there was some evidence (p<0.01) of higher RNA testing uptake for models 1 (93% [95%CI: 83%-99%]: 12 arms, n=6154) and 2 (84% [95%CI: 43%-100%]: 6 arms, n=820) vs Arms 4 (27% [95%CI: 18%-38%]), although Arms 4 only contained 1 arm with 85 patients.

For treatment uptake, there was no strong evidence (p=0.36) of increased treatment uptake when comparing Arms 1 (73% [95%CI: 64%-82%]: 19 arms, n=5373) and 2 (81% [95%CI: 60%-97%]: 5 arms, n=231) vs Arm 4 (59% [95%CI: 25%-88%]: 6 arms, n=1148). There was also some evidence (p<0.01) of increased treatment uptake with Arm 3 (99% [95%CI: 97%-100%]) vs Arms 1 and 2, however, there were only 2 arms with Arm 3 (both 3b), containing just 151 patients. Similarly, there was some evidence (p<0.01) of increased treatment uptake for Arm 3 vs Arm 4.

When considering the specific models of care, among arms with PoC RNA assays on site, models 1a and 1b had higher RNA testing uptake (100% [95%CI: 97%-100%]: 1 arm, n=139; and 99% [95%CI: 93%-100%]: 5 arms, n=3430, respectively), than model 1d (82% [95%CI: 68%-92%]: 6 arms, n=2576), p-value<0.01. There was also some evidence (p<0.01) of higher RNA testing uptake for model 2b (95% [95%CI: 90%-99%]: 2 arms, n=118) and model 2c (100% [95%CI: 98%-100%]: 1 arm, n=197) compared to model 2d (55% [95%CI: 51%-60%]: 2 arms, n=490).

For arms with PoC assays on site, higher treatment uptake was seen in the two small model 1a arms (94% [95%CI: 88%-99%]: 2 arms, n=103), than model 1b (71% [95%CI: 54%-85%]: 9 arms, n=3205) or model 1d (70% [95%CI: 58%-81%]: 8 arms, n=2065) – p-value<0.01. For the mobile PoC studies, there was some evidence (p<0.01) of higher treatment uptake for model 2b (100% [95%CI: 99%-100%]: 2 arms, n=36) than model 2c (62% [95%CI: 50%-73%]: 1 arm, n=71) or model 2d (66% [95%CI: 58%-74%]: 2 arms, n=124). Among the arms with lab-based high throughput RNA assays, model 4b had lower treatment uptake (16% [95%CI: 11%-20%]: 2 arms, n=259) than model 4d (77% [95%CI: 62%-89%]: 4 arms, n=889), although this was driven by restrictive DAA reimbursement rules at the time of the study in the Bajis PWID study[15] leading to 2% treatment uptake.

*General/mixed populations*

For general/mixed populations, there was little evidence of differences in RNA testing uptake or treatment uptake for any of the comparisons comparing PoC to lab-based assays (all p-values>0.3). The RNA testing uptake in arms with PoC RNA assays on site was 99% (95%CI: 94%-100%) (4 arms, n=1076), whilst it was 88% (95%CI: 42%-100%) (3 arms, n=2340) for lab-based PoC assays, and 97% (95%CI: 96%-98%) (2 arms, n=1750) for lab-based high throughput RNA assays, p=0.34. Similarly,

treatment uptake was 83% (95%CI: 71%-92%) (7 arms, n=15897) for those with PoC RNA assays on site, and for lab-based PoC assay 87% (95%CI: 85%-89%) (2 arms, n=1177), as compared with 69% (24%-99%) (3 arms, n=4487) with lab-based high throughput RNA assays, p=0.53. There were no studies in PoC assays based in mobile units.

There was evidence of higher treatment uptake (p<0.01) from two small studies of higher treatment uptake for model 1a (98% [95%CI: 92%-100%]: 2 arms, n=81) than model 1b (76% [95%CI: 61%-87%]: 5 arms, n=155816). RNA testing uptake was also higher for model 3b (100% [95%CI: 100%-100%]: 1 arm, n=950) than model 3d (79% [95%CI: 77%-81%]: 2 arms, n=1390) – p-value<0.01. There was higher treatment uptake in the arm from MSF Battambang[51] using model 3d (98% [95%CI: 97%-99%]: 1 arm, n=540) than in the arm from Hamid[47,48] using model 3b (71% [95%CI: 67%-74%]: 1 arm, n=637) – p-value<0.01.

*Prisoners*

RNA testing uptake was similar (p=0.48) in arms with PoC RNA assays on site (92% [95%CI: 77%-100%]: 3 arms, n=174) and in arms with non-PoC RNA assays off site (81% [95%CI: 44%-100%]: 3 arms, n=1691), p=0.48. There was evidence of differences in treatment uptake when PoC RNA assays were located on site (89% [95%CI: 67%-100%]: 4 arms, n=126) compared to use of high throughput lab-based RNA assays were located off site (20% [95%CI: 14%-26%]: 3 arms, n=185) (p<0.01).

Higher RNA testing uptake was seen for model 1a (100% [95%CI: 89%-100%]: 1 arm, n=31) compared with model 1b (85% [95%CI: 78%-90%]: 2 arms, n=143) (p<0.01), although numbers of participants were low. There was no evidence of trends in in treatment uptake among the sub-models for prisoners.

*PLHIV*

For PLHIV there were no lab-based high throughput RNA assay arms available. There was no evidence of differences in RNA testing uptake (p=0.38) seen between arms with PoC RNA assays on site (96% [95%CI: 85%-100%]: 3 arms, n=1334) and with lab-based PoC RNA assays (99% [95%CI: 99%-99%]: 1 arm, n=4258). There was very weak evidence (p=0.09) of differences in treatment uptake in arms with PoC RNA assays on site (75% [95%CI: 50%-93%]: 4 arms, n=2309) and with lab-based PoC RNA assays (53% [95%CI: 52%-55%]: 1 arm, n=3430).

No sub-model comparisons of RNA testing uptake were available for PLHIV.

There was weak evidence (p=0.06) of higher treatment uptake for model 1d (91% [95%CI: 89%-92%]: 1 stratum, n=1289) than model 1b (68% [95%CI: 39%-92%]: 3 arms, n=1020).

**Supplementary table 10:** Pooled estimates (and 95% confidence intervals) for percentages uptake of RNA testing and treatment for use of PoC RNA assays compared to lab-based RNA assays, stratified by model of care categorisation. See next page for table footnotes.

| | | **Estimate (95% confidence interval)<br>[Number of arms] (Combined denominator for number of patients included)** | | | | | | |
|---|---|---|---|---|---|---|---|---|
| **Model of care categorisation*<br>(Model #)** | **Median (range) # visits to treatment start‡** | **Anti-HCV testedȣ** | **Anti-HCV positive** | **HCV RNA tested** | **HCV RNA positiveƚ** | **Started treatment** | **Assessed for SVR12** | **SVR12 obtained** |
| **PoC RNA assay on site (Arm 1)** | **3 (1-5)** | **77% (58-92%)<br>[5] (2745)** | **42% (26-58%)<br>[22] (44654)** | **95% (89-99%)<br>[22] (8729)** | **62% (54-70%)<br>[33] (11858)** | **77% (72-83%)<br>[34] (23705)** | **68% (53-82%)<br>[23] (19447)** | **95% (91-98%)<br>[24] (10895)** |
| Same site, same visit (1a) | 1 (1-1) | 91% (88-94%)<br>[1] (425) | 12% (2-26%)<br>[3] (4051) | 100% (99-100%)<br>[4] (302) | 56% (41-70%)<br>[5] (329) | 97% (92-100%)<br>[5] (197) | 83% (63-97%)<br>[3] (108) | 94% (79-100%)<br>[3] (93) |
| Same site, not same visit (1b) | 3 (2-5) | 73% (54-88%)<br>[4] (2320) | 45% (25-66%)<br>[15] (35813) | 97% (93-99%)<br>[12] (5851) | 66% (55-76%)<br>[18] (8608) | 74% (66-81%)<br>[20] (20154) | 63% (41-83%)<br>[13] (16603) | 94% (89-98%)<br>[14] (2183) |
| Different site, not same visit (1d) | 3 (2-5) | NA<br>[0] (0) | 50% (44-57%)<br>[4] (4790) | 82% (68-92%)<br>[6] (2576) | 60% (40-78%)<br>[10] (2921) | 74% (64-82%)<br>[9] (3354) | 77% (70-83%)<br>[7] (2736) | 96% (90-99%)<br>[7] (8696) |
| Within model 1 category p-values | | 0.01 | <0.01 | <0.01 | 0.52 | <0.01 | 0.39 | 0.89 |
| **PoC RNA assay mobile (Arm 2)** | **2 (1-4)** | **NA<br>[0] (0)** | **27% (12-45%)<br>[6] (3877)** | **84% (43-100%)<br>[6] (820)** | **45% (34-55%)<br>[6] (554)** | **81% (60-97%)<br>[5] (231)** | **NA<br>[0] (0)** | **NA<br>[0] (0)** |
| Same site, same visit (2a) | 1 (1-1) | NA<br>[0] (0) | 14% (8-22%)<br>[1] (108) | 80% (52-96%)<br>[1] (15) | 67% (35-90%)<br>[1] (12) | NA<br>[0] (0) | NA<br>[0] (0) | NA<br>[0] (0) |
| Same site, not same visit (2b) | 2 (2-2) | NA<br>[0] (0) | 20% (17-24%)<br>[2] (558) | 95% (90-99%)<br>[2] (118) | 35% (25-45%)<br>[2] (101) | 100% (99-100%)<br>[2] (36) | NA<br>[0] (0) | NA<br>[0] (0) |
| Different site, same visit (2c) | 1 (1-1) | NA<br>[0] (0) | 12% (11-14%)<br>[1] (1577) | 100% (98-100%)<br>[1] (197) | 36% (29-43%)<br>[1] (197) | 62% (50-73%)<br>[1] (71) | NA<br>[0] (0) | NA<br>[0] (0) |
| Different site, not same visit (2d) | 3 (2-4) | NA<br>[0] (0) | 27% (25-30%)<br>[2] (1634) | 55% (51-60%)<br>[2] (490) | 51% (45-57%)<br>[2] (244) | 66% (58-74%)<br>[2] (124) | NA<br>[0] (0) | NA<br>[0] (0) |
| Within model 2 category p-values | | NA | <0.01 | <0.01 | <0.01 | <0.01 | NA | NA |
| **Lab-based PoC RNA assay (Arm 3)** | **2 (2-4)** | **NA<br>[0] (0)** | **15% (7-24%)<br>[3] (39737)** | **92% (68-100%)<br>[4] (6598)** | **69% (58-79%)<br>[4] (6163)** | **89% (66-100%)<br>[5] (4758)** | **79% (48-98%)<br>[5] (2966)** | **92% (83-98%)<br>[5] (1523)** |
| Same site, not same visit (3b) | 2 (2-3) | NA<br>[0] (0) | 20% (19-20%)<br>[2] (29312) | 99% (99-99%)<br>[2] (5208) | 79% (78-80%)<br>[2] (5161) | 85% (65-98%)<br>[4] (4218) | 77% (42-98%)<br>[4] (2436) | 89% (83-93%)<br>[4] (1057) |
| Different site, not same visit (3d) | 3 (2-4) | NA<br>[0] (0) | 7% (7-8%)<br>[1] (10425) | 79% (77-81%)<br>[2] (1390) | 67% (64-70%)<br>[2] (1002) | 98% (97-99%)<br>[1] (540) | 88% (85-91%)<br>[1] (530) | 98% (97-99%)<br>[1] (466) |
| Within model 3 category p-values | | NA | <0.01 | <0.01 | <0.01 | 0.03 | 0.43 | <0.01 |
| **Lab-based SoC RNA assay (Arm 4)** | **4 (2-9)** | **59% (18-93%)<br>[3] (6356)** | **25% (10-43%)<br>[6] (14363)** | **82% (53-99%)<br>[5] (3526)** | **66% (52-78%)<br>[11] (8787)** | **53% (31-75%)<br>[12] (5820)** | **69% (66-72%)<br>[4] (3846)** | **99% (97-100%)<br>[5] (2659)** |
| Same site, not same visit (4b) | 4 (2-9) | 59% (18-93%)<br>[3] (6356) | 20% (7-39%)<br>[5] (14185) | 90% (62-100%)<br>[5] (3441) | 58% (40-76%)<br>[7] (7687) | 41% (12-73%)<br>[8] (4931) | 68% (66-69%)<br>[2] (3155) | 98% (94-100%)<br>[3] (2170) |
| Different site, not same visit (4d) | 4 (3-4) | NA<br>[0] (0) | 53% (46-61%)<br>[1] (178) | 27% (18-38%)<br>[1] (85) | 79% (72-84%)<br>[4] (1100) | 77% (62-89%)<br>[4] (889) | 71% (67-74%)<br>[2] (691) | 99% (98-100%)<br>[2] (489) |
| Within model 4 category p-values | | NA | <0.01 | <0.01 | 0.03 | 0.05 | 0.06 | 0.02 |
| **Arms 1/2 vs Arm 4 p-values** | | **0.42** | **0.23** | **0.30** | **0.49** | **0.03** | **0.93** | **0.02** |
| **Arms 1/2/3 vs Arm 4 p-values** | | **0.42** | **0.30** | **0.31** | **0.56** | **0.02** | **0.83** | **0.01** |
| **Arms 1/2 vs Arm 3 p-values** | | **NA** | **<0.01** | **0.93** | **0.17** | **0.29** | **0.50** | **0.68** |
| **Arm 3 vs Arm 4 p-values** | | **NA** | **0.26** | **0.48** | **0.65** | **0.03** | **0.49** | **0.05** |

PoC: Point of care. SoC: Standard of care. *Model of care categorisation is based on whether the testing and treatment initiation was done at the same location and same visit. ǂ Some models of care started with RNA testing or people already identified as antibody positive. ȣ The denominator for anti-HCV tested was the population entering prison for 5 arms (from 3 studies) the population in a harm reduction cohort for 2 arms (from 1 study), and the population presented at a harm reduction center for 1 arm (1 study). Ɨ Some low RNA percentages were due to the model starting at RNA testing and some were recruiting a population already previously determined to be anti-HCV+. All studies where <50% of anti-HCV+ patients were RNA+ were among PWID or prisoners.

***DIRECT WITHIN-STUDY COMPARISONS OF CASCADE OF CARE PROPORTIONS***

Supplementary table 10 shows the direct within-study comparisons of uptake of RNA testing and treatment in 11 studies that had within study comparator arms for PoC assays and lab-based assays, with one study (HEADSTART Georgia[31]) that had a PoC assay arm compared with two lab-based assay arms. No studies among PLHIV had comparator PoC versus lab-based assay arms.

*PWID/Homeless populations*

Of the five PWID studies, only one, the London Joint Working Group for NSP report showed a clear higher RNA testing uptake in the PoC assay arm than the lab-based assay arm (57% vs 27%; p<0.01)[35,36] (supplementary table 10). However, treatment uptake was substantially lower in the PoC assay arm (18/66: 27% vs 16/18: 89%; p<0.01), although participant numbers were low and decentralised models were used for both arms. In a second study among PWID[15], treatment uptake was higher in the PoC arm than lab-based arm (30% vs 2%; p<0.01), although DAA reimbursement rules changed between the periods when these arms were implemented. For the three other PWID studies, there was little evidence of differences between the arms due to small study sizes: Schurch (71% vs 55%; p=0.46)[20], Martel-Laferriere (80% vs 43%; p=0.20)[21], HEADSTART Georgia comparing against the 1st non-PoC arm (84% vs 80%, p=0.08) and comparing against the 2nd lab-based arm (84% vs 88%, p<0.01)[31].

*General/mixed populations*

The three studies among general/mixed population groups had very different models of care and findings were not consistent across studies. A lower percentage of participants started treatment in the PoC arm than the lab-based arm in the MSF Cambodia Phnom Penh study[43] (74% vs 91%; p<0.01), although there were different durations of follow-up between the two groups. For the MSF Karachi study[44], a higher percentage of participants in the PoC stratum were RNA tested (100% vs 93%; p<0.01), however, a lower percentage started treatment (16% vs 25%; p=0.02), but the study was still ongoing at the time of analysis. In the Hamid study[47,48], treatment uptake was slightly lower in the lab-based PoC stratum in community screening camps than the lab-based non-PoC stratum but these were models involving active outreach in both arms with teams visiting participants in their home (71% vs 83%; p<0.01), and RNA testing uptake was 100% in both arms (p=0.89).

*Prisoners*

Of the three studies among prisoners, there was evidence of higher treatment uptake for the PoC assay arms than the lab-based assay arms, but similar HCV RNA testing uptake. For the Mohamed study[52], there was higher treatment uptake (85% vs 21%; p<0.01) but similar testing uptake (86% vs 91%; p=0.37). Davies et al[53], saw higher treatment uptake (97% vs 36%; p<0.01) but similar testing uptake (84% vs 94%; p=0.30). Whilst treatment uptake was also higher in the PoC stratum in the Ustianowski study[56] (63% vs 19%; p<0.01). However, in each of these studies, there were additional simplification strategies in the PoC arm.

*META-ANALYSIS OF CASCADE OF CARE PROPORTIONS FROM WITHIN-STUDY COMPARISONS*

Figure 3a shows that when pooling the relative risk of RNA testing uptake for the four studies that have both PoC assay with lab-based assay arms[35,36,44,52,53], there was weak evidence of increased RNA testing uptake for PoC assay arms versus lab-based assay arms: relative risk 1.11 (95%CI: 0.89-1.38).

The pooled relative risk for treatment uptake is shown in figure 3b for 10 studies (one with two comparator arms)[15,20,21,31,35,36,43,44,52,53,56], with evidence of increased treatment uptake among PoC assay arms versus lab-based assay arms: relative risk 1.32 (95%CI: 1.06-1.64).

This pooled analysis stratified by population group is shown in supplementary figures 2a and 2b. For the analysis of RNA testing uptake, there was only one study among both PWID and the general population, both showing higher treatment uptake for the PoC assay arms versus lab-based assay arms, relative risks 2.11 (95%CI: 1.47-3.03) and 1.08 (95%CI: 1.06-1.09), respectively. There was little evidence of a difference between PoC and lab-based assay arms for the two studies among prisoners, relative risk: 0.91 (95%CI: 0.82-1.01). For the pooled analysis of treatment uptake, there was little evidence of difference in uptake between the PoC and lab-based assay arms in the 5 studies among PWID, relative risk: 1.38 (95%CI: 0.70-2.71). There was some evidence of lower treatment uptake in the PoC assay arms in the 2 general-population studies, relative risk: 0.79 (95%CI: 0.67-0.92). However, treatment uptake in the PoC arms of these studies[43,44] will have been affected by patients still making their way through the cascade at the time of censoring. For the three studies among prisoners, there was higher treatment uptake in the PoC arms, relative risk: 3.47 (95%CI: 2.56-4.71).

**Supplementary table 11:** Percentage of patients achieving each step in the cascade for PoC vs non-PoC RNA assay arms, for 12 studies with PoC and lab-based RNA assay comparator arms

| Author | RNA Assay | Model of care categorisation* | Anti-HCV tested | Anti-HCV +ve | HCV RNA tested | HCV RNA +ve | Started treatment | SVR12 results available | SVR12 obtained |
|---|---|---|---|---|---|---|---|---|---|
| **PWID** | | | | | | | | | |
| Bajis [15] | PoC | Model 1b: S site, NS visit (RNA on site) | | | | | 56/189 (30%) | | |
| Bajis [15] | Lab-based SoC | Model 4b: S site, NS visit (Lab-based) | | | | | 4/165 (2%) | | |
| | | **P-value:** | | | | | **<0.01** | | |
| Schurch [20] | PoC | Model 1b: S site, NS visit (RNA on site) | 35/41 (85%) | 14/35 (40%) | | 7/14 (50%) | 5/7 (71%) | | 5/5 (100%) |
| Schurch [20] | Lab-based SoC | Model 4b: S site, NS visit (Lab-based) | 290/291 (100%) | 142/290 (49%) | | 94/142 (66%) | 52/94 (55%) | | 48/52 (92%) |
| | | **P-value:** | **<0.01** | **0.32** | | **0.23** | **0.46** | | **0.70** |
| Martel-Laferriere [21] | PoC | Model 1d: D site, NS visit (RNA on site) | | | | 94/103 (91%) | 51/64 (80%) | | |
| Martel-Laferriere [21] | Lab-based SoC | D site, NS visit (Lab-based) | | | | 49/76 (64%) | 21/49 (43%) | | |
| | | **P-value:** | | | | **<0.01** | **0.20** | | |
| HEADSTART Georgia (Japaridze) [31] | PoC | Model 1d: D site, NS visit (RNA on site) | | | | 514/620 (83%) | 432/514 (84%) | 308/432 (71%) | 299/308 (97%) |
| HEADSTART Georgia (Japaridze) [31] | Lab-based SoC (1) | Model 4d: D site, NS visit (Lab-based) | | | | 400/485 (82%) | 318/400 (80%) | 233/318 (73%) | 231/233 (99%) |
| | | **P-value:** | | | | **0.85** | **0.08** | **0.56** | **0.09** |
| HEADSTART Georgia (Japaridze) [31] | PoC | Model 1d: D site, NS visit (RNA on site) | | | | 514/620 (83%) | 432/514 (84%) | 308/432 (71%) | 299/308 (97%) |
| HEADSTART Georgia (Japaridze) [31] | Lab-based SoC (2) | Model 4d: D site, NS visit (Lab-based) | | | | 422/516 (82%) | 373/422 (88%) | 256/373 (69%) | 255/256 (100%) |
| | | **P-value:** | | | | **0.82** | **0.06** | **0.41** | **0.02** |
| LJWG NSPs [36] | PoC | Model 1d: D site, NS visit (RNA on site) | | | 176/308 (57%) | 66/176 (38%) | 18/66 (27%) | | |
| LJWG NSPs [35] | Lab-based SoC | Model 4d: D site, NS visit (Lab-based) | | | 23/85 (27%) | 18/23 (78%) | 16/18 (89%) | | |
| | | **P-value:** | | | **<0.01** | **<0.01** | **<0.01** | | |
| **General population/mixed** | | | | | | | | | |
| MSF Phnom Penh (Walker) [43] | PoC | Model 1b: S site, NS visit (RNA on site) | | | | 1882/2578 (73%) | 1391/1882 (74%) | 739/1391 (53%) | 719/739 (97%) |
| MSF Phnom Penh (Walker) [43] | Lab-based SoC | Model 4b: S site, NS visit (Lab-based) | | | | 3445/4969 (69%) | 3142/3445 (91%) | 2110/3142 (67%) | 2051/2110 (97%) |

| | | | | | | | | | |
|---|---|---|---|---|---|---|---|---|---|
| | | **P-value:** | | | | **<0.01** | **<0.01** | **<0.01** | **0.94** |
| MSF Karachi (Khalid) [44] | PoC | Model 1b: S site, NS visit (RNA on site) | | 752/1971 (38%) | 752/752 (100%) | | 22/135 (16%) | | |
| MSF Karachi (Khalid) [44] | Lab-based SoC | Model 4b: S site, NS visit (Lab-based) | | 1118/2958 (38%) | 1038/1118 (93%) | | 163/643 (25%) | | |
| | | **P-value:** | | **0.80** | **<0.01** | | **0.02** | | |
| Hamid [47] | Lab-based PoC | Model 3b: S site, NS visit (Lab-based) | | 1514/8621 (18%) | 950/950 (100%) | 637/950 (67%) | 452/637 (71%) | | |
| Hamid [48] | Lab-based SoC | Model 4b: S site, NS visit (Lab-based) | | 632/8385 (8%) | 632/632 (100%) | 399/632 (63%) | 330/399 (83%) | | |
| | | **P-value:** | | **<0.01** | **0.89** | **0.11** | **<0.01** | | |
| **Prisoners** | | | | | | | | | |
| Mohamed [52] | PoC | Model 1b: S site, NS visit (RNA on site) | 162/181 (90%) | 28/162 (17%) | 24/28 (86%) | 20/24 (83%) | 17/20 (85%) | | |
| Mohamed [52] | Lab-based SoC | Model 4b: S site, NS visit (RNA off site) | 2442/5239 (47%) | 91/2442 (4%) | 83/91 (91%) | 62/83 (75%) | 13/62 (21%) | | |
| | | **P-value:** | **<0.01** | **<0.01** | **0.37** | **0.42** | **<0.01** | | |
| Davies [53] | PoC | Model 1b: S site, NS visit (RNA on site) | 835/1253 (67%) | 93/835 (11%) | 97/115 (84%) | 33/97 (34%) | 32/33 (97%) | | |
| Davies [53] | Lab-based SoC | Model 4b: S site, NS visit (Lab-based) | 110/826 (13%) | 18/110 (16%) | 17/18 (94%) | 11/17 (65%) | 4/11 (36%) | | |
| | | **P-value:** | **<0.01** | **0.12** | **0.30** | **0.02** | **<0.01** | | |
| Ustianowski [56] | PoC | Model 1b: S site, NS visit (RNA on site) | | | | 60/507 (12%) | 38/60 (63%) | | |
| Ustianowski [56] | Lab-based SoC | Model 4b: S site, NS visit (Lab-based) | | | | 112/806 (14%) | 21/112 (19%) | | |
| | | **P-value:** | | | | **0.28** | **<0.01** | | |

*Model of care categorisation relates to the where and when testing and treatment took place. S site: Same site. D site: Different site. S visit: Same visit. NS visit: Not same visit.
SVR12: Sustained virological response 12 weeks after treatment. PoC: Point of care. SoC: Standard of care. MSF: Medicins Sans Frontiers. LJWG: London Joint Working Group on Substance Use and Hepatitis C. PWID: people who inject drugs. PLHIV: People living with HIV.
ȣThe denominator for anti-HCV tested was the population entering prison for Davies and Mohamed and the population in a harm reduction cohort for Schurch
ƗSome low RNA percentages were due to the model starting at RNA testing and some were recruiting a population already previously determined to be anti-HCV+. All studies where <50% of anti-HCV+ patients were RNA+ were among PWID or prisoners.
ǂSome studies were ongoing when results were reported, particularly affecting the percentages starting treatment and assessed for SVR.

**Supplementary figure 2a-b:** Meta-analysis of within study comparisons of PoC vs lab assay arms for the relative risks of a) RNA testing uptake and b) treatment uptake, stratified by population type. P-values of 0.000 should be interpreted as <0.001.

a)

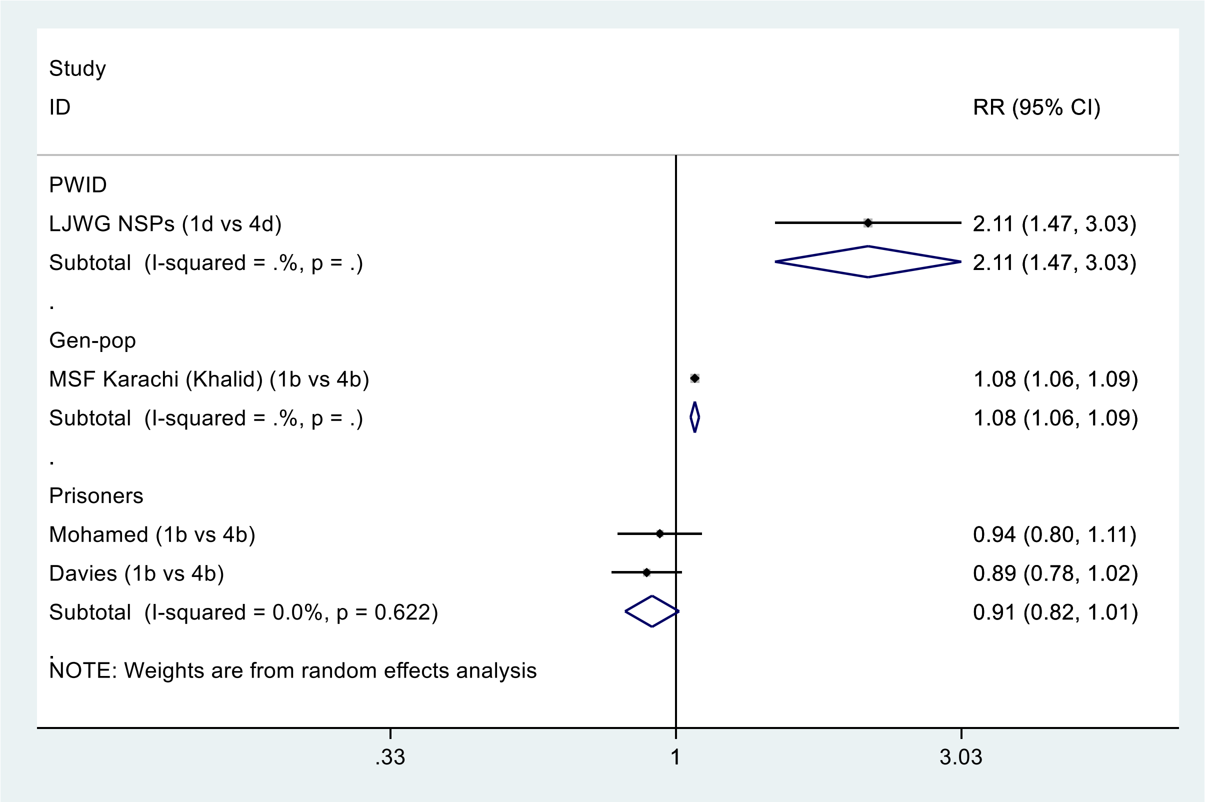


b)

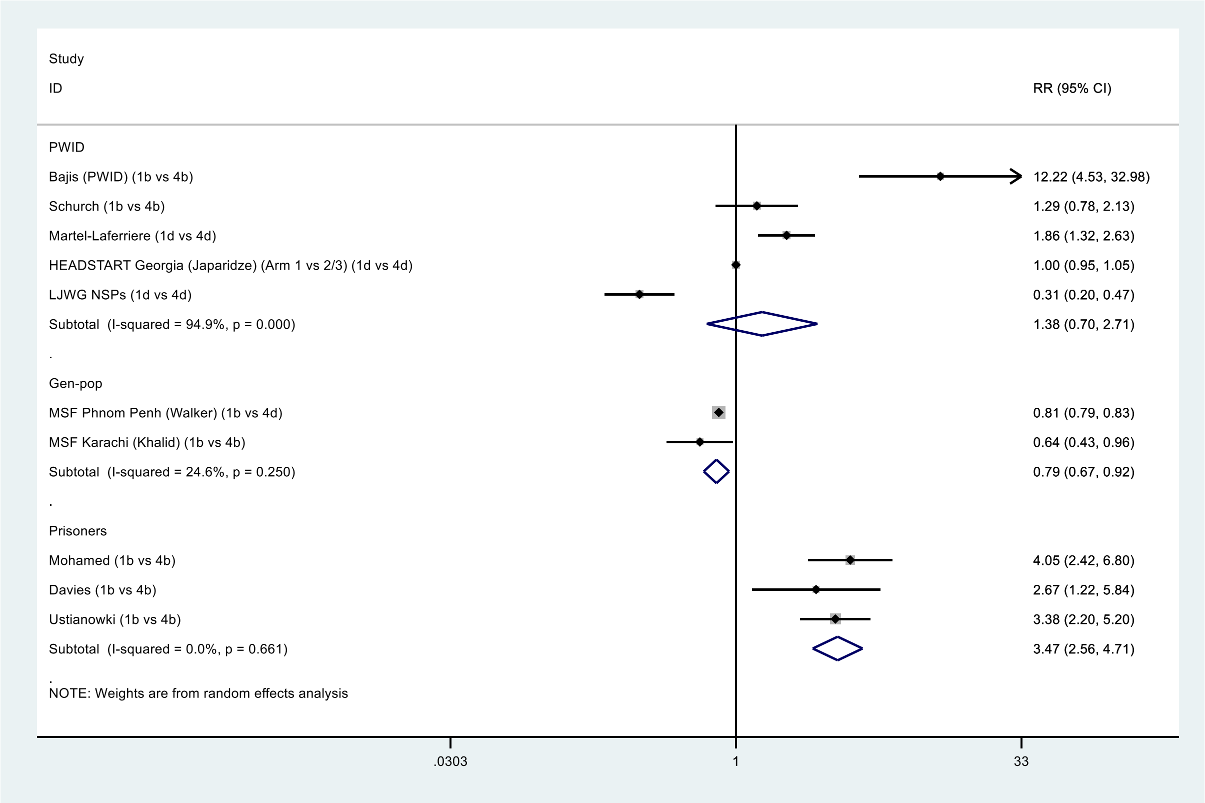

**Supplementary figure 3a-h:** Forest plots of cascade of care outcomes from random-effects meta analyses; ES = proportion. P-values of 0.000 should be interpreted as <0.001.

a)

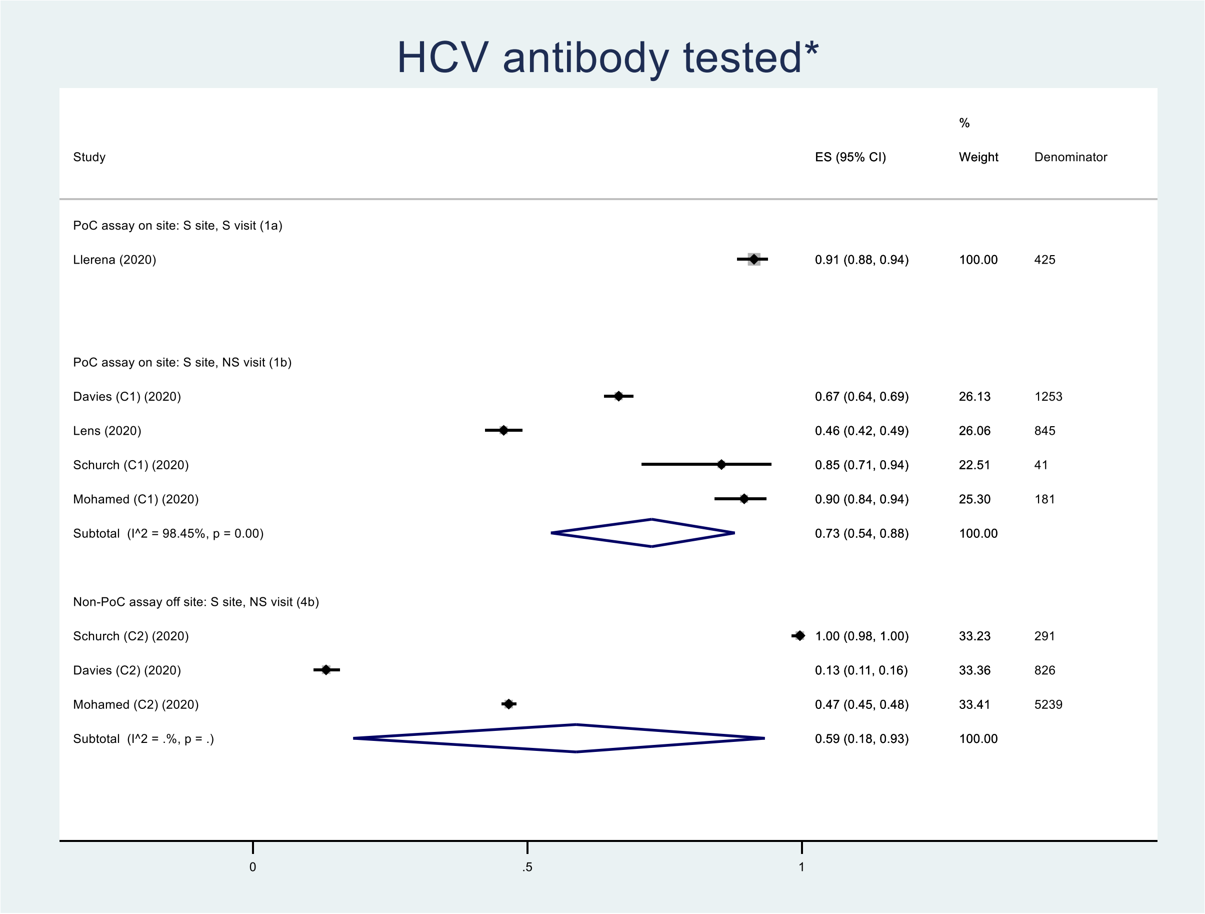


* The denominator for anti-HCV tested was the population entering prison for 5 arms (from 3 studies: Davies, Mohamed, Llerena), the population in a harm reduction cohort for 2 arms (from 1 study: Schurch), and the population presented at a harm reduction center for 1 arm (1 study: Lens).

b)

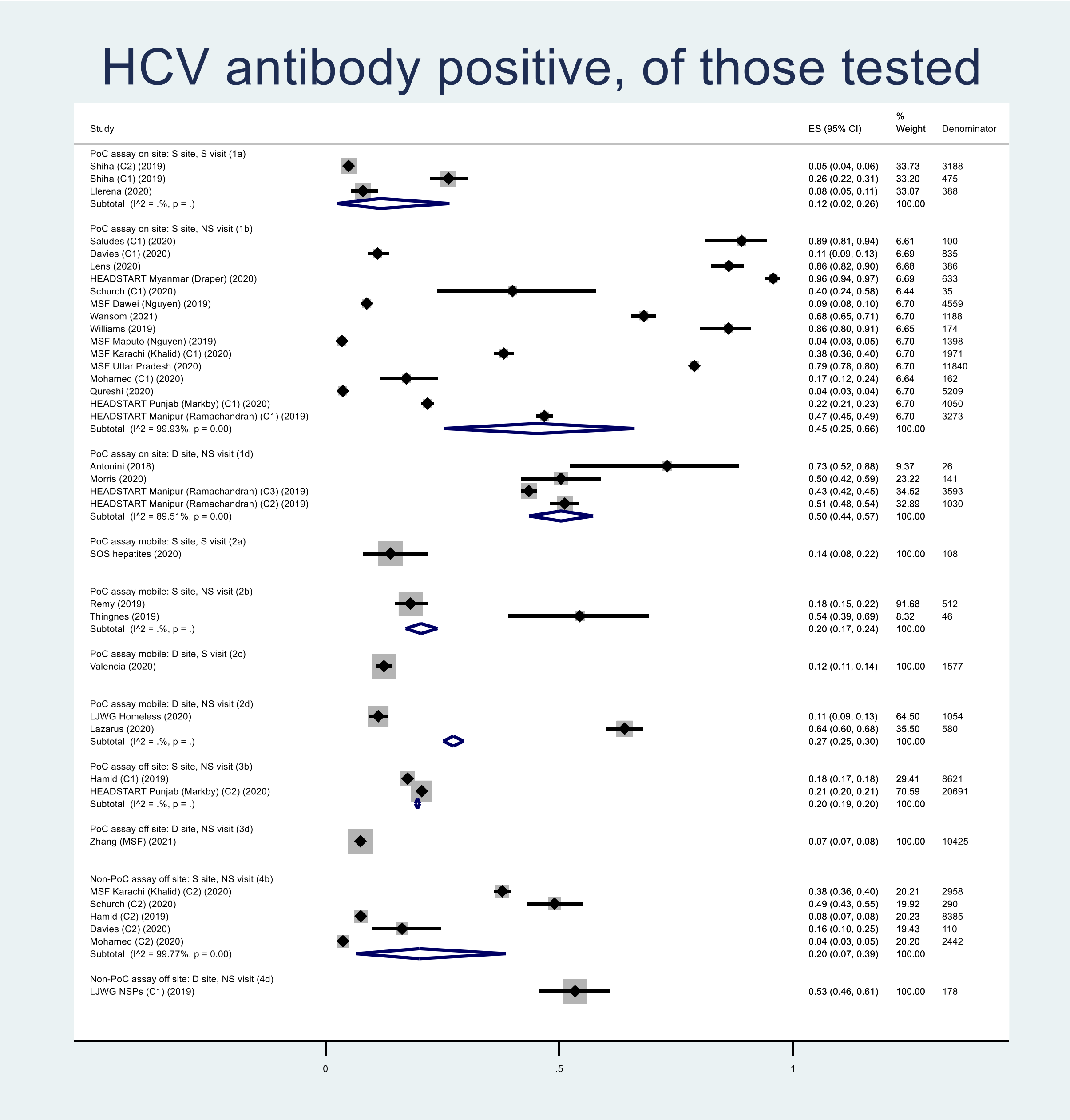
HCV antibody positive, of those tested
Study
ES (95% CI)
% Weight
Denominator
PoC assay on site: S site, S visit (1a)
Shiha (C2) (2019) 0.05 (0.04, 0.06) 33.73 3188
Shiha (C1) (2019) 0.26 (0.22, 0.31) 33.20 475
Llerena (2020) 0.08 (0.05, 0.11) 33.07 388
Subtotal (I^2 = .%, p = .) 0.12 (0.02, 0.26) 100.00
PoC assay on site: S site, NS visit (1b)
Saludes (C1) (2020) 0.89 (0.81, 0.94) 6.61 100
Davies (C1) (2020) 0.11 (0.09, 0.13) 6.69 835
Lens (2020) 0.86 (0.82, 0.90) 6.68 386
HEADSTART Myanmar (Draper) (2020) 0.96 (0.94, 0.97) 6.69 633
Schurch (C1) (2020) 0.40 (0.24, 0.58) 6.44 35
MSF Dawei (Nguyen) (2019) 0.09 (0.08, 0.10) 6.70 4559
Wansom (2021) 0.68 (0.65, 0.71) 6.70 1188
Williams (2019) 0.86 (0.80, 0.91) 6.65 174
MSF Maputo (Nguyen) (2019) 0.04 (0.03, 0.05) 6.70 1398
MSF Karachi (Khalid) (C1) (2020) 0.38 (0.36, 0.40) 6.70 1971
MSF Uttar Pradesh (2020) 0.79 (0.78, 0.80) 6.70 11840
Mohamed (C1) (2020) 0.17 (0.12, 0.24) 6.64 162
Qureshi (2020) 0.04 (0.03, 0.04) 6.70 5209
HEADSTART Punjab (Markby) (C1) (2020) 0.22 (0.21, 0.23) 6.70 4050
HEADSTART Manipur (Ramachandran) (C1) (2019) 0.47 (0.45, 0.49) 6.70 3273
Subtotal (I^2 = 99.93%, p = 0.00) 0.45 (0.25, 0.66) 100.00
PoC assay on site: D site, NS visit (1d)
Antonini (2018) 0.73 (0.52, 0.88) 9.37 26
Morris (2020) 0.50 (0.42, 0.59) 23.22 141
HEADSTART Manipur (Ramachandran) (C3) (2019) 0.43 (0.42, 0.45) 34.52 3593
HEADSTART Manipur (Ramachandran) (C2) (2019) 0.51 (0.48, 0.54) 32.89 1030
Subtotal (I^2 = 89.51%, p = 0.00) 0.50 (0.44, 0.57) 100.00
PoC assay mobile: S site, S visit (2a)
SOS hepatites (2020) 0.14 (0.08, 0.22) 100.00 108
PoC assay mobile: S site, NS visit (2b)
Remy (2019) 0.18 (0.15, 0.22) 91.68 512
Thingnes (2019) 0.54 (0.39, 0.69) 8.32 46
Subtotal (I^2 = .%, p = .) 0.20 (0.17, 0.24) 100.00
PoC assay mobile: D site, S visit (2c)
Valencia (2020) 0.12 (0.11, 0.14) 100.00 1577
PoC assay mobile: D site, NS visit (2d)
LJWG Homeless (2020) 0.11 (0.09, 0.13) 64.50 1054
Lazarus (2020) 0.64 (0.60, 0.68) 35.50 580
Subtotal (I^2 = .%, p = .) 0.27 (0.25, 0.30) 100.00
PoC assay off site: S site, NS visit (3b)
Hamid (C1) (2019) 0.18 (0.17, 0.18) 29.41 8621
HEADSTART Punjab (Markby) (C2) (2020) 0.21 (0.20, 0.21) 70.59 20691
Subtotal (I^2 = .%, p = .) 0.20 (0.19, 0.20) 100.00
PoC assay off site: D site, NS visit (3d)
Zhang (MSF) (2021) 0.07 (0.07, 0.08) 100.00 10425
Non-PoC assay off site: S site, NS visit (4b)
MSF Karachi (Khalid) (C2) (2020) 0.38 (0.36, 0.40) 20.21 2958
Schurch (C2) (2020) 0.49 (0.43, 0.55) 19.92 290
Hamid (C2) (2019) 0.08 (0.07, 0.08) 20.23 8385
Davies (C2) (2020) 0.16 (0.10, 0.25) 19.43 110
Mohamed (C2) (2020) 0.04 (0.03, 0.05) 20.20 2442
Subtotal (I^2 = 99.77%, p = 0.00) 0.20 (0.07, 0.39) 100.00
Non-PoC assay off site: D site, NS visit (4d)
LJWG NSPs (C1) (2019) 0.53 (0.46, 0.61) 100.00 178
0
.5
1

c)

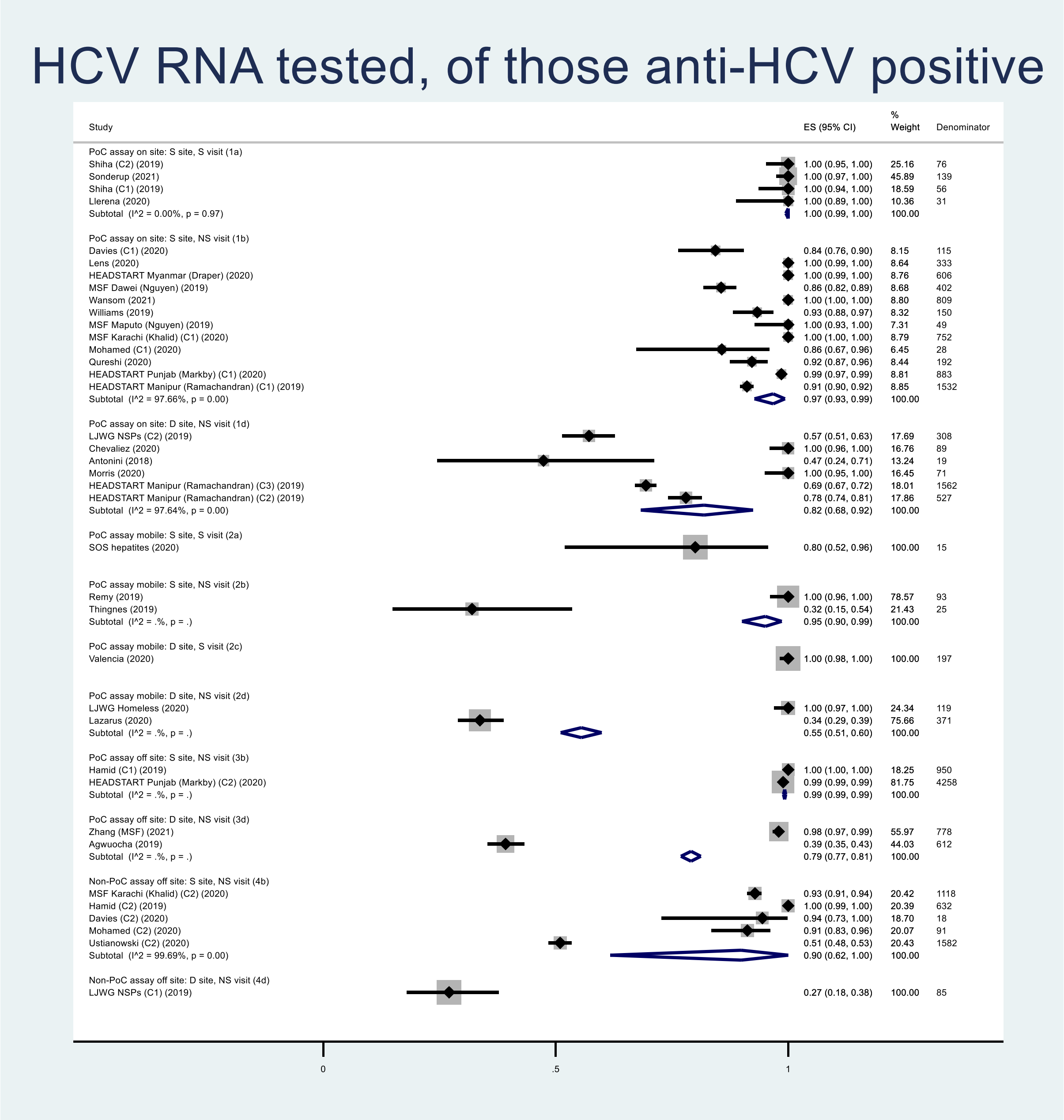
HCV RNA tested, of those anti-HCV positive
Study
ES (95% CI)
% Weight
Denominator
PoC assay on site: S site, S visit (1a)
Shiha (C2) (2019) 1.00 (0.95, 1.00) 25.16 76
Sonderup (2021) 1.00 (0.97, 1.00) 45.89 139
Shiha (C1) (2019) 1.00 (0.94, 1.00) 18.59 56
Llerena (2020) 1.00 (0.89, 1.00) 10.36 31
Subtotal (I^2 = 0.00%, p = 0.97) 1.00 (0.99, 1.00) 100.00
PoC assay on site: S site, NS visit (1b)
Davies (C1) (2020) 0.84 (0.76, 0.90) 8.15 115
Lens (2020) 1.00 (0.99, 1.00) 8.64 333
HEADSTART Myanmar (Draper) (2020) 1.00 (0.99, 1.00) 8.76 606
MSF Dawei (Nguyen) (2019) 0.86 (0.82, 0.89) 8.68 402
Wansom (2021) 1.00 (1.00, 1.00) 8.80 809
Williams (2019) 0.93 (0.88, 0.97) 8.32 150
MSF Maputo (Nguyen) (2019) 1.00 (0.93, 1.00) 7.31 49
MSF Karachi (Khalid) (C1) (2020) 1.00 (1.00, 1.00) 8.79 752
Mohamed (C1) (2020) 0.86 (0.67, 0.96) 6.45 28
Qureshi (2020) 0.92 (0.87, 0.96) 8.44 192
HEADSTART Punjab (Markby) (C1) (2020) 0.99 (0.97, 0.99) 8.81 883
HEADSTART Manipur (Ramachandran) (C1) (2019) 0.91 (0.90, 0.92) 8.85 1532
Subtotal (I^2 = 97.66%, p = 0.00) 0.97 (0.93, 0.99) 100.00
PoC assay on site: D site, NS visit (1d)
LJWG NSPs (C2) (2019) 0.57 (0.51, 0.63) 17.69 308
Chevaliez (2020) 1.00 (0.96, 1.00) 16.76 89
Antonini (2018) 0.47 (0.24, 0.71) 13.24 19
Morris (2020) 1.00 (0.95, 1.00) 16.45 71
HEADSTART Manipur (Ramachandran) (C3) (2019) 0.69 (0.67, 0.72) 18.01 1562
HEADSTART Manipur (Ramachandran) (C2) (2019) 0.78 (0.74, 0.81) 17.86 527
Subtotal (I^2 = 97.64%, p = 0.00) 0.82 (0.68, 0.92) 100.00
PoC assay mobile: S site, S visit (2a)
SOS hepatites (2020) 0.80 (0.52, 0.96) 100.00 15
PoC assay mobile: S site, NS visit (2b)
Remy (2019) 1.00 (0.96, 1.00) 78.57 93
Thingnes (2019) 0.32 (0.15, 0.54) 21.43 25
Subtotal (I^2 = .%, p = .) 0.95 (0.90, 0.99) 100.00
PoC assay mobile: D site, S visit (2c)
Valencia (2020) 1.00 (0.98, 1.00) 100.00 197
PoC assay mobile: D site, NS visit (2d)
LJWG Homeless (2020) 1.00 (0.97, 1.00) 24.34 119
Lazarus (2020) 0.34 (0.29, 0.39) 75.66 371
Subtotal (I^2 = .%, p = .) 0.55 (0.51, 0.60) 100.00
PoC assay off site: S site, NS visit (3b)
Hamid (C1) (2019) 1.00 (1.00, 1.00) 18.25 950
HEADSTART Punjab (Markby) (C2) (2020) 0.99 (0.99, 0.99) 81.75 4258
Subtotal (I^2 = .%, p = .) 0.99 (0.99, 0.99) 100.00
PoC assay off site: D site, NS visit (3d)
Zhang (MSF) (2021) 0.98 (0.97, 0.99) 55.97 778
Agwuocha (2019) 0.39 (0.35, 0.43) 44.03 612
Subtotal (I^2 = .%, p = .) 0.79 (0.77, 0.81) 100.00
Non-PoC assay off site: S site, NS visit (4b)
MSF Karachi (Khalid) (C2) (2020) 0.93 (0.91, 0.94) 20.42 1118
Hamid (C2) (2019) 1.00 (0.99, 1.00) 20.39 632
Davies (C2) (2020) 0.94 (0.73, 1.00) 18.70 18
Mohamed (C2) (2020) 0.91 (0.83, 0.96) 20.07 91
Ustianowski (C2) (2020) 0.51 (0.48, 0.53) 20.43 1582
Subtotal (I^2 = 99.69%, p = 0.00) 0.90 (0.62, 1.00) 100.00
Non-PoC assay off site: D site, NS visit (4d)
LJWG NSPs (C1) (2019) 0.27 (0.18, 0.38) 100.00 85
0
.5
1

d)

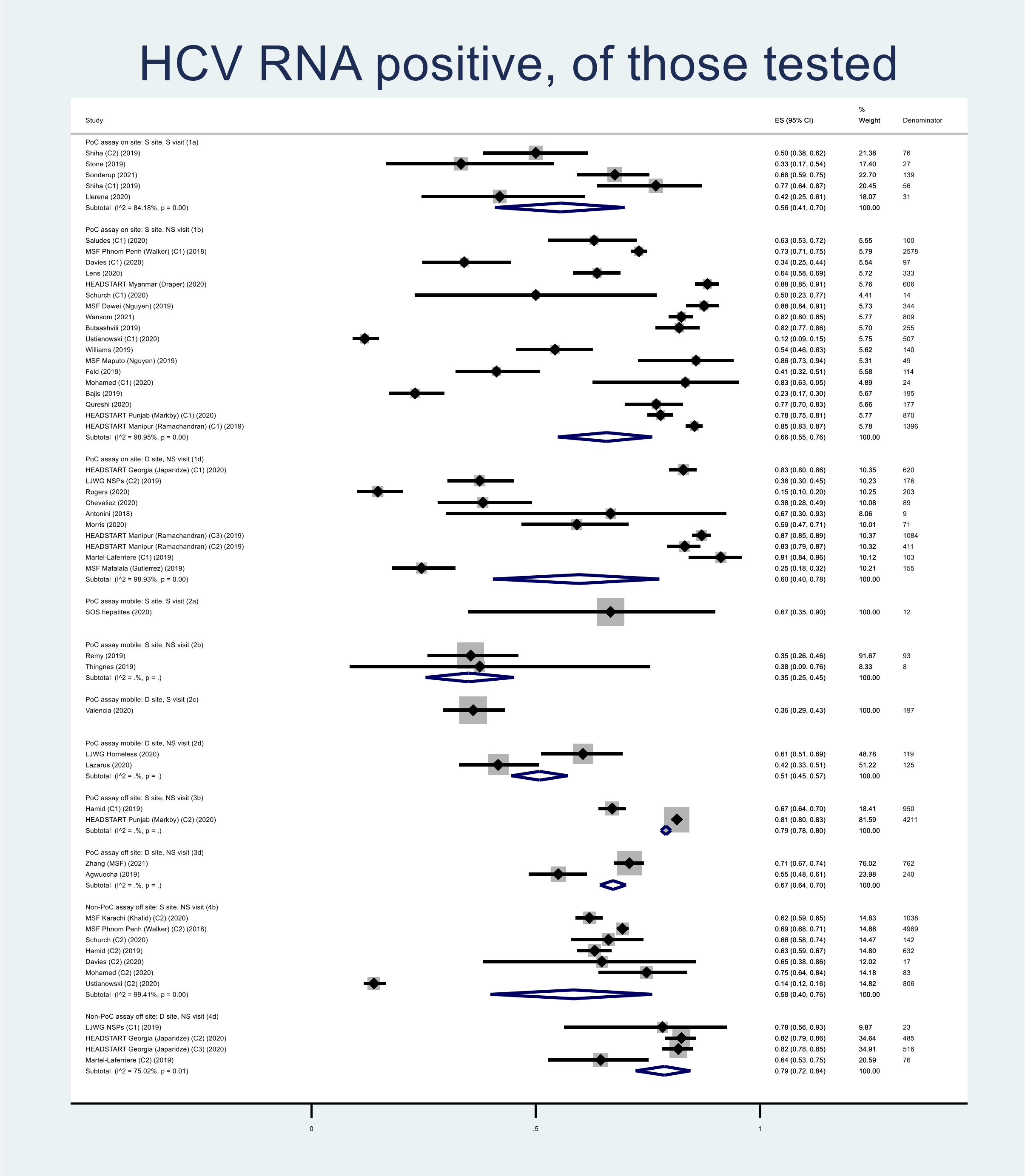
HCV RNA positive, of those tested
Study
ES (95% CI)
% Weight
Denominator
PoC assay on site: S site, S visit (1a)
Shiha (C2) (2019) 0.50 (0.38, 0.62) 21.38 76
Stone (2019) 0.33 (0.17, 0.54) 17.40 27
Sonderup (2021) 0.68 (0.59, 0.75) 22.70 139
Shiha (C1) (2019) 0.77 (0.64, 0.87) 20.45 56
Llerena (2020) 0.42 (0.25, 0.61) 18.07 31
Subtotal (I^2 = 84.18%, p = 0.00) 0.56 (0.41, 0.70) 100.00
PoC assay on site: S site, NS visit (1b)
Saludes (C1) (2020) 0.63 (0.53, 0.72) 5.55 100
MSF Phnom Penh (Walker) (C1) (2018) 0.73 (0.71, 0.75) 5.79 2578
Davies (C1) (2020) 0.34 (0.25, 0.44) 5.54 97
Lens (2020) 0.64 (0.58, 0.69) 5.72 333
HEADSTART Myanmar (Draper) (2020) 0.88 (0.85, 0.91) 5.76 606
Schurch (C1) (2020) 0.50 (0.23, 0.77) 4.41 14
MSF Dawei (Nguyen) (2019) 0.88 (0.84, 0.91) 5.73 344
Wansom (2021) 0.82 (0.80, 0.85) 5.77 809
Butsashvili (2019) 0.82 (0.77, 0.86) 5.70 255
Ustianowski (C1) (2020) 0.12 (0.09, 0.15) 5.75 507
Williams (2019) 0.54 (0.46, 0.63) 5.62 140
MSF Maputo (Nguyen) (2019) 0.86 (0.73, 0.94) 5.31 49
Feld (2019) 0.41 (0.32, 0.51) 5.58 114
Mohamed (C1) (2020) 0.83 (0.63, 0.95) 4.89 24
Bajis (2019) 0.23 (0.17, 0.30) 5.67 195
Qureshi (2020) 0.77 (0.70, 0.83) 5.66 177
HEADSTART Punjab (Markby) (C1) (2020) 0.78 (0.75, 0.81) 5.77 870
HEADSTART Manipur (Ramachandran) (C1) (2019) 0.85 (0.83, 0.87) 5.78 1396
Subtotal (I^2 = 98.95%, p = 0.00) 0.66 (0.55, 0.76) 100.00
PoC assay on site: D site, NS visit (1d)
HEADSTART Georgia (Japaridze) (C1) (2020) 0.83 (0.80, 0.86) 10.35 620
LJWG NSPs (C2) (2019) 0.38 (0.30, 0.45) 10.23 176
Rogers (2020) 0.15 (0.10, 0.20) 10.25 203
Chevaliez (2020) 0.38 (0.28, 0.49) 10.08 89
Antonini (2018) 0.67 (0.30, 0.93) 8.06 9
Morris (2020) 0.59 (0.47, 0.71) 10.01 71
HEADSTART Manipur (Ramachandran) (C3) (2019) 0.87 (0.85, 0.89) 10.37 1084
HEADSTART Manipur (Ramachandran) (C2) (2019) 0.83 (0.79, 0.87) 10.32 411
Martel-Laferriere (C1) (2019) 0.91 (0.84, 0.96) 10.12 103
MSF Mafalala (Gutierrez) (2019) 0.25 (0.18, 0.32) 10.21 155
Subtotal (I^2 = 98.93%, p = 0.00) 0.60 (0.40, 0.78) 100.00
PoC assay mobile: S site, S visit (2a)
SOS hepatites (2020) 0.67 (0.35, 0.90) 100.00 12
PoC assay mobile: S site, NS visit (2b)
Remy (2019) 0.35 (0.26, 0.46) 91.67 93
Thingnes (2019) 0.38 (0.09, 0.76) 8.33 8
Subtotal (I^2 = .%, p = .) 0.35 (0.25, 0.45) 100.00
PoC assay mobile: D site, S visit (2c)
Valencia (2020) 0.36 (0.29, 0.43) 100.00 197
PoC assay mobile: D site, NS visit (2d)
LJWG Homeless (2020) 0.61 (0.51, 0.69) 48.78 119
Lazarus (2020) 0.42 (0.33, 0.51) 51.22 125
Subtotal (I^2 = .%, p = .) 0.51 (0.45, 0.57) 100.00
PoC assay off site: S site, NS visit (3b)
Hamid (C1) (2019) 0.67 (0.64, 0.70) 18.41 950
HEADSTART Punjab (Markby) (C2) (2020) 0.81 (0.80, 0.83) 81.59 4211
Subtotal (I^2 = .%, p = .) 0.79 (0.78, 0.80) 100.00
PoC assay off site: D site, NS visit (3d)
Zhang (MSF) (2021) 0.71 (0.67, 0.74) 76.02 762
Agwuocha (2019) 0.55 (0.48, 0.61) 23.98 240
Subtotal (I^2 = .%, p = .) 0.67 (0.64, 0.70) 100.00
Non-PoC assay off site: S site, NS visit (4b)
MSF Karachi (Khalid) (C2) (2020) 0.62 (0.59, 0.65) 14.83 1038
MSF Phnom Penh (Walker) (C2) (2018) 0.69 (0.68, 0.71) 14.88 4969
Schurch (C2) (2020) 0.66 (0.58, 0.74) 14.47 142
Hamid (C2) (2019) 0.63 (0.59, 0.67) 14.80 632
Davies (C2) (2020) 0.65 (0.38, 0.86) 12.02 17
Mohamed (C2) (2020) 0.75 (0.64, 0.84) 14.18 83
Ustianowski (C2) (2020) 0.14 (0.12, 0.16) 14.82 806
Subtotal (I^2 = 99.41%, p = 0.00) 0.58 (0.40, 0.76) 100.00
Non-PoC assay off site: D site, NS visit (4d)
LJWG NSPs (C1) (2019) 0.78 (0.56, 0.93) 9.87 23
HEADSTART Georgia (Japaridze) (C2) (2020) 0.82 (0.79, 0.86) 34.64 485
HEADSTART Georgia (Japaridze) (C3) (2020) 0.82 (0.78, 0.85) 34.91 516
Martel-Laferriere (C2) (2019) 0.64 (0.53, 0.75) 20.59 76
Subtotal (I^2 = 75.02%, p = 0.01) 0.79 (0.72, 0.84) 100.00
0
.5
1

e)

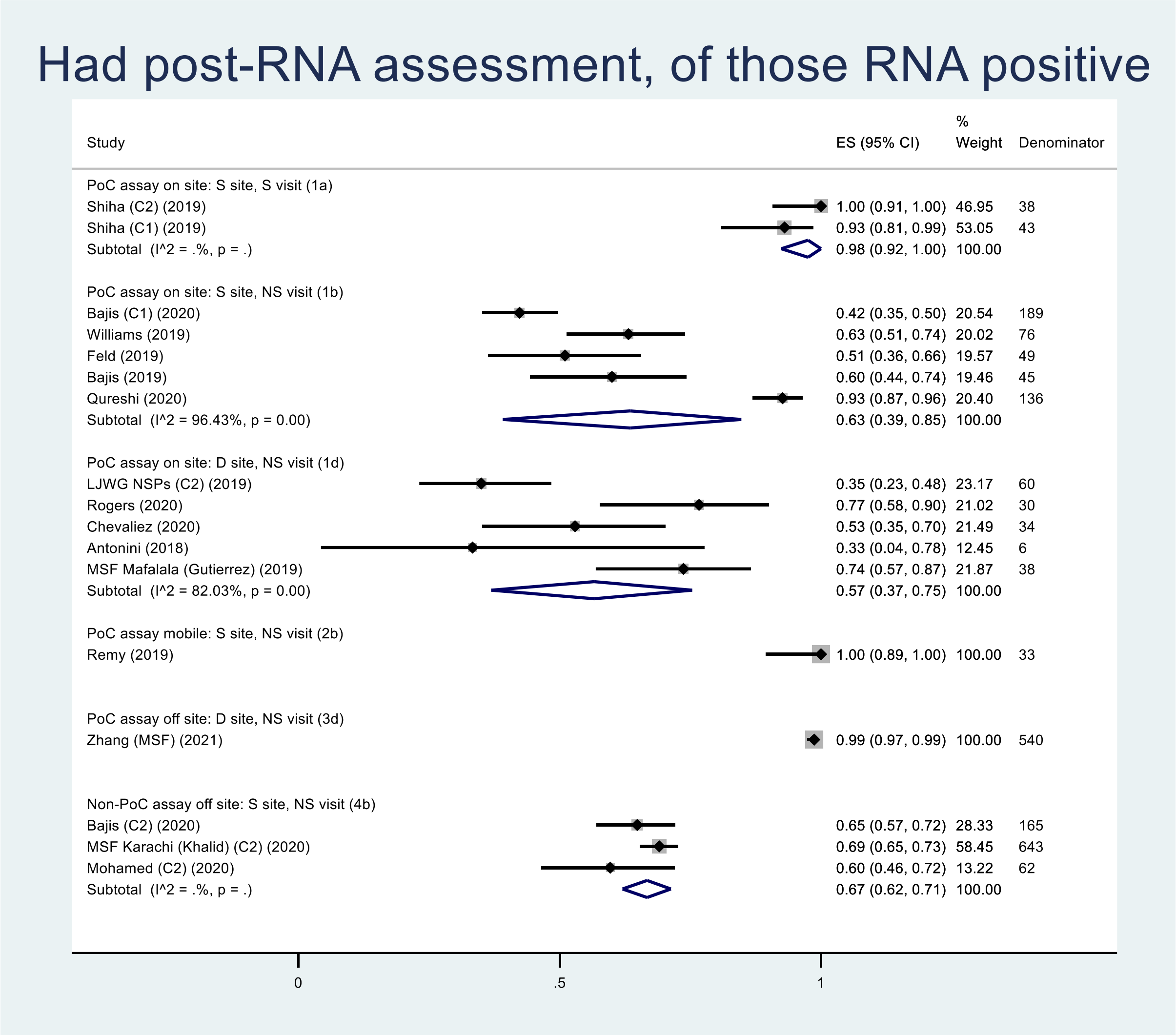
Had post-RNA assessment, of those RNA positive
Study
ES (95% CI)
% Weight
Denominator
PoC assay on site: S site, S visit (1a)
Shiha (C2) (2019) 1.00 (0.91, 1.00) 46.95 38
Shiha (C1) (2019) 0.93 (0.81, 0.99) 53.05 43
Subtotal (I^2 = .%, p = .) 0.98 (0.92, 1.00) 100.00
PoC assay on site: S site, NS visit (1b)
Bajis (C1) (2020) 0.42 (0.35, 0.50) 20.54 189
Williams (2019) 0.63 (0.51, 0.74) 20.02 76
Feld (2019) 0.51 (0.36, 0.66) 19.57 49
Bajis (2019) 0.60 (0.44, 0.74) 19.46 45
Qureshi (2020) 0.93 (0.87, 0.96) 20.40 136
Subtotal (I^2 = 96.43%, p = 0.00) 0.63 (0.39, 0.85) 100.00
PoC assay on site: D site, NS visit (1d)
LJWG NSPs (C2) (2019) 0.35 (0.23, 0.48) 23.17 60
Rogers (2020) 0.77 (0.58, 0.90) 21.02 30
Chevaliez (2020) 0.53 (0.35, 0.70) 21.49 34
Antonini (2018) 0.33 (0.04, 0.78) 12.45 6
MSF Mafalala (Gutierrez) (2019) 0.74 (0.57, 0.87) 21.87 38
Subtotal (I^2 = 82.03%, p = 0.00) 0.57 (0.37, 0.75) 100.00
PoC assay mobile: S site, NS visit (2b)
Remy (2019) 1.00 (0.89, 1.00) 100.00 33
PoC assay off site: D site, NS visit (3d)
Zhang (MSF) (2021) 0.99 (0.97, 0.99) 100.00 540
Non-PoC assay off site: S site, NS visit (4b)
Bajis (C2) (2020) 0.65 (0.57, 0.72) 28.33 165
MSF Karachi (Khalid) (C2) (2020) 0.69 (0.65, 0.73) 58.45 643
Mohamed (C2) (2020) 0.60 (0.46, 0.72) 13.22 62
Subtotal (I^2 = .%, p = .) 0.67 (0.62, 0.71) 100.00
0
.5
1

f)

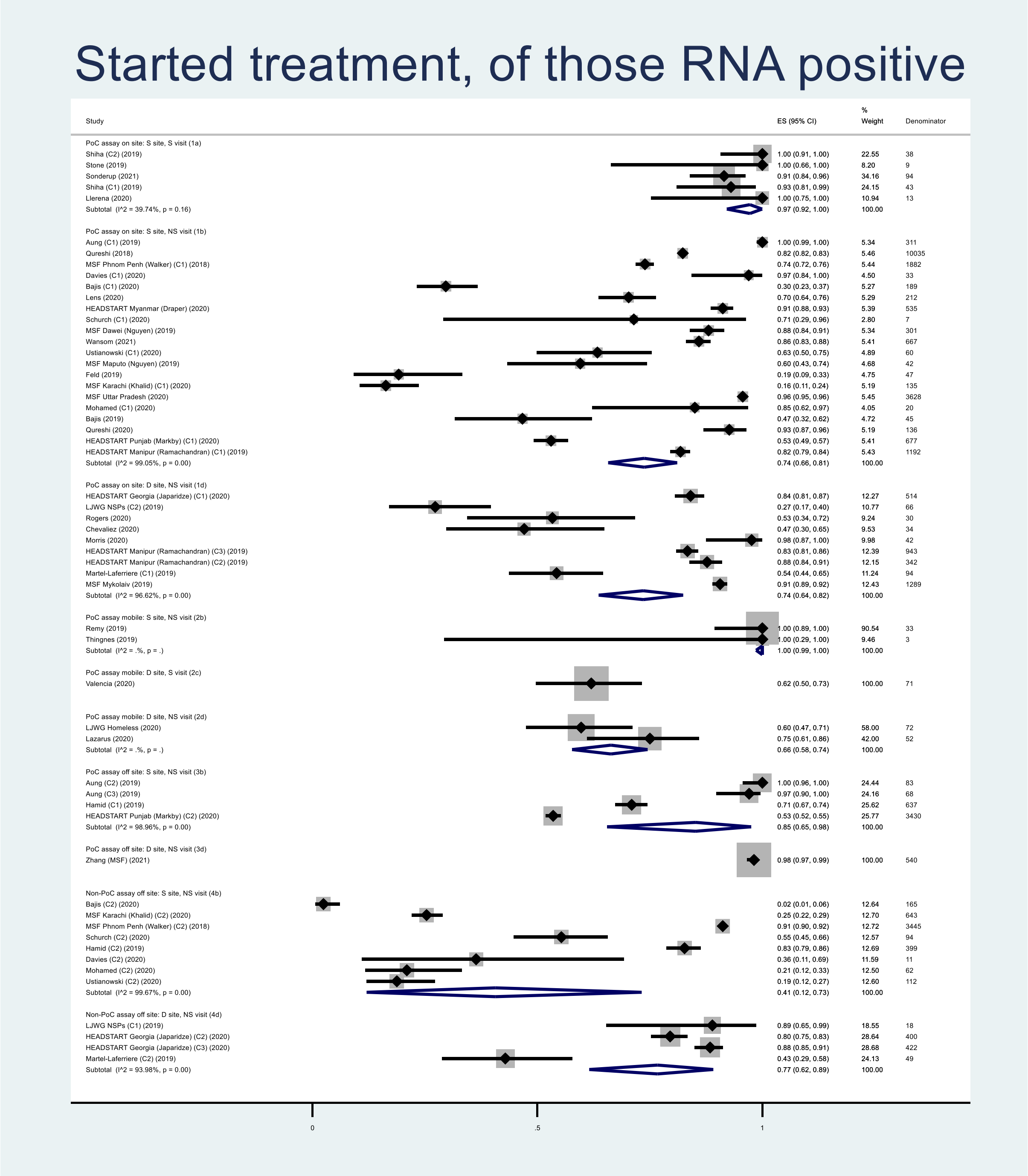
Started treatment, of those RNA positive
Study
ES (95% CI)
% Weight
Denominator
PoC assay on site: S site, S visit (1a)
Shiha (C2) (2019) 1.00 (0.91, 1.00) 22.55 38
Stone (2019) 1.00 (0.66, 1.00) 8.20 9
Sonderup (2021) 0.91 (0.84, 0.96) 34.16 94
Shiha (C1) (2019) 0.93 (0.81, 0.99) 24.15 43
Llerena (2020) 1.00 (0.75, 1.00) 10.94 13
Subtotal (I^2 = 39.74%, p = 0.16) 0.97 (0.92, 1.00) 100.00
PoC assay on site: S site, NS visit (1b)
Aung (C1) (2019) 1.00 (0.99, 1.00) 5.34 311
Qureshi (2018) 0.82 (0.82, 0.83) 5.46 10035
MSF Phnom Penh (Walker) (C1) (2018) 0.74 (0.72, 0.76) 5.44 1882
Davies (C1) (2020) 0.97 (0.84, 1.00) 4.50 33
Bajis (C1) (2020) 0.30 (0.23, 0.37) 5.27 189
Lens (2020) 0.70 (0.64, 0.76) 5.29 212
HEADSTART Myanmar (Draper) (2020) 0.91 (0.88, 0.93) 5.39 535
Schurch (C1) (2020) 0.71 (0.29, 0.96) 2.80 7
MSF Dawei (Nguyen) (2019) 0.88 (0.84, 0.91) 5.34 301
Wansom (2021) 0.86 (0.83, 0.88) 5.41 667
Ustianowski (C1) (2020) 0.63 (0.50, 0.75) 4.89 60
MSF Maputo (Nguyen) (2019) 0.60 (0.43, 0.74) 4.68 42
Feld (2019) 0.19 (0.09, 0.33) 4.75 47
MSF Karachi (Khalid) (C1) (2020) 0.16 (0.11, 0.24) 5.19 135
MSF Uttar Pradesh (2020) 0.96 (0.95, 0.96) 5.45 3628
Mohamed (C1) (2020) 0.85 (0.62, 0.97) 4.05 20
Bajis (2019) 0.47 (0.32, 0.62) 4.72 45
Qureshi (2020) 0.93 (0.87, 0.96) 5.19 136
HEADSTART Punjab (Markby) (C1) (2020) 0.53 (0.49, 0.57) 5.41 677
HEADSTART Manipur (Ramachandran) (C1) (2019) 0.82 (0.79, 0.84) 5.43 1192
Subtotal (I^2 = 99.05%, p = 0.00) 0.74 (0.66, 0.81) 100.00
PoC assay on site: D site, NS visit (1d)
HEADSTART Georgia (Japaridze) (C1) (2020) 0.84 (0.81, 0.87) 12.27 514
LJWG NSPs (C2) (2019) 0.27 (0.17, 0.40) 10.77 66
Rogers (2020) 0.53 (0.34, 0.72) 9.24 30
Chevaliez (2020) 0.47 (0.30, 0.65) 9.53 34
Morris (2020) 0.98 (0.87, 1.00) 9.98 42
HEADSTART Manipur (Ramachandran) (C3) (2019) 0.83 (0.81, 0.86) 12.39 943
HEADSTART Manipur (Ramachandran) (C2) (2019) 0.88 (0.84, 0.91) 12.15 342
Martel-Laferriere (C1) (2019) 0.54 (0.44, 0.65) 11.24 94
MSF Mykolaiv (2019) 0.91 (0.89, 0.92) 12.43 1289
Subtotal (I^2 = 96.62%, p = 0.00) 0.74 (0.64, 0.82) 100.00
PoC assay mobile: S site, NS visit (2b)
Remy (2019) 1.00 (0.89, 1.00) 90.54 33
Thingnes (2019) 1.00 (0.29, 1.00) 9.46 3
Subtotal (I^2 = .%, p = .) 1.00 (0.99, 1.00) 100.00
PoC assay mobile: D site, S visit (2c)
Valencia (2020) 0.62 (0.50, 0.73) 100.00 71
PoC assay mobile: D site, NS visit (2d)
LJWG Homeless (2020) 0.60 (0.47, 0.71) 58.00 72
Lazarus (2020) 0.75 (0.61, 0.86) 42.00 52
Subtotal (I^2 = .%, p = .) 0.66 (0.58, 0.74) 100.00
PoC assay off site: S site, NS visit (3b)
Aung (C2) (2019) 1.00 (0.96, 1.00) 24.44 83
Aung (C3) (2019) 0.97 (0.90, 1.00) 24.16 68
Hamid (C1) (2019) 0.71 (0.67, 0.74) 25.62 637
HEADSTART Punjab (Markby) (C2) (2020) 0.53 (0.52, 0.55) 25.77 3430
Subtotal (I^2 = 98.96%, p = 0.00) 0.85 (0.65, 0.98) 100.00
PoC assay off site: D site, NS visit (3d)
Zhang (MSF) (2021) 0.98 (0.97, 0.99) 100.00 540
Non-PoC assay off site: S site, NS visit (4b)
Bajis (C2) (2020) 0.02 (0.01, 0.06) 12.64 165
MSF Karachi (Khalid) (C2) (2020) 0.25 (0.22, 0.29) 12.70 643
MSF Phnom Penh (Walker) (C2) (2018) 0.91 (0.90, 0.92) 12.72 3445
Schurch (C2) (2020) 0.55 (0.45, 0.66) 12.57 94
Hamid (C2) (2019) 0.83 (0.79, 0.86) 12.69 399
Davies (C2) (2020) 0.36 (0.11, 0.69) 11.59 11
Mohamed (C2) (2020) 0.21 (0.12, 0.33) 12.50 62
Ustianowski (C2) (2020) 0.19 (0.12, 0.27) 12.60 112
Subtotal (I^2 = 99.67%, p = 0.00) 0.41 (0.12, 0.73) 100.00
Non-PoC assay off site: D site, NS visit (4d)
LJWG NSPs (C1) (2019) 0.89 (0.65, 0.99) 18.55 18
HEADSTART Georgia (Japaridze) (C2) (2020) 0.80 (0.75, 0.83) 28.64 400
HEADSTART Georgia (Japaridze) (C3) (2020) 0.88 (0.85, 0.91) 28.68 422
Martel-Laferriere (C2) (2019) 0.43 (0.29, 0.58) 24.13 49
Subtotal (I^2 = 93.98%, p = 0.00) 0.77 (0.62, 0.89) 100.00
0
.5
1

g)

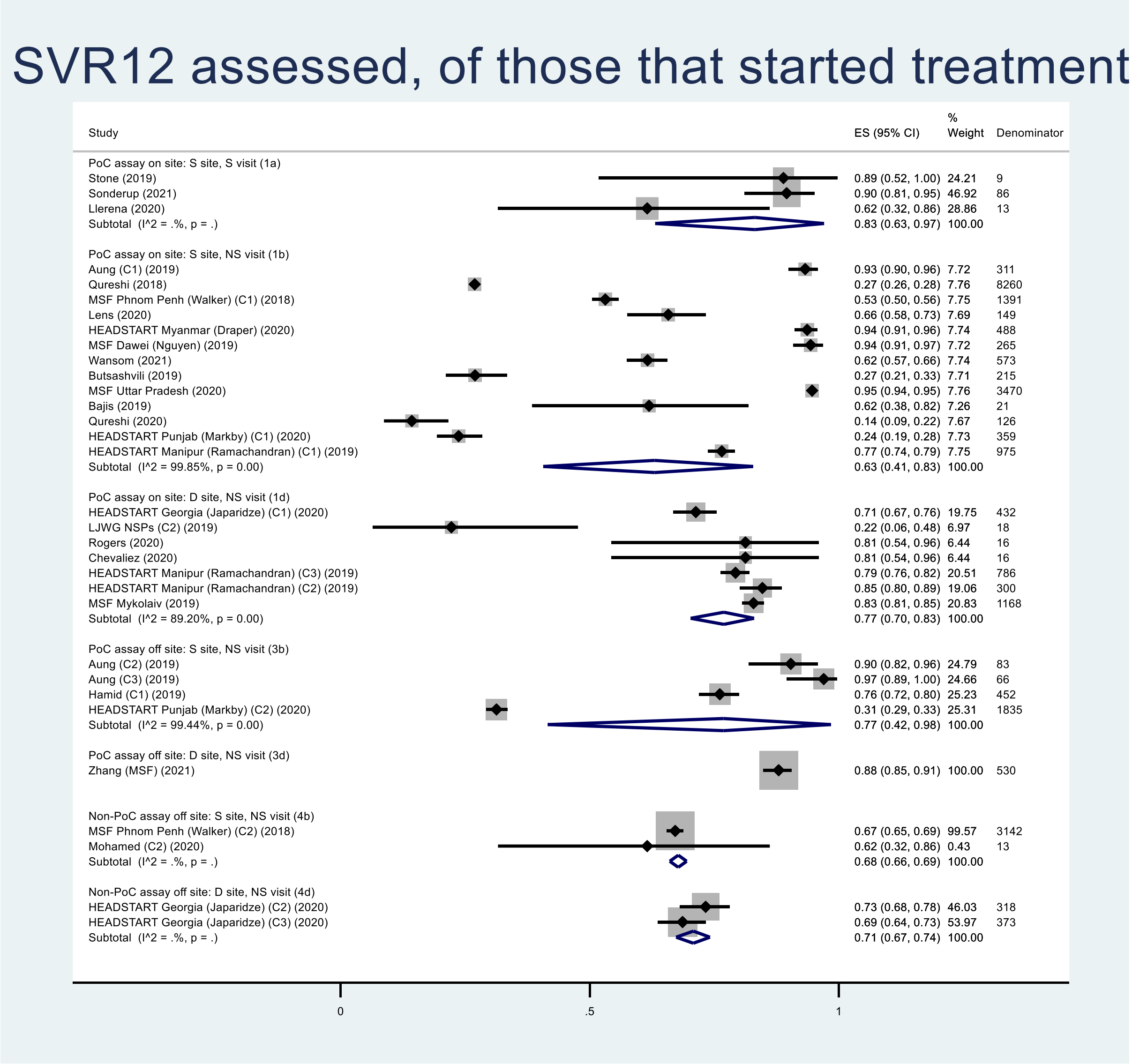

SVR12 assessed, of those that started treatment
Study
ES (95% CI)
% Weight
Denominator
PoC assay on site: S site, S visit (1a)
Stone (2019) 0.89 (0.52, 1.00) 24.21 9
Sonderup (2021) 0.90 (0.81, 0.95) 46.92 86
Llerena (2020) 0.62 (0.32, 0.86) 28.86 13
Subtotal (I^2 = .%, p = .) 0.83 (0.63, 0.97) 100.00
PoC assay on site: S site, NS visit (1b)
Aung (C1) (2019) 0.93 (0.90, 0.96) 7.72 311
Qureshi (2018) 0.27 (0.26, 0.28) 7.76 8260
MSF Phnom Penh (Walker) (C1) (2018) 0.53 (0.50, 0.56) 7.75 1391
Lens (2020) 0.66 (0.58, 0.73) 7.69 149
HEADSTART Myanmar (Draper) (2020) 0.94 (0.91, 0.96) 7.74 488
MSF Dawei (Nguyen) (2019) 0.94 (0.91, 0.97) 7.72 265
Wansom (2021) 0.62 (0.57, 0.66) 7.74 573
Butsashvili (2019) 0.27 (0.21, 0.33) 7.71 215
MSF Uttar Pradesh (2020) 0.95 (0.94, 0.95) 7.76 3470
Bajis (2019) 0.62 (0.38, 0.82) 7.26 21
Qureshi (2020) 0.14 (0.09, 0.22) 7.67 126
HEADSTART Punjab (Markby) (C1) (2020) 0.24 (0.19, 0.28) 7.73 359
HEADSTART Manipur (Ramachandran) (C1) (2019) 0.77 (0.74, 0.79) 7.75 975
Subtotal (I^2 = 99.85%, p = 0.00) 0.63 (0.41, 0.83) 100.00
PoC assay on site: D site, NS visit (1d)
HEADSTART Georgia (Japaridze) (C1) (2020) 0.71 (0.67, 0.76) 19.75 432
LJWG NSPs (C2) (2019) 0.22 (0.06, 0.48) 6.97 18
Rogers (2020) 0.81 (0.54, 0.96) 6.44 16
Chevaliez (2020) 0.81 (0.54, 0.96) 6.44 16
HEADSTART Manipur (Ramachandran) (C3) (2019) 0.79 (0.76, 0.82) 20.51 786
HEADSTART Manipur (Ramachandran) (C2) (2019) 0.85 (0.80, 0.89) 19.06 300
MSF Mykolaiv (2019) 0.83 (0.81, 0.85) 20.83 1168
Subtotal (I^2 = 89.20%, p = 0.00) 0.77 (0.70, 0.83) 100.00
PoC assay off site: S site, NS visit (3b)
Aung (C2) (2019) 0.90 (0.82, 0.96) 24.79 83
Aung (C3) (2019) 0.97 (0.89, 1.00) 24.66 66
Hamid (C1) (2019) 0.76 (0.72, 0.80) 25.23 452
HEADSTART Punjab (Markby) (C2) (2020) 0.31 (0.29, 0.33) 25.31 1835
Subtotal (I^2 = 99.44%, p = 0.00) 0.77 (0.42, 0.98) 100.00
PoC assay off site: D site, NS visit (3d)
Zhang (MSF) (2021) 0.88 (0.85, 0.91) 100.00 530
Non-PoC assay off site: S site, NS visit (4b)
MSF Phnom Penh (Walker) (C2) (2018) 0.67 (0.65, 0.69) 99.57 3142
Mohamed (C2) (2020) 0.62 (0.32, 0.86) 0.43 13
Subtotal (I^2 = .%, p = .) 0.68 (0.66, 0.69) 100.00
Non-PoC assay off site: D site, NS visit (4d)
HEADSTART Georgia (Japaridze) (C2) (2020) 0.73 (0.68, 0.78) 46.03 318
HEADSTART Georgia (Japaridze) (C3) (2020) 0.69 (0.64, 0.73) 53.97 373
Subtotal (I^2 = .%, p = .) 0.71 (0.67, 0.74) 100.00
0
.5
1

h)

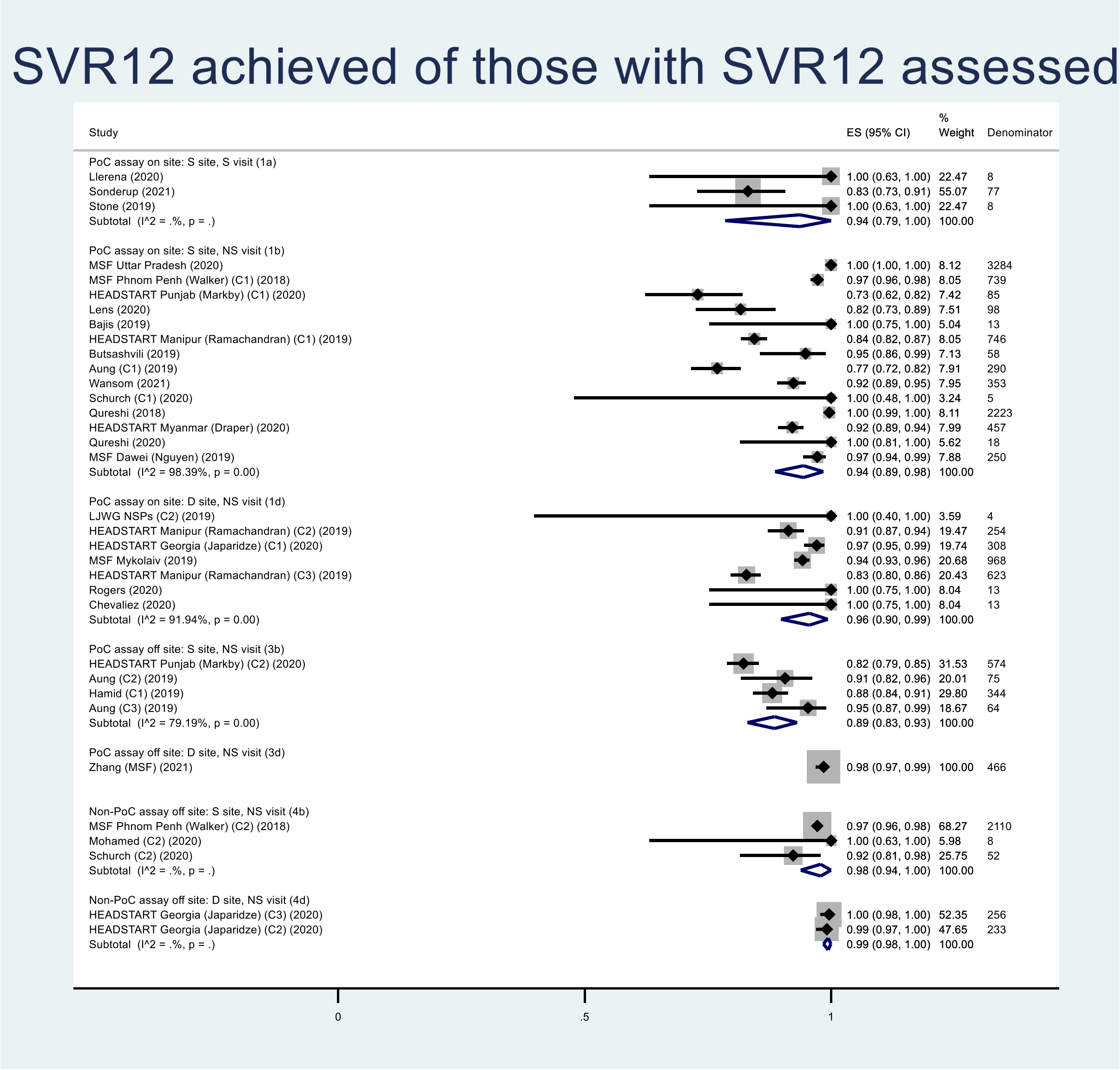
SVR12 achieved of those with SVR12 assessed
Study
ES (95% CI)
% Weight
Denominator
PoC assay on site: S site, S visit (1a)
Llerena (2020) 1.00 (0.63, 1.00) 22.47 8
Sonderup (2021) 0.83 (0.73, 0.91) 55.07 77
Stone (2019) 1.00 (0.63, 1.00) 22.47 8
Subtotal (I^2 = .%, p = .) 0.94 (0.79, 1.00) 100.00
PoC assay on site: S site, NS visit (1b)
MSF Uttar Pradesh (2020) 1.00 (1.00, 1.00) 8.12 3284
MSF Phnom Penh (Walker) (C1) (2018) 0.97 (0.96, 0.98) 8.05 739
HEADSTART Punjab (Markby) (C1) (2020) 0.73 (0.62, 0.82) 7.42 85
Lens (2020) 0.82 (0.73, 0.89) 7.51 98
Bajis (2019) 1.00 (0.75, 1.00) 5.04 13
HEADSTART Manipur (Ramachandran) (C1) (2019) 0.84 (0.82, 0.87) 8.05 746
Butsashvili (2019) 0.95 (0.86, 0.99) 7.13 58
Aung (C1) (2019) 0.77 (0.72, 0.82) 7.91 290
Wansom (2021) 0.92 (0.89, 0.95) 7.95 353
Schurch (C1) (2020) 1.00 (0.48, 1.00) 3.24 5
Qureshi (2018) 1.00 (0.99, 1.00) 8.11 2223
HEADSTART Myanmar (Draper) (2020) 0.92 (0.89, 0.94) 7.99 457
Qureshi (2020) 1.00 (0.81, 1.00) 5.62 18
MSF Dawei (Nguyen) (2019) 0.97 (0.94, 0.99) 7.88 250
Subtotal (I^2 = 98.39%, p = 0.00) 0.94 (0.89, 0.98) 100.00
PoC assay on site: D site, NS visit (1d)
LJWG NSPs (C2) (2019) 1.00 (0.40, 1.00) 3.59 4
HEADSTART Manipur (Ramachandran) (C2) (2019) 0.91 (0.87, 0.94) 19.47 254
HEADSTART Georgia (Japaridze) (C1) (2020) 0.97 (0.95, 0.99) 19.74 308
MSF Mykolaiv (2019) 0.94 (0.93, 0.96) 20.68 968
HEADSTART Manipur (Ramachandran) (C3) (2019) 0.83 (0.80, 0.86) 20.43 623
Rogers (2020) 1.00 (0.75, 1.00) 8.04 13
Chevaliez (2020) 1.00 (0.75, 1.00) 8.04 13
Subtotal (I^2 = 91.94%, p = 0.00) 0.96 (0.90, 0.99) 100.00
PoC assay off site: S site, NS visit (3b)
HEADSTART Punjab (Markby) (C2) (2020) 0.82 (0.79, 0.85) 31.53 574
Aung (C2) (2019) 0.91 (0.82, 0.96) 20.01 75
Hamid (C1) (2019) 0.88 (0.84, 0.91) 29.80 344
Aung (C3) (2019) 0.95 (0.87, 0.99) 18.67 64
Subtotal (I^2 = 79.19%, p = 0.00) 0.89 (0.83, 0.93) 100.00
PoC assay off site: D site, NS visit (3d)
Zhang (MSF) (2021) 0.98 (0.97, 0.99) 100.00 466
Non-PoC assay off site: S site, NS visit (4b)
MSF Phnom Penh (Walker) (C2) (2018) 0.97 (0.96, 0.98) 68.27 2110
Mohamed (C2) (2020) 1.00 (0.63, 1.00) 5.98 8
Schurch (C2) (2020) 0.92 (0.81, 0.98) 25.75 52
Subtotal (I^2 = .%, p = .) 0.98 (0.94, 1.00) 100.00
Non-PoC assay off site: D site, NS visit (4d)
HEADSTART Georgia (Japaridze) (C3) (2020) 1.00 (0.98, 1.00) 52.35 256
HEADSTART Georgia (Japaridze) (C2) (2020) 0.99 (0.97, 1.00) 47.65 233
Subtotal (I^2 = .%, p = .) 0.99 (0.98, 1.00) 100.00
0
.5
1

*OUTCOMES ACROSS OTHER CASCADE OF CARE STEPS*

*HCV Antibody testing uptake*: Supplementary table 10 shows that few data were available on the uptake of anti-HCV testing (mostly available for studies among prisoners) with wide confidence intervals for the pooled proportion of uptake among arms where a non-PoC RNA assay was located off site (59% [95%CI: 18%-93%]: 3 arms, n=6356) and where a PoC RNA assay was located on site (77% [95%CI: 58%-92%]), indicating little evidence of differences between PoC compared to lab-based assays (p=0.42). However, there was evidence that among PoC RNA assay arms on site, model 1a had higher anti-HCV testing uptake (91% [95%CI: 88%-94%]: 1 arm, n=425) than model 1b (73% [95%CI: 54%-88%]: 4 arms, n=2320), p-value=0.01.

*SVR12 assessment uptake:* When looking at the proportion of treated patients that were assessed for SVR12, there was little evidence of differences by RNA assay type and location, or the model of care sub-categories regarding co-location of testing and treatment.

*Percentage HCV antibody and HCV RNA positive:* There was variation in the proportion of patients that were HCV antibody positive by model of care type, however, this mostly reflected the heterogeneity in the populations tested and is presented in the table, as is HCV RNA positivity, to capture patient flow through the cascade. There was no evidence of differences in HCV RNA positivity when comparing models 1 and 2 vs model 4 (p=0.49), models 1 and 2 vs model 3 (p=0.17), or model 3 vs model 4 (p=0.65).

*Attaining SVR12:* Among those assessed for SVR12, the proportion achieving SVR12 was generally similar across studies. Although a few other arms had similarly low SVR12 proportions, these arms had less influence on the results within their model of care categorisation. The percentage obtaining SVR12 when PoC RNA assays were on site was 95% (95%CI: 91%-98%) (24 arms, n=10895), whilst the percentage for lab-based PoC RNA assay arms was 92% (95%CI: 83%-98%) (5 arms, n=1523), and for lab-based high throughput RNA assay arms it was 99% (95%CI: 97%-100%) (5 arms, n=2659).

COMPARING OUTCOMES ACROSS COUNTRY INCOME GROUPS

For general-population, prisoners, and PWH comparing outcomes across country income groups was not possible as 100% of the general population studies are from low- and middle-income countries (LMICs), as were 100% of studies among PLHIV, whilst 100% of prisoner studies are from high-income countries (HICs). This left the studies among PWID and homeless populations that were split between the country-income groups.

For RNA testing uptake, there was overlap between the high-income countries and LMICs:

- High income: 84% (63%-98%)
- Low/middle income: 94% (81%-100%)

For treatment uptake among PWID populations, higher uptake was seen in the studies in LMICs:

- High income: 60% (45%-75%)
- Low/middle income: 91% (86%-94%)

The distribution of the different model types is similar for both HICs and LMICs. One difference between the two groups is that the HIC studies are much smaller – mean denominator for treatment uptake is 67, which is 464 for the LMIC studies. In general these LMIC PWID studies are from large organisations like MSF, FIND and EQUIP, whilst the HIC PWID studies are generally from smaller organisations or individual research centers.

**Supplementary table 12:** Test for publication bias* for each cascade outcome proportion using a regression-based Egger test (Random-effects model)

| **Outcome** | **Egger test p-value** |
|---|---|
| | |
| Proportion RNA tested | 0.6694 |
| Proportion treated | 0.7553 |
| Proportion SVR assessed | 0.8874 |
| Proportion achieving SVR12 | 0.7853 |

*Also known as tests for small study effects

**Grading the quality of evidence**

Quality of evidence assessments were based on the following domains:

- Risk of bias, based on the overall risk of bias across studies
- Consistency of results across studies
- Directness of the evidence linking the intervention and health outcomes and directness of comparisons
- Precision of the estimate of effect, based on the number and size of studies and confidence intervals for the estimates
- Reporting bias, based on whether the studies defined and reported primary outcomes and whether we identified relevant unpublished studies

Based on our assessments of the domains described above, we graded the quality of evidence for each intervention as high, moderate, low, or very low. Randomized controlled trials of interventions start as "high" quality of evidence and are graded down based on the presence and severity of shortcomings in each domain. A "high" grade indicates high confidence that the evidence reflects the true effect, and that further research is very unlikely to change our confidence in the estimate of effect. A "moderate" grade indicates moderate confidence that the evidence reflects the true effect and further research may change the estimate. A "low" grade indicates low confidence that the evidence reflects the true effect and further research is likely to change the confidence in the estimate of effect and could increase the confidence in the estimate. A "very low" grade indicates evidence either is unavailable or is too limited to permit any conclusion, due to extreme study limitations, inconsistency, imprecision, or reporting bias.

**Supplementary table 13:** Grading the quality of evidence for time to event data, using the indirect pooled comparisons of PoC RNA assays with lab-based RNA assays.

| Outcome | Evidence | Risk of bias | Inconsistency | Imprecision | Indirectness | QOE | Main findings |
|---|---|---|---|---|---|---|---|
| Time from antibody test to RNA test | 22 study arms from observational studies (N=14,005) | Serious risk of bias (10/22 arms with high or moderate risk of bias) | No inconsistency | No imprecision | Serious indirectness* | Low | No difference between models of care and time from Ab test to RNA test |
| Time from RNA sample collection to test | 14 study arms from observational studies (N=9,963) | Serious risk of bias (6/14 arms with high or moderate risk of bias) | No inconsistency | No imprecision | Serious indirectness* | Low | PoC RNA assay on site associated with 5.9 fewer days from RNA sample collection to test vs. lab-based high-throughput RNA assayǂ |
| Time from RNA test to results made available | 19 studies arms from observational studies (N=12,017) | Serious risk of bias (11/19 arms with high or moderate risk of bias) | No inconsistency | No imprecision | Serious indirectness* | Low | PoC RNA assay on site associated with 4 fewer days from RNA test to results made available vs. lab-based high-throughput RNA assayǂ |
| Time from RNA test to treatment start | 28 study arms from observational studies (N=7,497) | Serious risk of bias (16/28 arms with high or moderate risk of bias) | No inconsistency | No imprecision | Serious indirectness* | Low | PoC RNA assay on site associated with 49 fewer days from RNA test to treatment start vs. lab-based PoC RNA assay and 30 fewer days to treatment start vs. lab-based high-throughput RNA assay† |
| Time from antibody test to treatment start | 14 study arms from observational studies (N=5,640) | Serious risk of bias (5/14 arms with high or moderate risk of bias) | No inconsistency | No imprecision | Serious indirectness* | Low | PoC RNA assay on site associated with 45.5 fewer days from Ab test to treatment start vs. lab-based PoC RNA assay and 48.5 fewer days to treatment start vs. lab-based high-throughput RNA assay |

QOE: Quality of evidence. Ab: Antibody. PoC: Point of care

* Few studies directly compared models of care, so most findings are primarily based on indirect (across study) comparison. However, the limited head-to-head data were generally consistent with the indirect evidence. ǂ There is limited evidence (1 study each) for models of care 2 (PoC RNA assay mobile) and 3 (Lab-based PoC RNA assay). † There is limited evidence (1 study each) for model of care 2 (PoC RNA assay mobile). ǂ There is very limited evidence (1 small study) for model of care 2 (PoC RNA assay mobile) and limited evidence (1 study) for model of care 3 (Lab-based PoC RNA assay).

**Supplementary table 14:** Grading the quality of evidence for the proportion of people with uptake of RNA testing and HCV treatment, comparing PoC RNA assays with lab-based RNA assays.

| Outcome | Evidence | Risk of bias | Inconsistency | Imprecision | Indirectness | QOE | Main findings |
|---|---|---|---|---|---|---|---|
| RNA testing uptake proportion | 8 study arms from observational studies (N=2430) | Serious risk of bias (6/8 arms with high or moderate risk of bias) | Serious inconsistency ($I^2$: 91.4%) | Serious imprecision* | No indirectness | Low | Relative risk: 1.11 (95% confidence interval: 0.89 to 1.38) |
| HCV treatment uptake proportion | 21 study arms from observational studies (N=8391) | Serious risk of bias (16/21 arms with high or moderate risk of bias) | Serious inconsistency ($I^2$: 95.8%) | No imprecision | No indirectness | Low | Relative risk: 1.32 (95% confidence interval: 1.06 to 1.64) |

*95% confidence intervals crosses 1.

## References


1. Liberati A, Altman DG, Tetzlaff J, et al. The PRISMA statement for reporting systematic reviews and meta-analyses of studies that evaluate healthcare interventions: explanation and elaboration. *Brit Med J* 2009; **339**: b2700.
2. UNITAID. Hepatitis C Diagnostic Technology Landscape, 2019. https://marketbookshelf.com/wp-content/uploads/2019/07/HepC-Dx-Tech-Landscape_May2019.pdf (accessed 03/10/2022).
3. World Health Organization. WHO Prequalification of In Vitro Diagnostics - Product: Xpert HCV Viral Load with GeneXpert Dx, GeneXpert Infinity-48s, and GeneXpert Infinity-80 WHO reference number: PQDx 0260-070-00. Geneva, Switzerland: World Health Organization, 2017. https://extranet.who.int/pqweb/sites/default/files/PQDx0260-070-00_XpertHCV-Viral-Load_v4.0.pdf (accessed 03/10/2022).
4. Hoy D, Brooks P, Woolf A, et al. Assessing risk of bias in prevalence studies: modification of an existing tool and evidence of interrater agreement. *J Clin Epidemiol* 2012; **65**(9): 934-9.
5. Sterne JAC, Hernan MA, Reeves BC, et al. ROBINS-I: a tool for assessing risk of bias in non-randomised studies of interventions. *Brit Med J* 2016; **355**.
6. Le Roux S, Meyer L, Vojnov L. Clinical and operational impact of point-of-care compared to laboratory-based nucleic acid testing for routine HIV viral load monitoring: a systematic review and meta-analysis. In: Updated recommendations on HIV prevention, infant diagnosis, antiretroviral initiation and monitoring. Geneva, Switzerland: World Health Organization, 2021. http://apps.who.int/iris/handle/10665/340190 (accessed 05/10/2022).
7. Bajis S, Grebely J, Cooper L, et al. Hepatitis C virus testing, liver disease assessment and direct-acting antiviral treatment uptake and outcomes in a service for people who are homeless in Sydney, Australia: The LiveRLife homelessness study. *Journal of Viral Hepatitis* 2019; **26**(8): 969-79.
8. London Joint Working Group on Substance Use and Hepatitis C. Hepatitis C testing and treatment interventions for the homeless population in London during the Covid-19 pandemic: Outcomes and learning, 2020. http://ljwg.org.uk/wp-content/uploads/2021/01/HCV-testing-in-temporary-accommodation-during-lockdown-December-2020.pdf (accessed 03/10/2022).
9. Chevaliez S, Wlassow M, Volant J, et al. Assessing Molecular Point-of-Care Testing and Dried Blood Spot for Hepatitis C Virus Screening in People Who Inject Drugs. *Open Forum Infectious Diseases* 2020; **7**(6): 6-.
10. Lens S, Miralpeix A, Galvez M, et al. Externalized HCV linkage-to-care cascade in the biggest harm reduction center in Barcelona: approaching a high-risk PWID population. *J Hepatol* 2020; **73**: S360-S.
11. Lazarus J, Ovrehus A, Demant J, Krohn-Hehli L, Weis N. A novel hepatitis C intervention in Denmark to test and treat people who inject drugs. *J Hepatol* 2020; **73**: S306-S.
12. Rogers B, Spear J, Mistry V, Wiselka M, Pareek M. Pharmacy-based molecular point-of-care testing for hepatitis C (HCV) in high-risk patients: feasibility and linkage to care. *J Hepatol* 2020; **73**: S307-S.
13. Antonini TM, Deschenau A, Le Pape S, Tateo MG, Roque-Afonso AM. Feasibility of a Global Out of Wall Assessment Of Hepatitis C Liver Disease In a Drug Service. 7th International Symposium on Hepatitis Care in Substance Users; Sept 19–21, 2018 (abstr); Cascais, Portugal.
14. Remy AJ, Hakim B, Hervet J, Happiette A. Test to cure: Increase outreach linkage to care by use of real time HCV viral load. *J Hepatol* 2019; **70**(1): E504-E.
15. Bajis S, Grebely J, Hajarizadeh B, et al. Hepatitis C virus testing, liver disease assessment and treatment uptake among people who inject drugs pre- and post-universal access to direct-acting antiviral treatment in Australia: The LiveRLife study. *Journal of Viral Hepatitis* 2020; **27**(3): 281-93.
16. Williams B, Howell J, Doyle J, et al. Point-of-care hepatitis C testing from needle and syringe programs: An Australian feasibility study. *International Journal of Drug Policy* 2019; **72**: 91-8.
17. Valencia J, Gutierrez J, Troya J, Cuevas C, Ryan P. Addressing the HCV cascade of care in vulnerable populations with poor access to healthcare in Madrid through of a point of care in a one

step. 9th International Symposium on Hepatitis Care in Substance Users; Oct 13-15, 2021 (abstr); Sydney, Australia. https://www.inhsu.org/resource/addressing-the-hcv-cascade-of-care-in-vulnerable-populations-with-poor-access-to-healthcare-in-madrid-through-of-a-point-of-care-in-a-one-step/ (accessed 06/10/2022).
18. Ryan P, Valencia J, Cuevas G, et al. Detection of active hepatitis C in a single visit and linkage to care among marginalized people using a mobile unit in Madrid, Spain. *International Journal of Drug Policy* 2021; **96**.
19. Saludes V, Antuori A, Lazarus JV, et al. Evaluation of the Xpert HCV VL Fingerstick point-of-care assay and dried blood spot HCV-RNA testing as simplified diagnostic strategies among people who inject drugs in Catalonia, Spain. *International Journal of Drug Policy* 2020; **80**: 9-.
20. Schürch S, Fux CA, Dehler S, et al. Management of hepatitis C in opioid agonist therapy patients of the Swiss canton Aargau within and outside the cohort study. *Swiss medical weekly* 2020; **150**: w20317.
21. Martel-Laferriere V, Brissette S, Juteau LC, et al. Effect of a single-day investigation for HCV infection on treatment initiation among people who inject drugs (PWID). *Hepatology v70 suppl1 2019* 2019; **70 (Supplement 1)**: 953A-4A.
22. Martel-Laferriere V, Brissette S, Wartelle-Bladou C, et al. Impact of an Accelerated Pretreatment Evaluation on Linkage-to-Care for Hepatitis C-infected Persons Who Inject Drugs. *Subst Abus-Res Treat* 2022; **16**.
23. Feld JJ, Lettner B, Mason K, et al. Rapid hepatitis c point-of-care RNA testing and treatment at an integrated supervised consumption site in Toronto, Canada. *Hepatology v70 suppl1 2019* 2019; **70 (Supplement 1)**: 973A-4A.
24. Thingnes GS, Ulstein K, Dalgard O. Challenges in delivery of point of care testing for HCV RNA in a mobile health service for people who inject drugs. 8th International Symposium on Hepatitis Care in Substance Users; Sept 11-13, 2019 (abstr); Montreal, Canada.
25. Midgard H, Bjornestad R, Egeland M, et al. Peer support in small towns: A decentralized mobile Hepatitis C virus clinic for people who inject drugs. *Liver International* 2022; **42**(6): 1268-77.
26. Stone B, Emerson S, Jenkins K, et al. Hepatitis C Single Attendance Test Assess and Treat ("Hep STAT") minimizes care cascade attrition and achieves high rates of treatment initiation, completion and cure in people who inject drugs. 9th International Symposium on Hepatitis Care in Substance Users; Oct 13-15, 2021 (abstr); Sydney, Australia. https://www.inhsu.org/resource/hepatitis-c-single-attendance-test-assess-and-treat-hep-stat-minimizes-care-cascade-attrition-and-achieves-high-rates-of-treatment-initiation-completion-and-cure-in-people-who-inj/ (accessed 06/10/2022).
27. Gutierrez AG, Medicins Sans Frontiers. The Mafalala Pilot: Institutional and Community Intervention for HIV, Hepatitis and TB Prevention and Care for People Who Use Drugs. 20th International Conference on AIDS and STIs in Africa (ICASA); Dec 2-7, 2019 (abstr); Kigali, Rwanda.
28. Butsashvili M, Kamkamidze G, Kajala M, et al. Hepatitis C Treatment Integration with Harm Reduction Services in Georgia: Preliminary Findings. 8th International Symposium on Hepatitis Care in Substance Users; Sept 11-13, 2019 (abstr); Montreal, Canada.
29. Thaung YM, Chasela CS, Chew KW, et al. Treatment outcomes and costs of a simplified antiviral treatment strategy for hepatitis C among monoinfected and HIV and/or hepatitis B virus infected patients in Myanmar. *Journal of Viral Hepatitis* 2021; **28**(1): 147-58.
30. Ramachandran BE, Sarin S, Shilton S, et al. Community centric HCV testing and treatment for PWID in Manipur, India- the HEAD START project. 8th International Symposium on Hepatitis Care in Substance Users; Sept 11-13, 2019 (abstr); Montreal, Canada.
31. Japaridze M, Markby J, Khonelidze I, Butsashvili M, Alkhazashvili M, Shilton S. The HEAD-START project Georgia: a three-armed, cluster, nonrandomised trial of the effectiveness of two novel models of HCV confirmatory testing in harm reduction sites (HRS) in Georgia. *J Hepatol* 2020; **73**: S841-S.

32. Shilton S, Markby J, Japaridze M, et al. Feasibility and effectiveness of HCV viraemia testing at harm reduction sites in Georgia: A prospective three-arm study. *Liver International* 2022; **42**(4): 775-86.
33. Hellard M. Community-based point-of-care hepatitis C testing and general practitioner initiated direct-acting antiviral therapy in Yangon, Myanmar (CT2 study). *Hepatology International* 2020; **14 (Supplement)**: S136-S7.
34. Draper BL, Htay H, Pedrana A, et al. Outcomes of the CT2 study: A 'one-stop-shop' for community-based hepatitis C testing and treatment in Yangon, Myanmar. *Liver International* 2021; **41**(11): 2578-89.
35. London Joint Working Group on Substance Use and Hepatitis C. HCV testing in NSP (Needle and Syringe Provision) Community Pharmacies Pilot (Phase 1), 2018. http://ljwg.org.uk/wp-content/uploads/2018/05/LJWG-Pharmacy-Testing-Phase-1-final-report-.pdf (accessed 03/10/2022).
36. London Joint Working Group on Substance Use and Hepatitis C. HCV testing in NSP (Needle and Syringe Provision) Community Pharmacies Pilot (Phase 2), 2019. http://ljwg.org.uk/wp-content/uploads/2019/09/LJWG-HCV-phase-2-report-FINAL.pdf (accessed 03/10/2022).
37. SOS Hepatites BFC team. Le Road Trip Hepatant. 2019. https://mailchi.mp/19037c30b944/vice-versa-n25-novembre-2019?fbclid=IwAR39VjeWpO6rVQqER8FVQqj3DHyvE_sjt7p-B1PgScxDwXDQ2eI3653NWEY (accessed 04/01/2021).
38. Morris D, Bonnett P, Smith S. Hepatitis C Test and Treat Roadshow - Reaching Homeless Communities across the West Midlands, UK. *Gut* 2020; **69**: A3-A4.
39. Wansom T, Thongmee A, Chittmittrapap S, et al. High HCV cure rates in C-FREE, first community-based study offering testing and treatment of viral hepatitis and HIV among people who use drugs and their partners in Thailand. *J Int Aids Soc* 2021; **24**: 17-8.
40. Sonderup MW. Access to HCV Treatment for People Who Inject Drugs through a Simplified Model of Care in Pretoria. Sysmex Asia Pacific Meeting 2021; 2021; Virtual.
41. Agwuocha C, Sacks J, Boeke C, et al. Use of facility-based provider initiated testing and counselling approach to ascertain viral hepatitis C status in high burden populations in Nigeria. *J Hepatol* 2019; **70**(1): E322-E3.
42. Médicins Sans Frontieres. Meerut Hepatitis-C Project. A Stakeholder's Consultation on Simplified model of care for HCV in India with focus on Uttar Pradesh; 03/02/2020; New Delhi, India.
43. Walker JG, Mafirakureva N, Iwamoto M, et al. Cost and cost-effectiveness of a simplified treatment model with direct-acting antivirals for chronic hepatitis C in Cambodia. *Liver International* 2020; **40**(10): 2356-66.
44. Khalid GG, Kyaw KWY, Bousquet C, et al. From risk to care: the hepatitis C screening and diagnostic cascade in a primary health care clinic in Karachi, Pakistan-a cohort study. *Int Health* 2020; **12**(1): 19-27.
45. Qureshi H, Riaz MK, Mahmood H, et al. The report of collaboration of a Civil Society Organization (CSO) and private gastroenterologist in diagnosis and treatment of patients with Hepatitis C Virus (HCV) infection. World Hepatitis Summit 2017; Nov 1-3, 2017 (abstr); Sao Paulo, Brazil. https://www.researchgate.net/publication/327361934_The_report_on_diagnosis_and_treatment_of_patients_with_Hepatitis_C_Virus_HCV_infection (accessed 05/10/2022).
46. Qureshi H. Hepatitis C Elimination in Pakistan Opportunities and Challenges. International Liver Conference 2019; Apr 10-14, 2019 (abstr); Vienna, Austria.
47. Rewari BB. Community based models of care and Testing strategies in high burden areas - perspective. A Stakeholder's Consultation on Simplified model of care for HCV in India with focus on Uttar Pradesh; 03/02/2020; New Delhi, India.
48. Hamid S, Abid A, Dore GJ. Identifying the lower age limit for hepatitis C screening in large elimination programmes in highly endemic areas. *Lancet Gastroenterol* 2021; **6**(2): 89-90.

49. Abid A, Uddin M, Muhammad T, et al. Evaluation of Hepatitis C Virus Core Antigen Assay in a Resource-Limited Setting in Pakistan. *Diagnostics* 2021; **11**(8).
50. Shiha G, Soliman R, Serwah A, Mikhail NNH, Asselah T, Easterbrook P. A same day 'test and treat' model for chronic HCV and HBV infection: Results from two community-based pilot studies in Egypt. *Journal of Viral Hepatitis* 2020; **27**(6): 593-601.
51. Zhang M, O'Keefe D, Craig J, et al. Decentralised hepatitis C testing and treatment in rural Cambodia: evaluation of a simplified service model integrated in an existing public health system. *Lancet Gastroenterol Hepatol* 2021; **6**(5): 371-80.
52. Mohamed Z, Al-Kurdi D, Nelson M, et al. Time matters: Point of care screening and streamlined linkage to care dramatically improves hepatitis C treatment uptake in prisoners in England. *International Journal of Drug Policy* 2020; **75**: 8.
53. Davies L, Healy B, Matthews G, et al. Elimination of hepatitis C in a remand prison using a rapid point of care driven test and treat pathway. *J Hepatol* 2020; **73**: S352-S.
54. Llerena S, Cabezas J, Mateo M, et al. Microelimination beyond prison walls: subjects sentenced to non-custodial sentences, screening and immediate assisted treatment with "navigator" figure and telemedicine. *J Hepatol* 2020; **73**: S361-S2.
55. Cabezas J, Llerena S, Mateo M, et al. Hepatitis C Micro-Elimination beyond Prison Walls: Navigator-Assisted Test-and-Treat Strategy for Subjects Serving Non-Custodial Sentences. *Diagnostics* 2021; **11**(5).
56. Ustianowski A, White M, Bell S, et al. Rapid test & treat programme successfully facilitating hepatitis C micro-elimination in a women's prison. *J Hepatol* 2020; **73**: S793-S.
57. Shilton S, Grover GS, Gupta E, et al. Demonstration of the feasibility of two innovative models of HCV testing and treatment in two unique populations in Punjab and Delhi, India-head-start project, India. *J Hepatol* 2020; **73**: S828-S.
58. Nguyen A, Fontas C, Tamayo Antabak N, Molfino L, Incerti A, Loarec A. Feasibility of treating HCV in low resource settings: The MSF projects in Mozambique and Myanmar. EASL Monothematic Conference on Translational research in viral hepatitis; Jan 23-25, 2020 (abstr); Athens, Greece.
59. Nguyen A, (MSF). Médicins Sans Frontieres. Personal Communication. 2018.
